# Mapping the Initial Stages of a Protective Pathway that Enhances Catalytic Turnover by a Lytic Polysaccharide Monooxygenase

**DOI:** 10.1021/jacs.3c06607

**Published:** 2023-09-09

**Authors:** Jingming Zhao, Ying Zhuo, Daniel E. Diaz, Muralidharan Shanmugam, Abbey J. Telfer, Peter J. Lindley, Daniel Kracher, Takahiro Hayashi, Lisa S. Seibt, Florence J. Hardy, Oliver Manners, Tobias M. Hedison, Katherine A. Hollywood, Reynard Spiess, Kathleen M. Cain, Sofia Diaz-Moreno, Nigel S. Scrutton, Morten Tovborg, Paul H. Walton, Derren J. Heyes, Anthony P. Green

**Affiliations:** † Manchester Institute of Biotechnology, The University of Manchester, 131 Princess Street, Manchester M1 7DN, U.K.; ‡ Department of Chemistry, University of York, Heslington, York YO10 5DD, U.K.; § Institute of Molecular Biotechnology, Graz University of Technology, Petersgasse 14, Graz 8010, Austria; ∥ Harwell Science and Innovation Campus, Diamond Light Source Ltd., Didcot, Oxfordshire OX11 0DE, U.K.; ⊥ Novozymes A/S, Krogshoejvej 36, Bagsvaerd 2880, Denmark

## Abstract

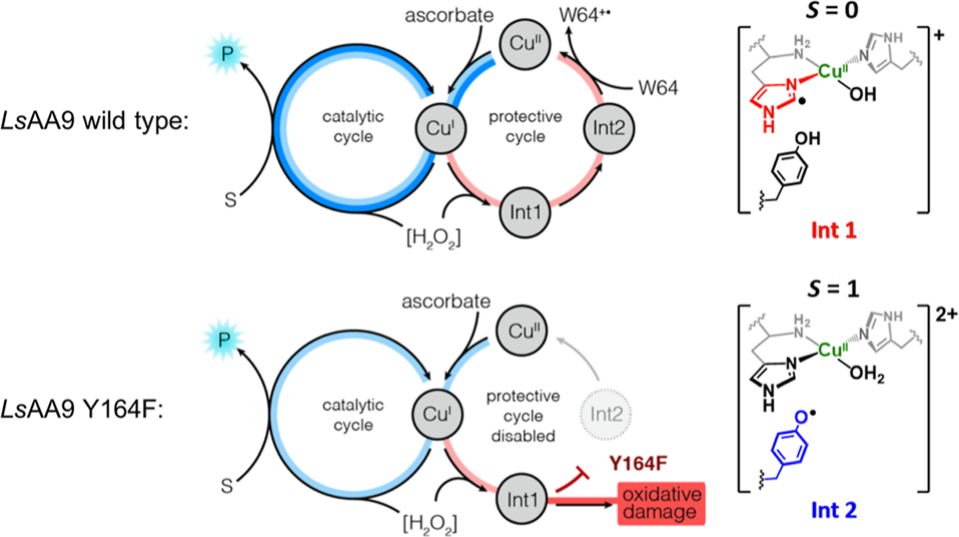

Oxygenase and peroxygenase enzymes generate intermediates at their active sites which bring about the controlled functionalization of inert C–H bonds in substrates, such as in the enzymatic conversion of methane to methanol. To be viable catalysts, however, these enzymes must also prevent oxidative damage to essential active site residues, which can occur during both coupled and uncoupled turnover. Herein, we use a combination of stopped-flow spectroscopy, targeted mutagenesis, TD-DFT calculations, high-energy resolution fluorescence detection X-ray absorption spectroscopy, and electron paramagnetic resonance spectroscopy to study two transient intermediates that together form a protective pathway built into the active sites of copper-dependent lytic polysaccharide monooxygenases (LPMOs). First, a transient high-valent species is generated at the copper histidine brace active site following treatment of the LPMO with either hydrogen peroxide or peroxyacids in the absence of substrate. This intermediate, which we propose to be a Cu^II^–(histidyl radical), then reacts with a nearby tyrosine residue in an intersystem-crossing reaction to give a ferromagnetically coupled (*S* = 1) Cu^II^–tyrosyl radical pair, thereby restoring the histidine brace active site to its resting state and allowing it to re-enter the catalytic cycle through reduction. This process gives the enzyme the capacity to minimize damage to the active site histidine residues “on the fly” to increase the total turnover number prior to enzyme deactivation, highlighting how oxidative enzymes are evolved to protect themselves from deleterious side reactions during uncoupled turnover.

## Introduction

**Figure 1:**
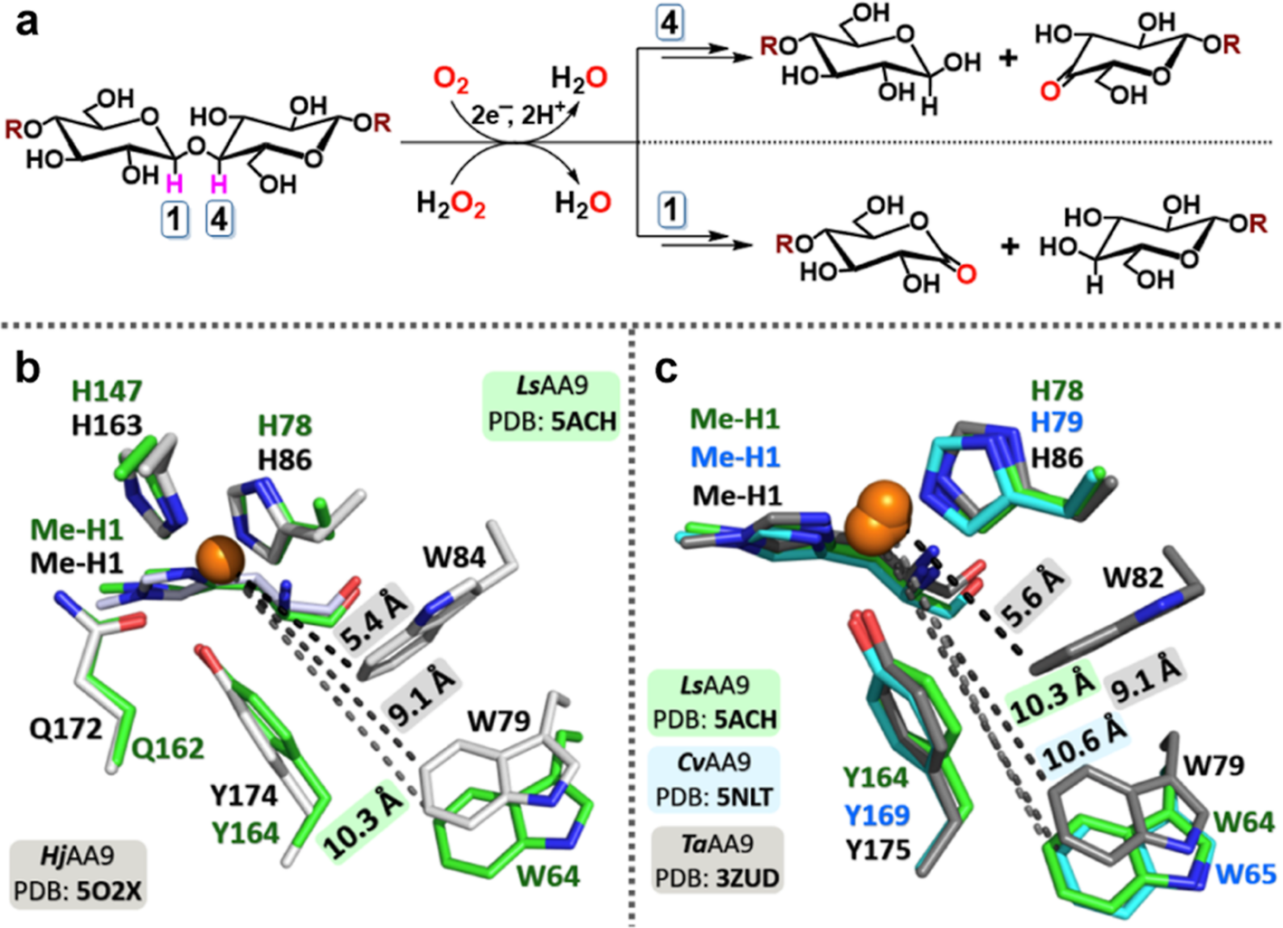
Structures and catalytic functions of LPMOs. (a) Chemical scheme depicting oxidative cleavage of oligosaccharides by LPMOs at C1 or C4. (b) Overlay of the *Ls*AA9 (PDB 5ACH) and *Hj*AA9 (PDB 5O2X) crystal structures. (c) Overlay of the *Ls*AA9 (PDB 5ACH), *Cv*AA9 (PDB 5NLT), and *Ta*AA9 (PDB 3ZUD) crystal structures. The copper ion is shown as an orange sphere, and key active site residues are shown as atom-colored sticks. The catalytic copper is chelated by two nitrogen atoms of an N-terminal histidine (or *N*-methyl histidine) and the N_τ_ nitrogen atom of a second histidine in a T-shaped configuration, termed the “histidine brace”.

Lytic polysaccharide monooxygenases (LPMOs) are enzymes secreted by aerobic organisms during the degradation of abundant biomass.^1,2^ LPMOs have become the focus of research effort not only due to their commercial potential in biorefineries^3^ and their roles as virulence factors^4^ in plant disease but also because these enzymes employ an oxidative mechanism for the cleavage of glycosidic bonds within polysaccharides (Figure 1a).^5,6^ Of particular interest in this regard is the recalcitrant nature of the polysaccharide substrate, which necessitates that LPMOs generate a potent oxidizing intermediate at their copper histidine brace (i.e., a copper ion chelated by the NH_2_ and π–N atom of a second histidine, Figure 1b) active site, in order to cleave selectively strong C–H bonds (dissociation enthalpy, ca. 95–100 kcal mol^–1^) of the glycosidic ring within the polysaccharide.^7^ The nature of this intermediate has not been experimentally determined but has been explored in multiple theoretical calculations, which propose it to be either a triplet state (*S* = 1) Cu^II^–oxyl, [Cu–O]^+^ or a singlet state (*S* = 0) Cu^III^–hydroxide, [Cu–OH]^2+^ (or possibly free hydroxyl radicals, following the enzyme-catalyzed homolytic fission of the O–O bond in H_2_O_2_).^8−12^ As with all oxygenase and peroxygenase enzymes, however, these intermediates can cause damage to the enzyme through oxidative cleavage of bonds on adjacent amino acids,^13,14^ an action which compromises enzyme efficiency.^15−17^ Thus, an outstanding question about the mode of action of LPMOs is how does the enzyme mitigate/prevent oxidative damage to preserve the integrity of key active site residues? In this regard, it is known that the type of substrate,^18,19^ the concentration of peroxide,^17^ the type of reducing agent,^20^ the degree of glycosylation^21^ and methylation of His1^22^ of LPMOs are all factors in the degree of substrate versus enzyme oxidation. Nonetheless, little is known about the fate of reactive intermediates at a mechanistic level, save for proposals from recent QM/MM calculations which suggest that the formation of a copper(II)–histidyl radical complex occurs during uncoupled turnover of AA9 LPMOs with peroxide.^23^

In preparation for our studies aimed at understanding the nature and reactivity of intermediates that are generated at the histidine brace active site, we were mindful of previous work on LPMOs, which had demonstrated that histidine, tryptophan, and tyrosine amino acids adjacent to the histidine brace in LPMOs can be oxidatively modified by the intermediate that is generated at the active site following the addition of peroxide or peroxy acids to Cu^I^–LPMOs in the absence of substrate.^15,21,24−26^ These previous works provided insight into the potential electronic coupling pathways between the histidine brace and redox-active residues that function as conduits for charge transfer through the protein by which intermediates at the active site can be extinguished.

However, these studies were unable to identify the mechanism by which any oxidative modification to the active site occurs. Moreover, rapid charge transfer associated with these pathways reduces the lifetimes of any species formed immediately after reactions with peroxides, thereby making their experimental observation challenging. Thus, in the expectation that the lifetime of any reactive species is a function of the distance between the histidine brace and any redox-active amino acids,^13^ we sought to take advantage of the fact that some structures of LPMOs exhibit the putative redox-active tryptophan at a distance of ca. 10.3 Å away from the active site, as opposed to the ca. 5.4 Å histidine brace–tryptophan distance in LPMOs used in previous studies (Figure 1b).^27^

Using this strategy, we describe here a previously unobserved and spectroscopically distinct intermediate, which forms and decays on a millisecond timescale following the addition of hydrogen peroxide or peroxyacids to Cu^I^-LPMOs. Electron paramagnetic resonance spectroscopy (EPR) and high energy resolution fluorescence detection X-ray absorption spectroscopy (HERFD–XAS) studies combined with TD-DFT calculations support an assignment of this intermediate as an open-shell singlet (*S* = 0) Cu^II^–(histidyl radical) complex, as recently proposed in calculations and in accordance with the known oxidative damage of histidine 1 during uncoupled turnover of LPMOs^15,21,23^ with H_2_O_2_. This species is extinguished by net hydrogen atom transfer from a nearby tyrosine to give an *S* = 1 ferromagnetically coupled Cu^II^–tyrosyl pair along with the restoration of the Cu^II^–LPMO resting state. This mechanism reveals the non-innocent properties of the histidine ligands of the histidine brace active site of copper oxygenases and in this case, their role in defending the enzyme against oxidative damage during uncoupled turnover.

## Results

### Transient Intermediates in LPMOs

**Figure 2:**
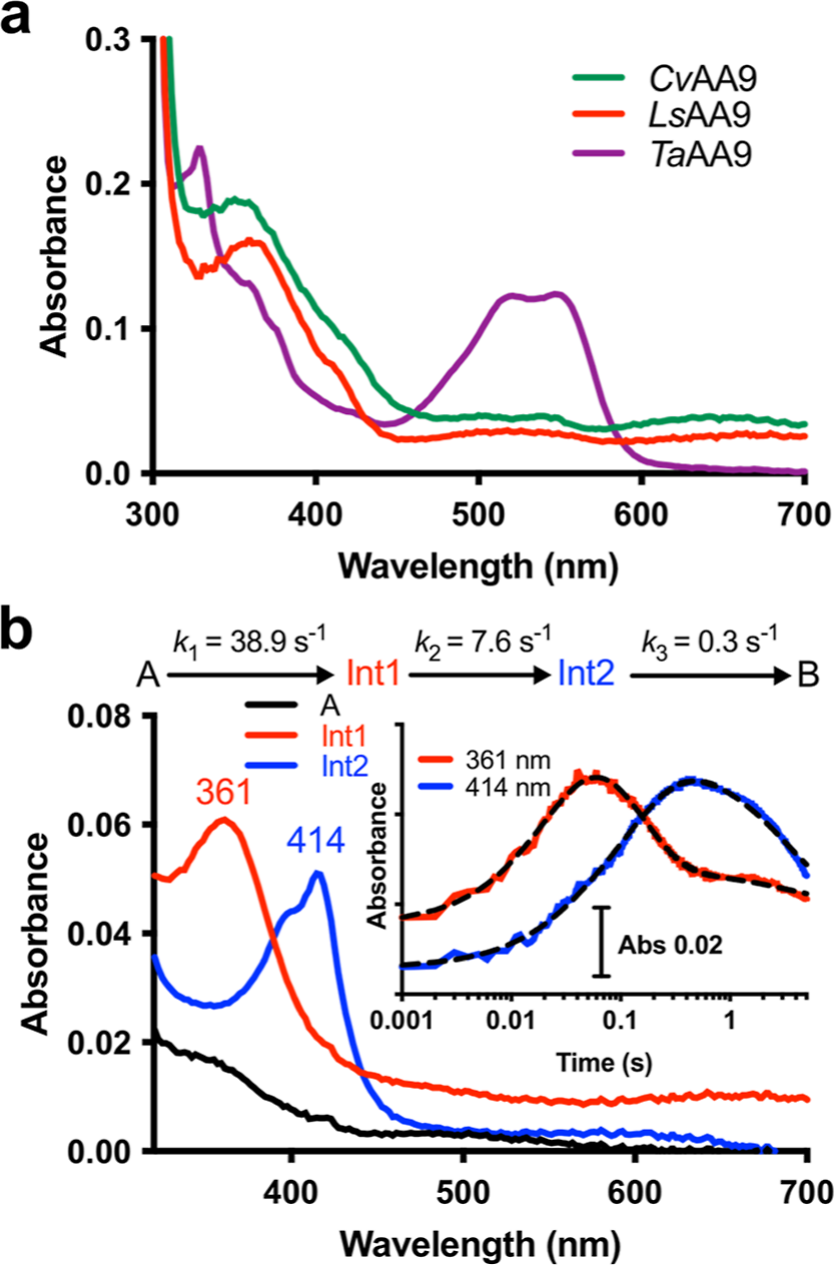
Stopped-flow analysis of intermediates formed upon LPMO oxidation. (a) Overlay of UV–vis spectra of transient intermediates generated upon oxidation of Cu^I^–*Ta*AA9, *Cv*AA9, and *Ls*AA9 with mCPBA (100 μM enzymes, 500 μM oxidant). The spectra shown are at 10 ms post-mixing. In *Ta*AA9, a tryptophanyl radical is formed with characteristic bands at 520 and 548 nm. In *Ls*AA9 and *Cv*AA9, a short-lived species (**Int1**) with weak absorption maxima at ca. 355–365 nm is observed. (b) UV–vis spectra of intermediates and their rates of formation upon mixing 50 μM reduced *Ls*AA9 with 50 μM peracetic acid (PAA). The multiwavelength stopped-flow data were fit to a sequential kinetic model to extract component spectra and global rate constants. A first intermediate (**Int1**) with a major absorbance feature at 361 nm rapidly accumulates and decays to a second species (**Int2**) with an absorbance at 414 nm consistent with a tyrosyl radical. The inset shows kinetic transients at 361 and 414 nm overlaid by fits from global analysis (black dashed lines).

In an effort to capture transient intermediates in LPMOs, we explored their reactivity with peroxide and peroxy acid oxidants. Stopped flow UV–vis spectroscopy studies were initially performed for the reaction of *meta*-chloro-perbenzoic acid (*m*-CPBA), with three different Cu^I^–LPMOs. This oxidant has previously been used to isolate the reactive Compound I state in P450 enzymes.^28^ One of the LPMOs (*Ta*AA9) has a tryptophan residue close to the active site as described above, whereas two others (*Ls*AA9 and *Cv*AA9) have structures with a more distant tryptophan (Figure 1c).^29,30^ In the former, in accordance with previous studies,^24^ we observed the appearance of two optical intermediates in stopped-flow measurements that lie on divergent pathways, viz. a tryptophanyl radical with characteristic bands at 520 and 548 nm with associated features at 329 and 355 nm (Figure 2a), and a longer-lived tyrosyl radical with characteristic bands at ca. 420 nm (Figure S1).^25^ These species are formed with ∼65 and ∼26% conversions, respectively, based on known extinction coefficients of neutral tryptophanyl and tyrosyl radicals (1900 and 2600 M^–1^ cm^–1^ respectively).^24^ The latter, but not the former, of these species was previously observed in spectroscopic studies of *Ta*(AA9), where it was suggested that the tyrosyl radical species could be part of the catalytic cycle of LPMOs (see below ref (25)), although more recent studies suggest that it is unlikely that such a species is catalytically active for substrate oxidation.^24,31^

In contrast, in the cases of *Cv*AA9 (Figure S2) and *Ls*AA9 (Figure S3) no significant oxidation of tryptophan was observed. Instead, oxidation of a tyrosine, as evidenced by absorption with λ_max_ = 414 nm (hereafter **Int2**) was preceded by the appearance of a short-lived species with λ_max_ = ∼360 nm (hereafter **Int1**) and a further weak absorption in the 600–800 nm region of the spectrum. These kinetic data are in accord with the expected lower degree of electronic coupling of the tryptophan residue with the active site in *Ls*AA9 and *Cv*AA9 as compared to *Ta*AA9 and would appear to support the hypothesis that the lifetime of an intermediate, **Int1**, is controlled by coupling to adjacent redox-active amino acids. It should be noted that although similar intermediates are observed in both the *Ls*AA9 and *Cv*AA9 enzymes, there are some differences in the intensity and lifetime of each of these species, showing that various factors contribute to their stabilities. In this vein, during the preparation of this manuscript, Hedison et al. reported stopped-flow data which showed the rapid formation of both tyrosine and tryptophan radicals in *Nc*AA9C, where the closest tryptophan lies ca. 10 Å from the active site.^26^ In this case, the formation of the tryptophan radical is uncoupled from that of the tyrosine revealing the presence of separate but kinetically similar charge transfer pathways in *Nc*AA9C. For *Ls*AA9 and *Cv*AA9, despite the similar tryptophan to active site distances (∼10 to 11 Å) it is evident that the charge transfer pathway to a tryptophan radical is slower than that to the tyrosine radical (**Int2**), for reasons which are not clear but likely reflect differences in the electronic microenvironments of nearby tryptophans in *Nc*AA9C compared with those in *Ls*AA9 and *Cv*AA9, e.g., closest distance to adjacent phenylalanine (4.5 Å in *Nc*AA9C, 4.9 Å in *Ls*AA9).

We next developed an expression system for *Ls*AA9 in *Escherichia coli* (Figure S4), which gave access to significant amounts of active protein needed for further intermediate characterization, and facilitated subsequent site-directed mutagenesis studies (vide infra). Similar rates of formation/decay of **Int1** and **Int2** are observed in *Ls*AA9 produced in fungal and bacterial expression systems (Figure S5). Global modeling of the observed rates confirmed a sequential **A** → **Int1** → **Int2** → **B** model, where **A** is Cu^I^–LPMO and **B** is the Cu^II^ resting state of the enzyme (assigned from the lack of any distinct spectral features in its visible spectrum). The sequential nature of this reaction pathway, which likely involves, or is triggered by, O–O bond cleavage of the oxidant (see Discussion), is evident when comparing the kinetic transients at 361 and 414 nm (Figure 2b inset). Using a molar absorption coefficient (*Y*·ε_420 nm_) of 2600 M^–1^ cm^–1^, we estimate that ∼35–40% of reduced *Ls*AA9 and ∼55% of reduced *Cv*AA9 are converted to **Int2** (via **Int1**), through this pathway.

Within error, the rates of formation and the reaction of **Int1** and **Int2** and their spectral features were found to be independent of pH (6 to 8, Figure S6), buffer (phosphate, MES, Figures S7 and S8) and initial reductant (Figure S9). Furthermore, no optical intermediates were observed when the Cu^II^ form of the enzyme was used, demonstrating that the observed stopped-flow spectra arise from the oxidation of the Cu^I^-state of the LPMO (Figure S10). We then examined the role of H_2_O_2_, PAA, and *m*-CPBA as oxidants, which all gave similar patterns of behavior and identical spectra for **Int1** and **Int2**. The rate of conversion from **Int1** to **Int2** was found to be independent of H_2_O_2_ concentration (12.8 ± 0.1 and 11.5 ± 0.02 s^–1^ with 10 equiv and 50 equiv of H_2_O_2_, respectively, Figures S8 and S11) and oxidant (PAA and *m*-CPBA, 7.6 ± 0.01 s^–1^ and 9.0 ± 0.01 s^–1^, Figures 2b, S12 and S13). The rate of **Int2** decay was also independent of oxidant concentration/identity (Figures S8, S11–S14). The only significant differences between oxidants were seen in the rates of formation of **Int1** (Figure S15), which are sensitive to the nature of the oxidant and the oxidant concentration. Notably, **Int1** did not accumulate when a stoichiometric amount of H_2_O_2_ was used (Figure S16), as its rate of formation is substantially slower than its rate of decay under these conditions, but was observed at higher equivalents. In contrast, **Int1** formation was observed with stoichiometric quantities of the more reactive peroxy-acid oxidants, *m*-CPBA or PAA. For instance, rates of formation of 38.1 ± 0.1 and 38.9 ± 0.1 s^–1^ at 277 K were observed with one equivalent of *m*-CPBA and PAA respectively (Figures S12 and S13), and 14.8 ± 0.2 and 99.1 ± 0.3 s^–1^ at 277 K were observed with 10 equiv and 50 equiv of H_2_O_2_, respectively (Figures S8 and S11). The rates of **Int1** and **Int2** formation were also measured in the D_2_O buffer to determine whether any solvent kinetic isotope effects (SIEs) were associated with either step. An SIE of 1.6 ± 0.2 was observed on the rate of formation of **Int1**, which is consistent with the value reported in pathways involving heterolytic cleavage of the oxidant O–O bond (Τable S3).^24^

### Intermediates 1 and 2 Do Not Oxidize Oligosaccharide Substrates

**Figure 3:**
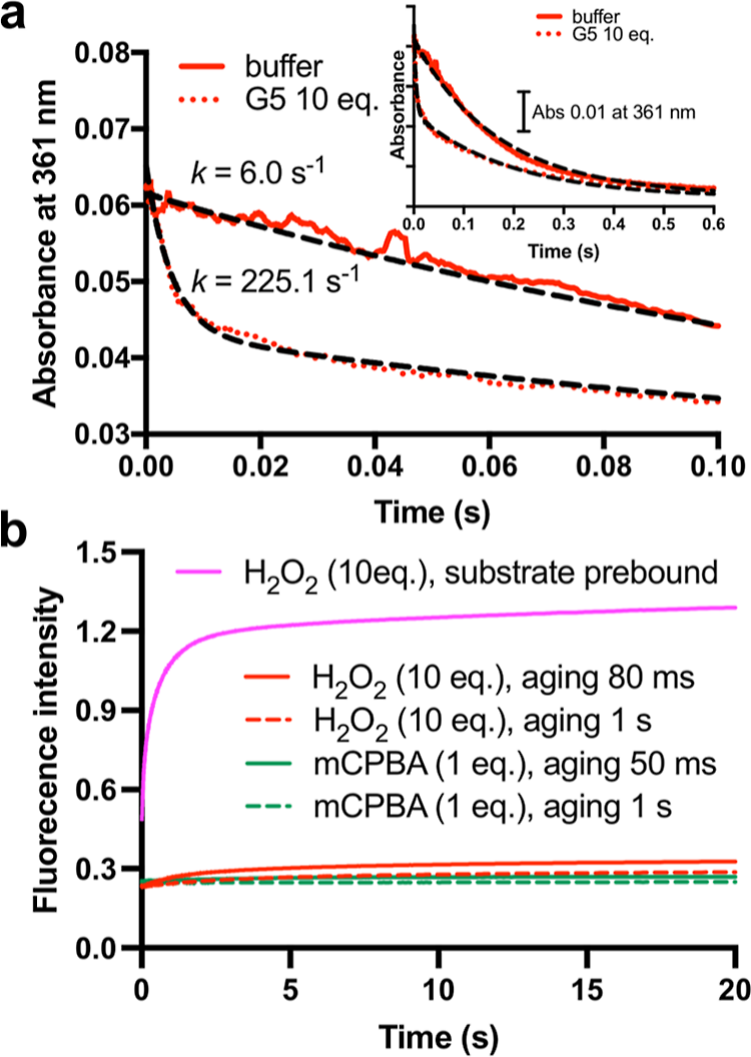
Exploring **Int1** and **Int2** reactivity with oligosaccharide substrates. (a) Double mixing stopped-flow experiments showing absorbance changes at 361 nm after 50 μM reduced wild-type *Ls*AA9 was first mixed with 50 μM PAA, aged for 50 ms to generate **Int1**, prior to a second mix with either buffer (red) or 500 μM G5 substrate (red dotted). Black dashed lines show the fits of the data to a double exponential. (b) Double mixing stopped-flow kinetics showing fluorescence changes upon mixing **Int1** or **Int2** with FRET-G4 (see Figure S29). Reduced Cu^I^–*Ls*AA9 (50 μM) was first mixed with *m*-CPBA (50 μM) or H_2_O_2_ (500 μM), allowed to age for either 50 ms, 80 ms or 1 s, then mixed with the FRET-G4 (100 μM). All raw data are shown in the Supporting Information.

To explore the reactivity of **Int1** with oligosaccharide substrates, we next carried out stopped-flow measurements in the presence of cellopentaose (G5), a known substrate for *Ls*AA9.^15^ No optical intermediates were observed when oxidants were added to a mixture of G5 and Cu^I^–*Ls*AA9 (Figure S17), showing that **Int1** and **Int2** are not formed under these conditions, or that their rate of decay is much faster than their rate of formation in the presence of substrate. We therefore turned to double mixing stopped-flow experiments, where Cu^I^–*Ls*AA9 is mixed first with stoichiometric amounts of oxidant, allowed to age for 50 ms to generate a maximal concentration of **Int1**, and then mixed with G5. Under these conditions, **Int1** decayed ca. 40-fold faster in the presence of G5 than in the absence of substrate (i.e., with 500 μM G5, rate = 225.1 ± 3.2 vs 6.0 ± 0.1 s^–1^ with buffer only) (Figures 3a and S18). **Int2** decay (bi-exponential) was also accelerated (i.e., with 500 μM G5, *k* = 38.4 ± 0.8 vs 0.20 ± 0.001 s^–1^ with buffer only, Figure S19). These rates of decay were dependent on substrate concentration (see PDA data in Figures S20–S26). However, the presence of the cellobiose (G2 product) in the double mixing experiments had no effect on the rate of decay of either intermediate (Figures S27 and S28).

Despite the fact that the rates of decay of **Int1** and **Int2** are accelerated by substrate binding, it cannot be inferred from these data that either intermediate is part of the oxygenase’s productive catalytic cycle. As such, we undertook two experiments to determine the role, if any, of **Int1** and **Int2** in the productive catalytic cycle of *Ls*AA9. In both experiments, the double mix experiment described above was repeated using both H_2_O_2_ and *m*-CPBA as oxidants but mixed at the second stage with either: (i) a known cellotetraose substrate for *Ls*AA9 which fluoresces upon cleavage (FRET-G4, Figure S29), or (ii) analyzing the double-quench samples described above (with variable double-mixing times of 50, 300 and 1000 ms after the initial mix) for any cleavage products and their amounts as a function of **Int1** or **Int2** concentration (Figure S30). In both cases, no evidence of intermediate-dependent substrate cleavage was found, showing that neither **Int1** nor **Int2** is likely to be part of the productive catalytic cycle of *Ls*AA9. In contrast, control experiments where reactions are initiated by the addition of H_2_O_2_ to a mixture of substrate and enzyme led to product formation as anticipated (Figure 3b). Given that neither **Int1** nor **Int2** appears able to oxidize oligosaccharide substrates, it is interesting that these intermediates decay more rapidly in the presence of oligosaccharides. Substrate binding in *Ls*AA9 leads to changes in copper redox potential and alters the environments and relative positioning of important active site residues. For example, substrates are known to interact with the face of His1.^30^ Such changes will likely result in changes in p*K*_a_ and ionization energies of redox-active residues proximal to the active site, and as such will influence the lifetimes of oxidized intermediates.

### Intermediate 2 is a Ferromagnetically Coupled (*S* = 1) Cu^II^–Tyrosyl Complex

**Figure 4:**
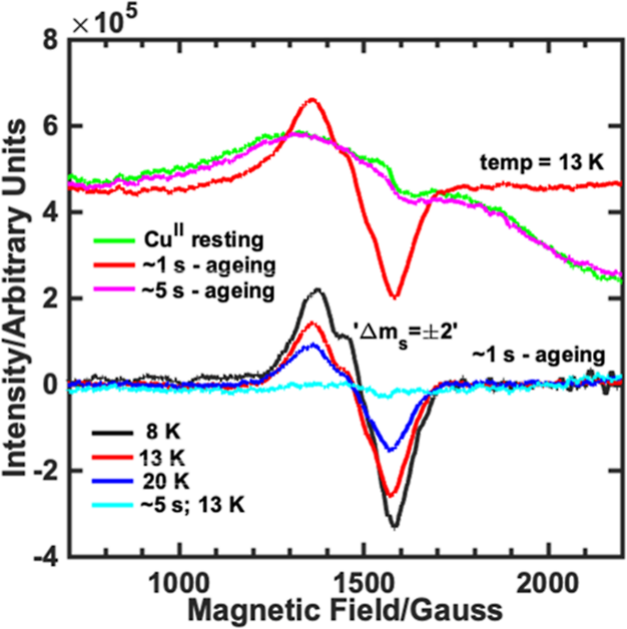
CW-EPR spectra of selected samples in the low-field region show the half-field “Δ*m*_S_ = ± 2” transitions. Temperature-dependent spectra are shown (bottom) with resting-state EPR signals subtracted manually. All raw data are shown in the Supporting Information.

The relatively long lifetime (1–5 s) of **Int2** allowed us to trap this species using standard freeze-quench methods (see Methods). Thus, using an **Int2** sample quenched after ∼1 s, we investigated its temperature- and power-dependent CW-EPR spectra and compared these to two control samples (Cu^II^–*Ls*AA9 and a sample quenched after 5 s). Spectra of **Int2** collected over the temperature range of 8–20 K showed the presence of three different EPR active species for the ∼1 s sample; (i) signals from a Cu^II^ ion (*S* = 1/2), with spin Hamiltonian parameters that match that of the *Ls*AA9 resting state (Figure S31—top panel),^21^ (ii) a sharp signal from an organic-like radical (*g* ∼ 2.00) without noticeable magnetic-splitting (*S* = 1/2) and with low intensity (ca. < 1% of the integrated Cu^II^ intensity; Figure S31—top and bottom panels with black asterisk mark) and (iii) half-field, forbidden transitions, “Δ*m*_S_ = ±2”’ at 1500 G (Figure 4) and allowed “Δ*m*_S_ = ±1” transitions (Figures S33 and S34).^32^ The observation of the two *S* = 1/2 signals (resting-state Cu^II^ signal and sharp signal at *g* ∼ 2.00) show that the trapped sample contains products that likely arise from the direct homolytic fission of the peroxide bond to give Cu^II^–OH and an organic-based radical. From modeling of the low-temperature EPR data, this constitutes ca 62% of the whole sample, in good agreement with stopped-flow data, and shows that this is the dominant pathway, similar to a previous report.^24^ In addition to the products from the homolytic pathway, the half-field transition and its temperature- and power-dependent behavior reveal the formation of a separate triplet state (*S* = 1) species (calculated dipolar coupling of **T** = [+3417 + 917 – 4334]), and *J* ∼ −100 MHz (note here that the calculated value of exchange coupling^32^ is not determined to a high degree of precision, Figures S35–S37). A spin–spin distance can be estimated for two separate cases: when the dipolar interaction tensor is assumed to be approximately axial, **T** ∼ [−*T*, −*T*, +2*T*] giving *T* = 2167 MHz (Cu···O = 2.88 Å), and when the tensor is assumed to be rhombic, **T** ∼ [−*T*, 0, +*T*] giving *T* = 4344 MHz (Cu···O = 2.29 Å). In combination with the fact that **Int2** has clear absorption features that match those of a tyrosyl radical, such a coupling can only realistically arise from unpaired electrons on the Cu^II^ ion and a tyrosyl radical that lies adjacent to the Cu in the active site of LPMOs (Cu···O = 2.6 Å, Figure 1). The triplet EPR signals are not observed in Cu^II^–*Ls*AA9 nor in the sample freeze-quenched after ∼5 s (Figures 4 and S32, S33 and S36), indicating the direct correlation between the population of tyrosyl radical observed in stopped-flow measurement and formation of the triplet state (*S* = 1).

### Mutagenesis of Active Site Residues Perturbs Intermediate Formation and Decay

**Figure 5:**
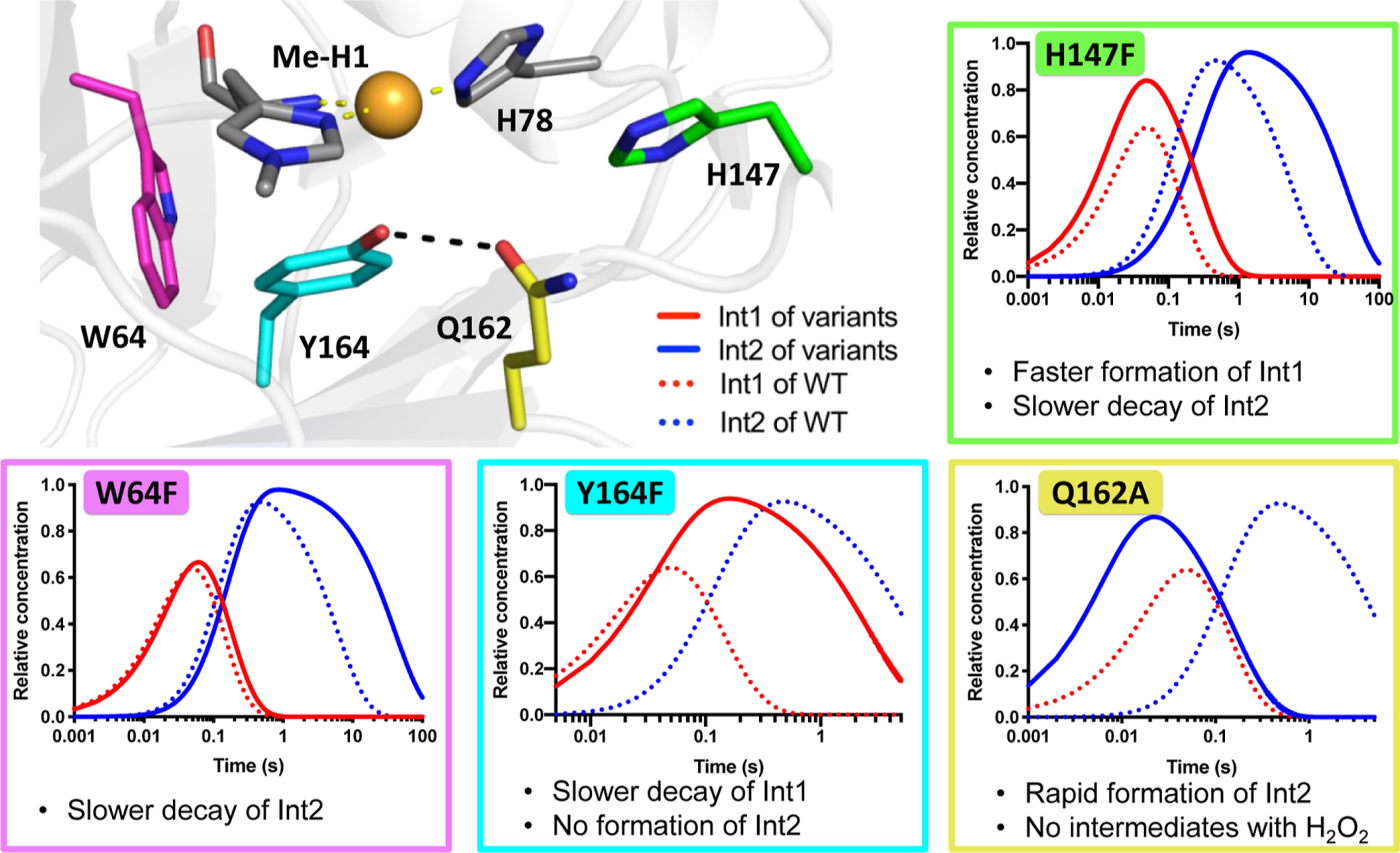
Impact of point mutations on intermediate formation and decay. Crystal structure of *Ls*AA9 active site, showing copper (orange), the histidine brace (grey atom colored sticks), and key active site residues selected for mutagenesis: H147 (green), W64 (pink), Y164 (cyan), and Q162 (yellow). For each of the mutants H147F, W64F, Y164F, and Q162A, concentration profiles of **Int1** (red) and **Int2** (blue) derived from global fitting software are shown as a function of time (solid line) in comparison to the wild-type enzyme (dotted line). All raw data can be found in Supporting Information.

To confirm the identity of the Tyr radical in **Int2**, we prepared Y164F and W64F variants of *Ls*AA9 (Figures 5 and S40). The active site Tyr164 and the neighboring Trp64 (closest aromatic ring to aromatic ring distance of 5.3 Å) are thought to form part of a charge transfer pathway from the active site to the protein surface.^21^ Stopped-flow analysis of Y164F showed the formation of a single intermediate species (Figure S41), with very similar absorbance features to **Int1**. This intermediate is not converted into **Int2** but instead decays to a nondescript spectrum at a rate similar to that of **Int2** decay in the wild-type enzyme. In contrast to the wild-type enzyme, temperature- and power-dependent CW-EPR analysis (5–20 K) on a freeze-quench trapped sample (0.5–1 s after reaction, 60–80% **Int1** relative concentration) revealed no half-field signals associated with a triplet species, showing that **Int2** is not formed in the reaction and that **Int1** is likely an EPR silent *S* = 0 spin singlet, although whether this is in an open or closed-shell form cannot be determined from the EPR data alone (Figures S36 and S37, see below for further discussion). Oxidation of the reduced W64F variant leads to the formation of **Int1** and **Int2** (Figure S42) at similar rates to wild-type *Ls*AA9. However, the rate of decay of **Int2** in W64F is > 10-fold slower than in the wild-type enzyme, suggesting that the tyrosyl radical of **Int2** is reduced by rapid “hole-hopping” from Trp64. This behavior is consistent with the site of tyrosyl radical identified in EPR studies of **Int2** being Y164, affirming the identity of **Int2** as a ferromagnetically coupled Cu^II^···Y164^•^ pair.

The roles of two additional conserved residues, His147 and Gln162, in intermediate formation and decay were also explored. His147 lies adjacent to the histidine brace and participates in π-stacking interactions with His78. With His147 replaced by Phe, **Int1** and **Int2** were formed as in the wild-type enzyme, albeit at slightly different rates (*k*_1_ = 61.9 ± 0.2 s^–1^ and *k*_2_ = 3.6 ± 0.01 s^–1^), showing that His147 does not contribute significantly to the electronic features of **Int1** and **Int2** (Figure S43). Interestingly no intermediates were observed upon oxidation of H147A and H147Q variants (Figures S44 and S45), suggesting that π-stacking interactions with His78 may be important for **Int1** formation and/or stability, plausibly by restricting accessible conformations of His78. We next explored the role played by Gln162. This residue is conserved across all AA9 LPMOs and is well-positioned to interact with H_2_O_2_ (or O_2_-derived species) bound at the vacant equatorial coordination site. Previous studies have shown that mutation of this conserved glutamine abolishes catalytic activity,^33^ and QM/MM calculations invoke an important role for the glutamine in orientating a peroxide within the active site.^34^ The relative orientation of frontier orbitals between the Cu(I) and H_2_O_2_ was also described in a recent X-ray absorption spectroscopy study of AA9 LPMOs.^35^ Stopped-flow measurements with a Q162A variant revealed that no intermediates are accumulated upon oxidation with H_2_O_2_ (Figure S46), consistent with Gln162 playing a role in the formation of a Cu^I^···HO–OH species with the peroxide bound at the active site. In contrast, using *m*-CPBA as the oxidant, where the O–O bond strength is expected to be weaker than in H_2_O_2_,^36^ leading to rapid accumulation of **Int2** (Figure S47) with no observable accumulation of **Int1**. Although not observed directly, we presume **Int2** formation still proceeds via **Int1**, but that the Q162A mutation decreases **Int1** stability, preventing its accumulation on the timescale of the experiment before the formation of **Int2**.

### Spectroscopic Properties of Intermediate 1 are Commensurate with an Open-Shell Singlet (*S* = 0) Cu^II^–(Histidyl Radical)

**Figure 6:**
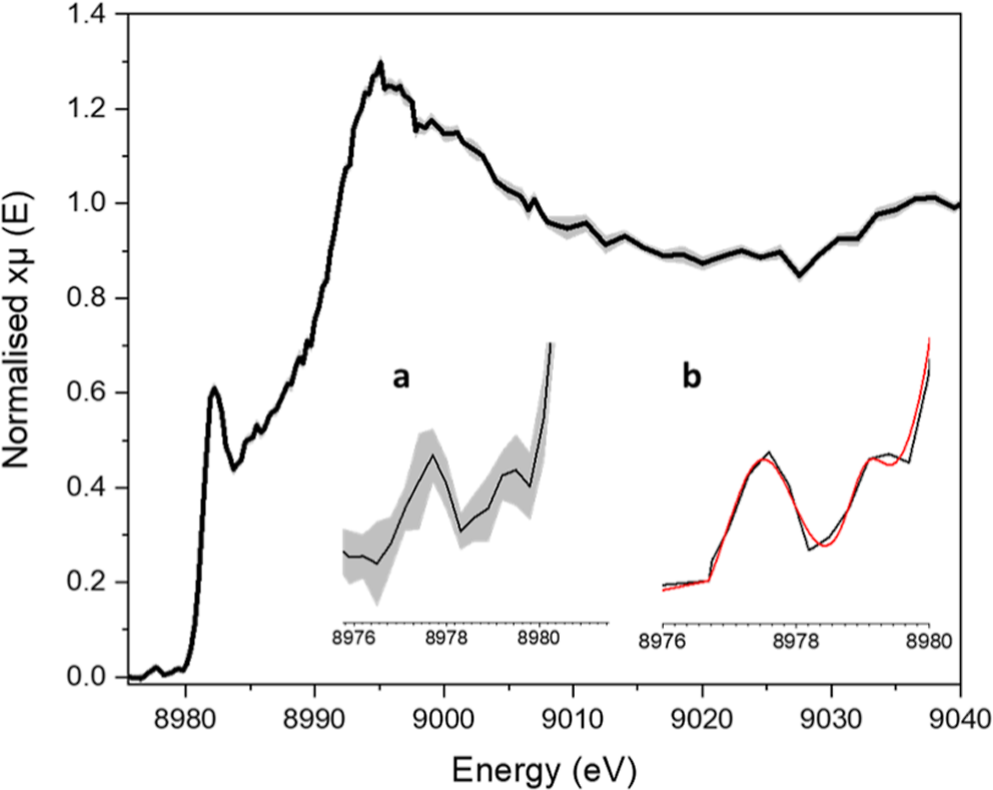
Copper K-edge HERFD–XAS spectrum (collected at 10 K) of **Int1** trapped at 100 ms and annealed to 150 K, 0.3 eV resolution in pre-edge edge regions, plotted with the standard error (grey). Inset (a) is a blow-up of the pre-edge region. Inset (b) shows experimental data (black) and the sum of individual Gaussians (red) from TD-DFT calculations (B3LYP, 17% HF exchange).

The characterization of **Int2** as a Cu^II^–tyrosyl radical pair is informative as to the nature of **Int1**, not least in the fact that **Int1** must exist at an oxidation state level which is one higher than a formal Cu^II^ state. To determine its electronic structure, we took advantage of the extended lifetime of **Int1** in the Y164F variant to facilitate its trapping using rapid freeze quench methods and characterization using HERFD–XAS (Figures S48–S50). Copper K-edge HERFD–XAS spectra (collected at 10 K to avoid significant photoreduction of the sample, Figure S48) of samples trapped at 100 ms, and subsequently annealed to 150 K, revealed an edge position at 8985.8(3) eV, consistent with a sample that is principally in the Cu^II^ oxidation state.^21^ An intense rising-edge feature at 8981.9(3) eV is also observed, likely to be either a Cu^II^ shakedown transition often observed in the K-edge XANES spectra of Cu^II^ species,^37^ or a 1s–4p transition that is observed in the equivalent Cu^I^ form of the enzyme at the same position. The latter could arise from partial photoreduction of the sample or any unreacted Cu^I^ form of the enzyme remaining in the sample.^21^ Notably, two weak pre-edge features at 8977.5(3) and 8979.2(3) eV were also observed, each with signal-to-noise ratios >10 (Figure 6a).

The feature at 8977.5(3) eV is commensurate with a dipole-disallowed, quadrupole-allowed 1s–3d(*x*^2^–*y*^2^) transition, commonplace^38,39^ in Cu^II^ enzymes and complexes. The transition is also in accord with previous Cu K-edge XAS studies on wild-type Cu^II^–*Ls*AA9, which exhibit the same transition at 8977.4 eV.^21^ The pre-edge feature at 8979.2(3) eV, on the other hand, is not a common absorption feature in Cu^II^ complexes, and—in the Cu^II^ oxidation state—can only realistically arise from a dipole-forbidden Cu(1s) to a metal/ligand LUMO/SOMO transition. Such requirements are fulfilled by a radical based on a ligand that is directly coordinated with the Cu^II^, such that the ligand-based SOMO has a metal character. Indeed, a similar weak pre-edge transition has been observed previously for oxidized forms of *Ls*AA9 at 8982.8 eV, which in this earlier case was assigned to a Cu(1s) to ligand charge transfer transition in an inactive Cu^II^–tyrosyl species (that is only formed upon prolonged exposure to hydrogen peroxide).^21^ However, we can rule out the source of the absorption at 8979.2(3) eV in the **Int1** sample, as arising from the previously reported Cu^II^–tyrosyl species in *Ls*AA9, since the latter occurs at 8982.8 eV, and the tyrosine adjacent to the copper center has been replaced by phenylalanine in the Y164F variant used in this study.

In making a putative assignment of the pre-edge transitions in **Int1**, we turn to previous work on the UV–vis spectra of transient imidazolyl radicals, Cu^II^–(imidazolyl radicals) and Cu^II^–(histidyl radicals), all of which exhibit semi-intense absorptions at ca. 360 nm (ε > 2000 M^–1^ cm^–1^),^40,41^ and also that of an isolated imidazolyl radical with an intense band at 365 nm (ε ∼ 2000 M^–1^ cm^–1^).^42^ The analogy between these absorptions and that in **Int1** directs us towards the possibility that **Int1** is, in fact, a Cu^II^–(histidyl radical) complex. Indeed, given the need from the HERFD–XAS data for the Cu-based intermediate to have some radical character from the ligands, this species offers itself as one of only two possibilities, where the other is one in which the radical “hole” exists on the exogenous ligand coordinated to the Cu^II^ such as a Cu^II^–oxyl species.^31^ It is not possible to separate the two possibilities on the basis of the combined UV–vis and HERFD–XAS data, although the lack of reactivity of **Int1** (see above) with substrate strongly indicates that **Int1** is not a Cu^II^–oxyl.

TD-DFT calculations for the pre-edge region of the XAS of **Int1**(43) were performed on an optimized structure of the active site of *Ls*AA9 for a Cu^II^–histidyl (broken symmetry, singlet surface), where the hydrogen atom of the C2 carbon of His1 had been removed to give the His1-radical (Figure S51). The C2 site for radical formation was chosen in the knowledge that C2–H atom abstraction to give histidyl radicals has been observed for Cu^I^-superoxide dismutases treated with peroxide,^44^ that oxo-histidine is the principal product from the reaction of AA10 LPMOs with H_2_O_2_ in the absence of substrate,^15^ and that recent DFT calculations on *Ls*AA9 have demonstrated radical formation at this site following formation of a transient Cu^II^–oxyl.^23^

As expected, the calculations afforded two pre-edge transitions in the XAS spectrum: 1s–3d(*x*^2^–*y*^2^) and 1s-metal/histidyl transitions. The energy separation of the two varied between 0.50 and 4.94 eV dependent on the degree of Hartree–Fock (HF) exchange which was included in the hybrid functional. At 30% HF exchange, the calculated energy separation was 4.9 eV, as compared to 4.3 eV which was previously calculated for the stable Cu^II^–tyrosyl radical species that forms upon long-term exposure of LsAA9 to H_2_O_2_. At 17% HF exchange, the pre-edge peaks have a separation of 1.7 eV, corresponding to that from experiment and, moreover, relative intensities which match those seen experimentally (Figure 6b), showing that this level of HF exchange in the functional is a good model for the experimental spectrum. (We note here that the fully annealed sample displays a much less intense peak associated with the 1s–3d(*x*^2^–*y*^2^) transition and thus any signal arising from any residual Cu^II^–*Ls*AA9 in the sample does not interfere with the relative intensities of the two pre-edge peaks seen in **Int1**.) Thus, the combined data from XAS, UV–vis spectroscopy, and TD-DFT, along with the lack of reactivity of **Int1** with a substrate, are consistent with **Int1** being a transient Cu^II^–(histidyl radical).

### **Int1** and **Int2** are Part of a Radical Dissipation Pathway

**Figure 7:**
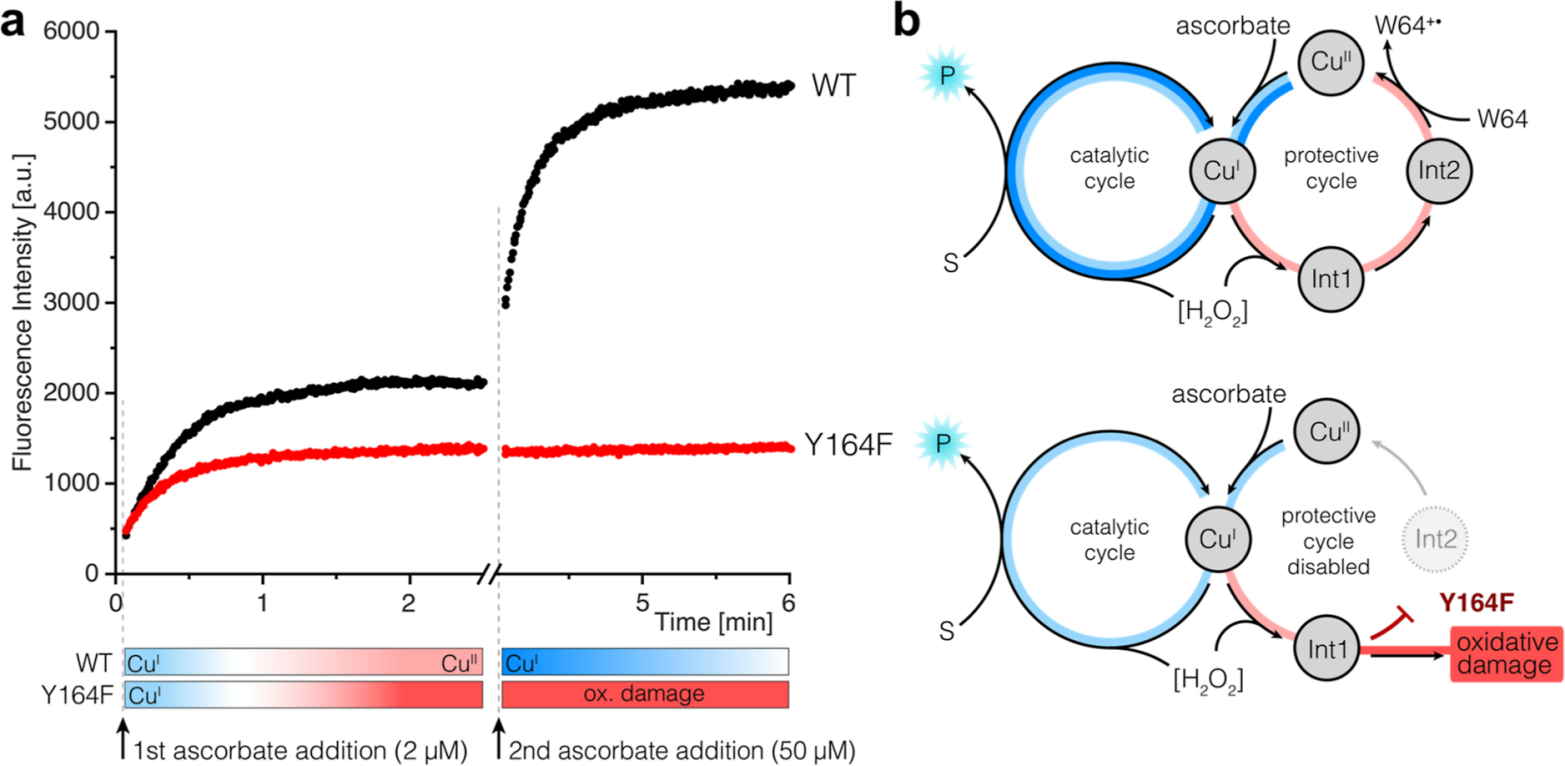
Proposed protective mechanism operating in *Ls*AA9. (a) Steady-state assays using a fluorogenic substrate with *Ls*AA9 WT (black) and Y164F (red). Reaction conditions: enzyme (1 μM), substrate (20 μM), H_2_O_2_ (150 μM), ascorbate (2 μM) in KPi buffer (50 mM, pH 6). Addition of ascorbate (50 μM) after 3 min restores the catalytic activity of *Ls*AA9 WT but not the Y164F variant. (b) In *Ls*AA9 WT, **Int1** and **Int2** form part of a protective cycle that restores the copper-histidine brace to its resting state, which can then re-enter the catalytic cycle through reduction. This protective pathway is disabled in the Y164F variant.

To explore the functional role of the **Int1**/**Int2** charge transfer pathway, we next carried out steady-state assays with wild-type *Ls*AA9 using FRET-G4. For comparison, assays were also performed with the Y164F variant where the charge transfer pathway has been disabled. Assays were performed in the presence of H_2_O_2_ and were initiated by the addition of ascorbate as a reductant. The wild-type enzyme and the Y164F variant show similar catalytic behavior, with the rapid initial formation of the anticipated fluorescent product followed by apparent deactivation (Figure 7a). We note that the initial reaction velocities with WT and Y164F are very similar, suggesting that the Y164F substitution does not substantially alter substrate binding or Cu reactivity. However, the origin of the observed deactivation is different in the two variants. In the wild-type enzyme, activity can be restored by the addition of reductant (or through consecutive additions of reductant, Figures 7a and S52), ultimately resulting in fluorescence intensity anticipated following the complete conversion of substrate to product. In contrast, the activity of the Y164F variant cannot be restored with reductant, and increases in reaction conversion can only be achieved by the addition of fresh enzyme. Moreover, we have subjected both enzymes to multiple cycles of uncoupled turnovers prior to catalysis and shown that significant levels of activity are retained in the wild-type, but not in the Y164F enzyme (Figure S53). Consistent with these activity measurements, intact protein mass spectrometry analysis shows that oxidative damage is more extensive in Y164F compared with the wild-type enzyme (Figure S54). Taken together, from these experiments, we can infer that the **Int1**/**Int2** charge transfer pathway protects the active site from oxidative damage during uncoupled turnover by restoring the copper-histidine brace to its resting state, which can then re-enter the catalytic cycle through reduction (Figure 7b).

## Discussion

LPMOs generate high-valent intermediates at their copper histidine brace active sites. In coupled turnover, the high reactivity of these intermediates is crucial in effecting hydrogen-atom-transfer (HAT) from the polysaccharide substrate. On the other hand, the potential to generate such intermediates during uncoupled turnover remains, despite the fact that their oxidizing power cannot be directed towards the substrate. In such cases, in common with other oxygenase and peroxygenase enzymes, LPMOs contain mechanisms by which the deleterious reaction of the intermediate with the protein is reduced. The details of these mechanisms in LPMOs are beginning to be revealed, consisting principally of: (1) substrate-dependent oxidant-activation mechanisms in LPMOs and (2) charge-transfer pathways made up of redox-active amino acids that extend from the histidine brace in AA9 LPMOs to the protein surface, which can “extinguish” a reactive intermediate inadvertently generated at the active site.^12,21,45^ In this context, our studies herein describe the formation of a previously uncharacterized intermediate (**Int1**) formed during the reaction of a Cu^I^–LPMO with peroxides in the absence of substrate. Our combined data best support the assignment of **Int1** as a Cu^II^–(histidyl) radical. We propose His1 as the most likely site for radical formation based on recent DFT calculations on the same enzyme and the close proximity of this residue to Tyr164.^23^ In this regard, it is interesting that studies on fungal LPMOs show that methylation of His1 protects LPMOs from oxidative damage during catalysis.^22^ Our current studies show that **Int1** is not ostensibly competent for oxidation of substrate, but rather that it lies on a separate uncoupled pathway to the catalytic cycle, forming the first part of the mechanism in LPMOs for extinguishing potentially damaging intermediates.

**Int1** results from the direct reaction of Cu^I^–LPMO with peroxide or peroxy acids in what can be described as a “net” heterolytic cleavage of the peroxide (Figure 6). From our current data, we cannot gain mechanistic insight into the reaction mechanism which precedes the formation of **Int1**. Notwithstanding the various potential pathways, however, recent calculations by Torbjörnsson et al., indicate that **Int1** forms from direct HAT from a Cu^II^–oxyl species. Such a mechanism is consistent with our experimental observations. Moreover, the consistency between different peroxides and peroxy-acids in the generation of **Int1** is commensurate with this mechanism. Whatever the initial mechanism, it is evident that **Int1** is readily formed in the active sites of *Ls*AA9 and *Cv*AA9 LPMOs, directing us toward the suggestion that it is part of a bespoke pathway for diffusing reactive intermediates in these enzymes and perhaps the wider LPMO family.

**Figure 8:**
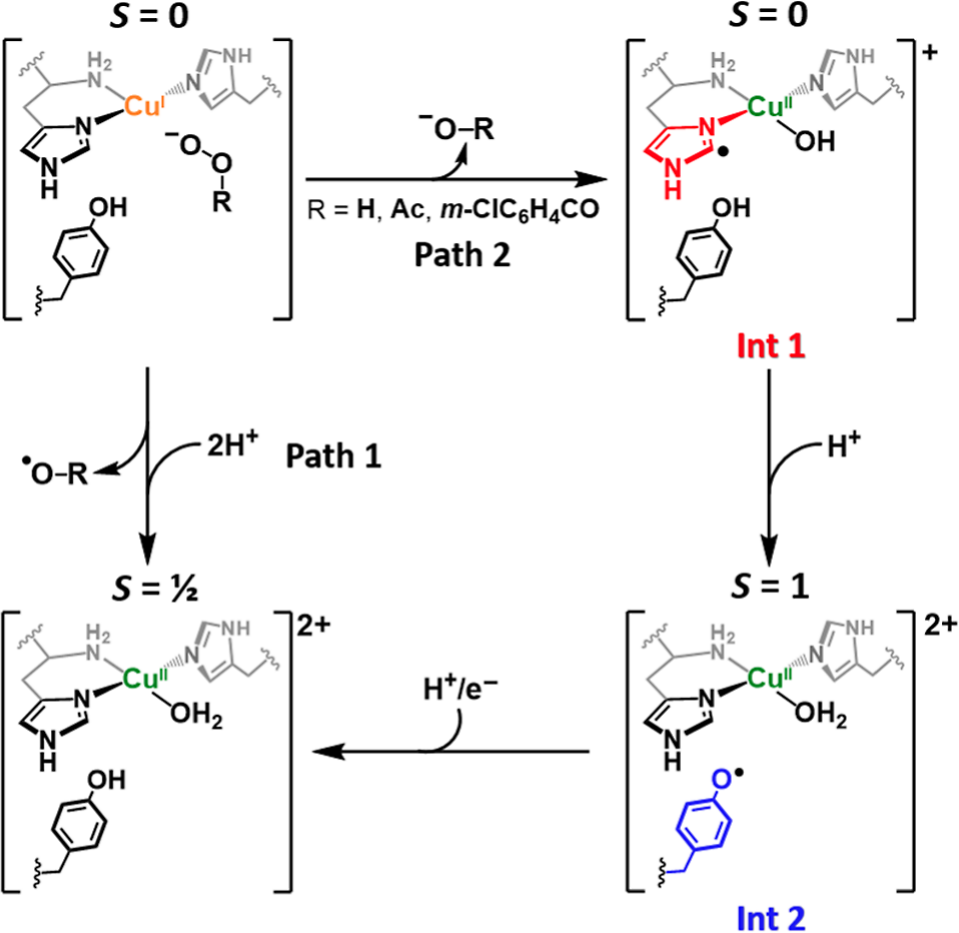
Reaction pathways of Cu^I^–*Ls*AA9 reacting with peroxides in the absence of substrate.

We have demonstrated that **Int1** then oxidizes an adjacent conserved tyrosine residue on a 10–100 ms timescale to give a Cu^II^–tyrosyl radical complex **Int2** (Figure 8). A W64F mutation extends the lifetime of **Int2**, suggesting the tyrosyl radical is reduced by a “hole-hopping” pathway involving Trp64 that likely extends to the exterior of the protein as predicted computationally in an earlier study.^21^ We have also shown that the activity of wild type LPMOs that have oxidized substrate during coupled turnover and then participated in uncoupled turnover can be restored by the addition of reducing agent. In contrast, site-directed mutagenesis studies of the tyrosine to phenylalanine mutant show that the activity of the mutant protein cannot be restored after participating in uncoupled turnover. The details of the protective mechanism in AA9 LPMOs are thus revealed, in which a combination of histidine and an adjacent tyrosine combine to provide a pathway for the dissipation of oxidizing species at the histidine brace active site. These measurements have practical implications when exploiting LPMOs as biocatalysts for lignocellulose deconstruction and show how careful control over the balance of oxidant concentration and reducing equivalents will be important for achieving optimal catalyst performance.

## Conclusions

The ability of LPMO enzymes to mitigate and/or protect against oxidative damage during catalytic turnover clearly offers a competitive advantage to the biomass-degrading organisms which deploy these enzymes in their secretomes. Herein, we have shown that uncoupled turnover of LPMOs with peroxide can rapidly lead to radical formation on coordinating histidine residues, in accord with the known permanent oxidative modification of histidine in LPMOs.^15,21^ In fact, it is perhaps to be expected from the well-known chemistry of Cu^I^ complexes with peroxides that uncoupled reaction of LPMOs with peroxide will lead to oxidative reactions within the primary coordination sphere of the copper histidine brace.^46,47^ Our study shows how biological systems have in-built mechanisms by which this damage can be mitigated. In particular, our results show that a conserved tyrosine residue in the active sites of LPMOs reacts rapidly with a putative Cu^II^–(histidyl radical) to restore it to a Cu^II^–(histidine) complex in an apparent protective mechanism.

The property of LPMOs to protect against oxidative damage in this way is a key component of the enzymes’ ability to operate efficiently in vivo and also speaks to the balance that nature has to strike in utilizing the power of O_2_/peroxide-driven oxidation of substrates. Indeed, this argument is one that extends to the emerging wider discussion about protective pathways within oxygenase enzymes, in which a metal ion is coordinated by one or more histidine residues at the active site, representing the bulk of known metal-containing oxygenases. Necessarily, given the proximity of the histidine to oxidizing equivalents, the concomitant oxidation of coordinated histidine during either coupled or uncoupled turnover of these enzymes could be assumed to be the first and principal site of damage. Thus, internal mechanisms to mitigate the effects of this damage might also be expected to be found across a range of metal-dependent oxygenases. In LPMOs, a combination of histidine alkylation and adjacent redox-active amino acids fulfill such a function, although our study shows that the protective pathway is also competent with an unmodified histidine. It becomes an interesting question as to whether this combination is more widespread, especially given the fact that heterologously expressed enzymes typically used in biochemical studies may not carry the modified histidines. It is also clear that other strategies, such as the addition of excess reducing agents taken to “extinguish” histidyl radicals generated during the turnover of oxygenases, may offer themselves as means of increasing efficiency by prolonging the life of the catalyst,^48^ be it an enzyme or—for that matter—a small molecule mimic. Our studies reported herein on LPMOs can therefore direct future research efforts on maximizing the efficiency of oxidative catalysts.^13^

## Supporting Information Available

Experimental/methods. Stopped-flow kinetic traces and fits. Activity data for Int1, Int2, wild-type protein, and Y164F mutant. EPR spectra and simulations. HERFD-XAS data. DFT data and coordinates of optimized structure of Int1 (PDF)
