# Supplementary material for: Mapping the Initial Stages of a Protective Pathway that Enhances Catalytic Turnover by a Lytic Polysaccharide Monooxygenase
Source: J Am Chem Soc. 2023 Sep 9;145(37):20672–82. doi: 10.1021/jacs.3c06607 (PMC10515631; doi:10.1021/jacs.3c06607)
Supplement: Supplementary file 1 — ja3c06607_si_001.pdf [file ja3c06607_si_001.pdf]

Supplementary Information for

Mapping the Initial Stages of a Protective Pathway that Enhances Catalytic Turnover by a Lytic Polysaccharide Monooxygenase

Jingming Zhao‡[1], Ying Zhuo‡[1], Daniel E. Diaz[2], Muralidharan Shanmugam[1], Abbey J. Telfer[2,4], Peter J. Lindley[2], Daniel Kracher[3], Takahiro Hayashi[1], Lisa S. Seibt[1], Florence J. Hardy[1], Oliver Manners[1], Tobias M. Hedison[1], Katherine A. Hollywood[1], Reynard Spiess[1], Kathleen M. Cain[1], Sofia Diaz-Moreno[4], Nigel S. Scrutton[1], Morten Tovborg,[5] Paul H. Walton*[2], Derren J. Heyes*[1], Anthony P. Green*[1]

[1]Manchester Institute of Biotechnology and Department of Chemistry, University of Manchester, 131 Princess Street, Manchester, M1 7DN, UK

[2] Department of Chemistry, University of York, Heslington, York, YO10 5DD, UK

[3] Institute of Molecular Biotechnology, Graz University of Technology, Petersgasse 14, 8010 Graz, Austria

[4] Diamond Light Source Ltd., Harwell Science and Innovation Campus, Didcot, Oxfordshire OX11 0DE, United Kingdom

[5] Novozymes A/S, Krogshoejvej 36, 2880 Bagsvaerd, Denmark

‡These authors contributed equally to this work.

*Correspondence can be addressed to: anthony.green@manchester.ac.uk; derren.heyes@manchester.ac.uk; paul.walton@york.ac.uk

Experimental/Methods

Materials

H_2O_2 solution (30%), meta-chloroperoxybenzoic acid (*m*-CPBA) and peracetic acid (PAA) were purchased from Sigma-Aldrich and used as received. Cellopentaose (G5) substrate and cellobioase (G2) were purchased from Megazyme Ltd. The fluorescence resonance energy transfer (FRET) substrate (FRET-G4) was synthesized according to the reported procedure.[1] LB agar, 2 x YT auto induction media were purchased from Formedium; *Escherichia coli* (*E. coli*) C43 (DE3), Q5 DNA polymerase, T4 DNA ligase and restriction enzymes from New England BioLabs; and oligonucleotides were synthesized by Integrated DNA Technologies. Buffer solutions were prepared using MQ water.

Preparation of reduced LPMO AA9 enzymes

The fungal *Ta*AA9, *Cv*AA9 and *Ls*AA9 enzymes were prepared according to previously reported procedures.[1,2] The Cu^{I}-states of the above three enzymes were prepared using two methods: 1) mixing 1 equiv. enzyme (100 µM) with 5 equiv. ascorbate (500 µM) in a glove box, followed by buffer exchanging to remove the excess ascorbate and 2) mixing 1 equiv. enzyme (100 µM) with 0.5 equiv. ascorbate (50 µM) in a glove box without further rebuffering. Reduction from Cu(II) to Cu(I) was confirmed by EPR spectroscopy (Figure S55). Subsequent stopped-flow experiments gave essentially identical transients independent of the reduction method employed. Method 2 was employed for subsequent experiments with *E. coli* expressed *Ls*AA9 and its variants unless otherwise stated (see below). For the *Ls*AA9 Y164F variant, 0.7 equiv. ascorbate was needed to achieve complete reduction.

Construction of pET22b_*Ls*AA9 and variants

The coding sequence of fungal *Ls*AA9 (obtained from Prozomix Limited, UK) was codon optimized for *E. coli* expression. To produce the mature LPMO with a catalytic *N*-terminal His1 residue, a pelB leader sequence, which directs the protein secretion to the periplasm and is cleaved off automatically, was ligated to the *N*-terminus of the *Ls*AA9 gene. To achieve high yielding protein purification, a twin-strep-tag sequence (GGSG-WSHPQFEK-GGGSGGGSGGSSA-WSHPQFEK) was ligated to the *C*-terminus (Figure S4). The chimeric DNA sequence was obtained by overlapping PCR using primers E8K_Tt3318A_F (5'-AGATCTTTTAAGAAGGAGATATACATATGAAGTATCTGCTACCTAC-3') and ZYLPMO1R (5'-TCCACGCCGAACCTCCCGATCCACCTCCGGAACCTCCACCTTTCT CGAATTGTGGATGGG-3'). The PCR product was used as template for a 2nd round PCR with the primers E8K_Tt3318A_F and ZYLPMO2R (5'-TACTCGAGTTTGGATCCTTA TTTTTCGAACTGCGGGTGGCTCCACGCCGAACCTCCCGAT-3'). The resulting PCR product was digested by NdeI and XhoI enzymes, and subsequently inserted into the pET22b vector (Invitrogen), to yield the final pET22b_*Ls*AA9 construct. The Y164F, W64F, H147F, H147Q, H147A and Q162A mutations were introduced into the pET22b_*Ls*AA9 construct using QuikChange site-directed mutagenesis. Final DNA sequences were confirmed by DNA sequencing (Source BioScience, Nottingham, UK). The protein sequence and the DNA sequence for wild type *Ls*AA9 were listed in Table S1.

Table S1. The sequence of the pET22b_*Ls*AA9 wild-type construct for periplasmic expression (the two key residues W64 and Y164 is highlighted in bold magenta, the *pel*B sequence is highlighted in blue and the C-terminal twin strep tag is highlighted in orange and green). The associated codon optimized DNA sequence is given below.

protein sequence	MKYLLPTAAAGLLLLAAQPAMA**H**TLVWGVWVNGVDQGDGR<br>NIYIRSPPNNNPVKNLTSPDMTCNVDNRVVPKSVPVNAGDTLTF<br>E**W**YHNTRDDDIIASS**H**HGPIAVYIAPAASNGQGNVWVKLFEDA<br>YNVTNSTWAVDRLITAHGQHSVVVPHVAPGDYLFRAEIIALHE<br>ADSLYSQNPIRGAQF**Y**ISCAQITINSSDDSTPLPAGVPFPGAYTDS<br>TPGIQFNIYTTPATSYVAPPPSVWSGALGGSIAQVGDASLEGGS<br>GWSHPQFEKGGGSGGGSGGSAWSHPQFEK
DNA sequence	ATGAAGTATCTGCTACCTACAGCTGCTGCCGGTCTCTTGTTG<br>TTAGCCGCACAACCTGCAATGGCACACACTCTAGTATGGGGT<br>GTCTGGGTTAATGGAGTTGATCAAGGGGATGGAAGAAATAT<br>CTACATTCGATCACCGCCAAACAACAACCCTGTAAAGAACCT<br>CACGTCTCCTGATATGACTTGTAACGTTGATAATAGAGTTGT<br>TCCTAAAAGCGTACCGGTGAATGCAGGTGATACTCTTACGTT<br>CGAGTGGTACCACAATACGCGAGATGACGACATAATAGCTA<br>GCAGCCACCATGGTCCCATAGCAGTGTATATTGCTCCAGCTG<br>CTTCTAATGGCCAAGGCAATGTTTGGGTTAAGTTGTTTGAAG<br>ATGCTTATAACGTCACCAACTCAACCTGGGCAGTTGATAGAT<br>TGATTACTGCACACGGCCAACATTCTGTCGTCGTTCCTCACG<br>TCGCACCAGGTGACTATCTCTTCCGTGCCGAGATTATTGCTC<br>TACACGAGGCAGATTCATTGTATAGCCAAAATCCAATCAGA<br>GGTGCTCAGTTTTACATCTCTTGTGCTCAGATTACTATTAACT<br>CTTCGGATGATTCTACGCCCCTTCCAGCTGGAGTTCCTTTCCC<br>AGGTGCTTATACTGACAGCACACCAGGTATCCAATTTAACAT<br>ATACACCACTCCGGCTACATCCTACGTTGCTCCTCCTCCAAG<br>TGTCTGGTCAGGAGCTTTGGGTGGATCAATTGCTCAGGTGGG<br>AGACGCTTCACTAGAGGGTGGCTCGGGATGGTCCCATCCAC<br>AATTCGAGAAAGGTGGAGGTTCCGGAGGTGGATCGGGAGGT<br>TCGGCGTGGAGCCACCCGCAGTTCGAAAAA

Protein production and purification

Chemically competent *E. coli* C43 (DE3) cells were transformed with the appropriate LPMO construct (pET22b_*Ls*AA9 WT, Y164F, W64F, H147F, H147Q, H147A and Q162A) and plated onto LB agar containing 100 μg/mL ampicillin. A single colony was used to inoculate 5 mL of LB medium (containing ampicillin 100 μg/mL) and cultured for 18 h. 4 mL of this culture was used to inoculate 400 mL 2 × YT auto induction medium (Formedium, Norfolk, UK) supplemented with 100 μg/mL ampicillin for a 2 h incubation at 37 ˚C, followed by a further incubation of 20 h at 25 ˚C (shaking 180 rpm). The cells were harvested by centrifugation (6000 × g, 4 ˚C, 10 min) and frozen at -20 ˚C until purification.

The frozen cell pellets were thawed on ice for 0.5 h and suspended in 50 mL cold NP buffer (NaH_2PO_4 50 mM, NaCl 300 mM, pH 8.0) containing lysozyme (1 mg/mL) and DNase (10 μg/mL). The mixture was sonicated for 2 × 5 min (1 s on, 1 s off at 50% power) and centrifuged (20,000 × g, 4 ˚C, 1 h). The supernatant was filtered through a 0.2 μm filter and slowly passed through a 5-mL Strep-Tactin®XT column (IBA GmbH, Germany). The column was washed with NP buffer (75 mL) to remove the unbound proteins. Finally, the desired *apo*-*Ls*AA9 was eluted with 18 mL NPB buffer (NaH_2PO_4 50 mM, NaCl 300 mM, biotin 50 mM, pH 8.0). The eluted solution was concentrated by Vivaspin 20 centrifugal concentrators (Generon, Berkshire, UK) with a 10,000 Da molecular weight cut-off membrane, then desalted using a 10DG desalting column (Bio-Rad, Hertfordshire, UK) with potassium phosphate (50 mM, pH 6.0) as the elution buffer. The protein concentration was determined by the absorbance at 280 nm with a Nano-Drop spectrophotometer (ThermoFisher Scientific) using the molecular weight and extinction coefficient calculated by ProtParam tool in ExPASy.

To generate copper-loaded *Ls*AA9 WT, W64F, H147F, H147Q, H147A and Q162A, a solution of $CuCl_2$ (1.1 equiv., 10 mM in Milli-Q water) was slowly added to the above concentrated apo-protein solution followed by incubation on ice for 2h. Excess free copper was removed using a 10DG desalting column with potassium phosphate (50 mM, pH 6.0) as the elution buffer. The Y164F mutant was found to have a weaker affinity for the catalytic copper ion when compared to the wild-type enzyme. It was observed that use of the 10DG desalting column resulted in low concentrations of copper-loaded Y164F. Therefore, to produce the copper-loaded Y164F, the concentrated apo-protein solution was incubated with $CuCl_2$ (0.75 equiv., 10 mM in Milli-Q water) on ice for at least 2 h without any further desalting and concentration steps. The purified proteins were analyzed by SDS-PAGE (Figure S4c), high-resolution mass spectrometry (HRMS) and electron paramagnetic resonance (EPR) spectroscopy, and stored at 4 ˚C prior to use within one week.

Mass spectrometry (MS) analysis

Purified proteins were buffer exchanged into 0.1% acetic acid using a 10 kDa MWCO Vivaspin (Sartorius) and diluted to a final concentration of 0.5 mg/mL. MS was performed using a 1200 series Agilent LC, 5 μL injection into 5% acetonitrile (with 0.1% formic acid), and desalted inline for 1 min. Protein was eluted over 1 min using 95% acetonitrile with 5% water. The resulting multiply charged spectrum was analysed using an Agilent QTOF 6510 and

deconvoluted using Agilent MassHunter Software. The measured protein MS are listed in Table S2.

Table S2. Table of mass spectrometry data of purified *Ls*AA9 variants.

Variant	Expected Mass	Observed Mass
*Ls*AA9 WT	28369.2	28369.0
*Ls*AA9 Y164F	28351.2	28350.9
*Ls*AA9 W64F	28328.2	28327.1
*Ls*AA9 H147F	28379.24	28377.0
*Ls*AA9 H147Q	28360.20	28358.0
*Ls*AA9 H147A	28303.15	28301.0

Stopped-flow kinetics

Stopped-flow kinetic measurements were carried out using an SX20 rapid mixing stopped-flow spectrophotometer (Applied Photophysics Ltd, Leatherhead, UK) placed inside a Belle Technology anaerobic chamber (oxygen levels < 2 ppm) as previously described.[2] Multiple wavelength data were collected at 3 ˚C using a photodiode array (PDA) detector and single wavelength data was obtained from a photomultiplier tube (PMT) single wavelength detector. Most stopped-flow single mixing experiments were peformed at 3 ˚C in potassium phosphate buffer (KPi 50 mM, pH 6.0, degassed overnight before use) using a final concentration of 50 µM protein (reduced by 25 µM ascobate) and 50 µM oxidants (*m*-CPBA, peracetic acid and H_2O_2), or H_2O_2 (500 µM, 2500 µM). For double mixing stopped-flow measurments, reduced Cu^{I}-*Ls*AA9 WT (200 µM, 50 µM final) was first mixed with PAA (1 equiv., 200 µM, 50 µM final) and held in an ageing loop for 50 ms to generate **Int1**, or 1 s to generate **Int2**, before mixing with either buffer (as a control) or G5 substrate (50 µM, 100 µM, 200 µM and 500 µM final concentrations) at pH 6.0, 3 ˚C. Stopped-flow fluorescence measurements using a FRET-G4 substrate were performed using an excitation wavelength of 330 nm and a 455 nm high-pass filter. Stopped-flow samples were freshly prepared by bringing concentrated enzyme stocks, reductant stocks, oxidants stocks, and cellopentaose (G5) substrate stocks inside the N_2 glovebox.

Stopped-flow kinetics fitting

Raw UV/visible absorbance changes from stopped-flow single mixing experiments were fitted globally using the Pro-Kineticist software (Applied Photophysics). The multi-wavelength stopped-flow data for *Ls*AA9 wild type and variants from a range of different experimental conditions were fit to a sequential kinetic model to extract component spectra and global rate constants. The number of phases was determined by visual inspection of the spectra and global residuals. The fit of the model to selected wavelengths is shown in the supporting information in each case. Kinetic transients at single wavelengths from stopped-flow experiments were fitted to single, double or triple exponential equations using the Pro-Data Viewer software.

Quench-flow kinetics

Quench-flow experiments were carried out using an RQF-73 rapid quench-flow instrument (TgK Scientific, UK) placed inside a Belle Technology anaerobic chamber (oxygen levels < 2 ppm). All quench-flow double mixing experiments were performed at 3˚C in KPi (50 mM, pH 6.0, degassed overnight before use). The reduced Cu^{I}-*Ls*AA9 WT (200 µM, 50 µM final) was first mixed with *m*-CPBA (1 equiv., 200 µM, 50 µM final) and held in an ageing loop for 50 ms to generate **Int1**, or 1 s to generate **Int2**, before mixing with G5 substrate (5 equiv., 250 µM final concentration) at pH 6.0 at 3˚C. The final reaction mixture (200 µL) was recovered from the instrument and immediately quenched with MeCN (200 µL). The mixture was centrifuged at 10 000 rpm for 10 min and the supernatant was subjected to LC-MS analysis.

Liquid Chromatography- Mass Spectrometry

All analysis was conducted on a QExactive Plus with an Ultimate 3000 UHPLC (Thermo, UK). The UHPLC was equipped with a ZIC-cHILIC column (2.1 mm x 100 mm; 3 mm particle size). The solvents employed were (A) water + 0.1% formic acid and (B) acetonitrile + 0.1 % formic acid. The flow gradient was programmed to equilibrate at 99% B for 2 min followed by a linear gradient to 10% B over 10 min and held at 10% B for 2 min before returning to 99% B for 2 min with a flow rate of 600 mL/min. The column was maintained at 40 °C and the samples chilled in the autosampler at 10 °C. A sample volume of 5 µL was injected onto the column. Data acquisition was conducted in full scan mode in the scan range of 90-1050 m/z with a resolution of 70,000, an AGC target of $3e^{6}$ and a maximum integration time of 200 ms. The acquisition was conducted in positive ion mode.

Electron paramagnetic resonance (EPR)

EPR measurements were carried out using a Bruker ELEXSYS-E580 X-band EPR spectrometer capable of operating both in continuous wave (CW) and pulsed modes, equipped with an Oxford variable-temperature unit and ESR900 cryostat with Super High-Q resonator. All EPR samples were prepared in quartz capillary tubes (outer diameter; 4.0 mm, inner diameter 3.0 mm) and frozen immediately in liquid nitrogen until further analysis. The X-band EPR tubes were then transferred into the EPR probe-head, which was pre-cooled to 20 K. A microwave power of 30 dB (0.2 mW) and modulation of 5 G appear to be optimal for recording the EPR spectra of the *Ls*AA9 and its variants. The low temperature EPR spectra were acquired using the following conditions: sweep time of 84 s, microwave power of 0.2 mW, time constant of 81 ms, average microwave frequency of 9.386 GHz and modulation amplitude of 5 G. The intermediates were formed using 200 µM protein with 100 µM ascorbate and 200 µM PAA in 50 mM KPi buffer (pH 6.0). Samples were prepared using a home-made hand mixing device with two syringes, one containing reduced protein and the other containing PAA, that are directly mixed into an EPR tube and quickly frozen in liquid nitrogen. Analysis and simulations of the CW-EPR spectra were performed using EasySpin toolbox (5.2.28) for the /Matlab (R2017a) program package.[3]

X-ray absorption spectroscopy (XAS) and freeze quench method

*Ls*AA9 Y164F (0.8 mM) in KPi buffer (1.5 mL, 50 mM, pH 6) was loaded with Cu(II) (0.8 equiv., 10 mM $Cu(NO_3)_2$) and reduced under anaerobic conditions using sodium ascorbate to a final concentration of Cu(I) *Ls*AA9 Y164F of 0.64 mM. Cu(I) *Ls*AA9 Y164F (1.5 mL, 0.64 mM) was loaded along with m-CPBA (15 µL, 64 mM) into separate syringes and cooled to 3 °C in the Biologic SFM 2000. The solutions were programmed to mix in a 1:1 molar ratio at 100 ms and 350 ms before being ejected into liquid N_2. The final copper concentration on the samples was approximately 0.63 mM. The samples were then ground into a fine powder in liquid N_2 to be packed into pure aluminium sample holders for the XAS measurements. The thickness of the sample was 2 mm.

The HERFD-XAS experiments were performed at beamline I20-Scanning[4] at Diamond Light Source (Didcot, United Kingdom). At the time of the measurements the synchrotron was operating with a ring energy of 3 GeV and at a current of 300 mA. The beamline was equipped with an in-house designed four-bounce scanning Si(111) monochromator[5] and two dedicated Rh-coated mirrors operating at 4.5 mrad incidence angle were used to reject higher harmonics. The beam size at the sample was 400 µm (H) x 300 µm (V) FWHM. The X-ray beam was attenuated using a 0.2 mm thick aluminium foil, in order to mitigate photoreduction, so the photon flux at the sample was estimated to be $1x10^{11}$ ph/s. The x-ray emission spectrometer[6] available in the beamline, and based on a 1 m diameter Rowland circle operating in the Johann configuration in the vertical plane[7] was used for the experiment.

The Cu Kα1 emission line (8048 eV) was collected using three 100 mm diameter Si(444) spherically bent analyzer crystals. An ionization chamber filled with the appropriate mixture of He and Ar gases to absorb 20% of the beam was used as an incident intensity monitor, while a four element Medipix-Merlin photon-counting pixel detector[8] was used to monitor the Kα1 emission line. The energy resolution of the measurements was 1 eV, calculated from the width of the elastic peak measurements.

During data collection, samples were held at 10 K (140 K during sample transfer) using a liquid He cryostat, to curb photoreduction radiation damage induced photoreduction. Each six-minute scan was collected at different points in the sample, to avoid photoreduction.

All XANES spectra for the respective samples were processed *via* calibration, alignment, background removal and normalisation and subsequently merged to improve signal-to-noise ratio using Athena v 0.9.26, part of the Demeter software[9] following standard procedures.

DFT calculations

Geometry optimizations were performed using the ORCA 4.2 software package to the DFT level of theory.[10,11] Starting point geometries of the resting state *Ls*AA9 enzyme were obtained from crystallographic coordinates (PDB: 5ACI).[1] The crystallographic coordinates were truncated to only include the central copper ion, coordinating oxygen and certain key residues: His-1 and His-78 making up the 'histidine brace', Tyr-164 and Gln-162. Hydrogen atoms were added to suitable positions to complete the valence shells of each atom from the crystallographic coordinates. To further reduce the size of the model the selected residues were further truncated *via* the following modifications: The carbonyl of His-1 was substituted with a methyl group; His-78 and Tyr-164 were truncated by methyl substitution of the C_β carbon; Gln-162 was

truncated by methyl substitution of the C_γ carbon. For the histidyl radical calculation no hydrogen atom was added to the C2 position of His-1.

Geometry optimizations were performed with the BP86[12] functional (with RI approximation), Def2-TZVP basis set[13] on Cu and ligating atoms and Def-2-SVP on all the remaining atoms; empirical dispersion correction were accounted using Grimme's D3 method with Becke-Johnson damping (D3BJ)[14]; solvation effects were included with the conductor-like polarizable continuum model (CPCM, ε=4.0)[15]. The broken symmetry (BS) approach was used to optimize the singlet spin state geometry. Single point energies were calculated using the B3LYP functional[16] and the Def2-TZVP basis set on all atoms. Corrected singlet state energies and exchange coupling constants (*J*) were computed with the Yamaguchi formula[17]:

$$J = \frac{E_{triplet} - E_{BS\ singlet}}{< S^2 >_{triplet} - < S^2 >_{singlet}}$$

Cu K-edge absorption spectra were calculated with time dependent density functional theory (TD-DFT) using ORCA 4.2 software approach applying the Tamm−Dancoff approximation.[18] The XAS absorption spectra were computed with RIJCOSX[19] approximation with a dense integration grid, using the uB3LYP functional and ZORA-def2-TZVP basis set[20] on all atoms. The Zeroth order relativistic approximation (ZORA) was employed to better account for relativistic effects; deemed important for this type of calculation.[21] The SARCJ auxiliary basis set was also used to give improved accuracy on relativistic calculations than that of def2J. The integration grid size was increased to its maximum of 7. Solvation effects were accounted for using the Conductor-like Polarizable Continuum Model (CPCM), ε = 80.0 and refractive index = 1.33. 30 transitions (roots) were calculated starting from the Cu 1*s* orbitals. The lowest energy peak calculated in both models corresponds to a weak quadrupole allowed 1*s*-3*d* transition (final state occupying the Cu hole), the next is a weak 1*s*→2*p* MLCT (final state occupying a 2*p* orbital on the C2 carbon atom of His-1), followed finally by the rising edge of 1*s*→4*p*/continuum.

Supplementary figures and tables

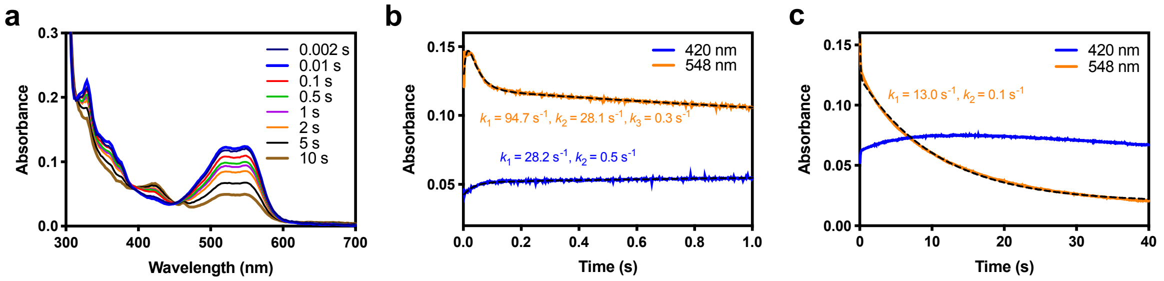

Figure S1. Stopped-flow analysis of *Ta*AA9 oxidation. Cu^{I}-*Ta*AA9 (100 µM) mixed with *m*-CPBA (500 µM) in MES buffer (50 mM, pH 6) at 3 ˚C. (a) Raw UV-vis absorbance spectra at selected time points. (b) Kinetic transients at 420 nm and 548 nm over 1 s. The transient at 420 nm was fitted to a double exponential model and the transient at 548 nm was fitted to a triple exponential model, yielding rates of formation of 28.2 s^{-1} for the tyrosyl radical and 94.7 s^{-1} for the tryptophanyl radical, respectively. The number of phases was determined by inspection of the residuals from each fit. (c) Kinetic transients at 420 nm and 548 nm over 40 s, showing the full decay of the tryptophanyl radical with rates of 13.0 s^{-1} and 0.1 s^{-1}. Black dashed lines show the fits of the data to the exponentials described.

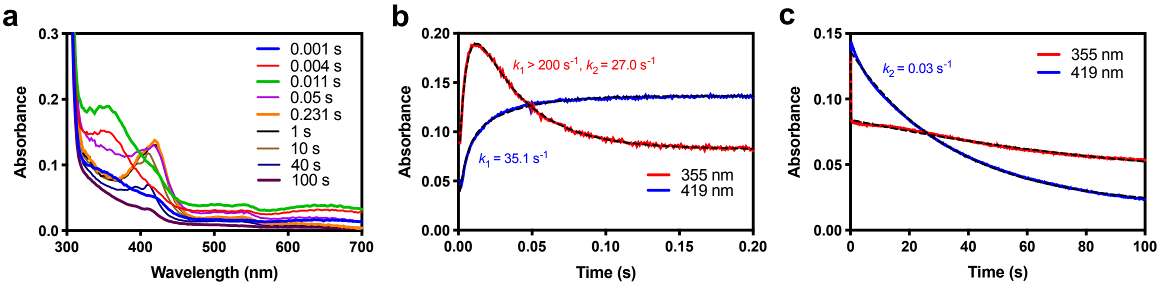

Figure S2. Stopped-flow analysis of *Cv*AA9 oxidation. Cu^{I}-*Cv*AA9 (100 µM) mixed with m-CPBA (500 µM) in MES buffer (50 mM, pH 6) at 3 ˚C. (a) Raw UV-vis absorbance spectra at selected time points. (b) Kinetic transients at 355 nm and 419 nm over 1 s. The transient at 355 nm was fitted to a double exponential model, yielding rates of formation and decay of **Int1** of > 200 s^{-1} and 27.0 s^{-1} respectively. The transient at 419 nm was fitted to a single exponential model to yield a rate of formation of **Int2** of 35.1 s^{-1}. (c) Kinetic transients at 355 nm and 419 nm, showing the decay of **Int2** over 100 s. The transient at 419 nm was fitted to a single exponential, giving the decay rate of 0.03 s^{-1}. All transients are overlaid with fits by black dashed lines.

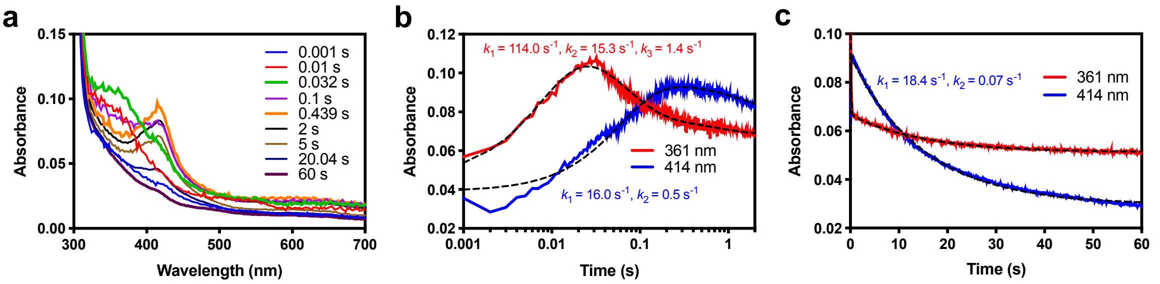

Figure S3. Stopped-flow analysis of fungal *Ls*AA9 oxidation. Cu^{I}-*Ls*AA9 (100 µM, expressed in *Aspergillus oryzae*) mixed with *m*-CPBA (100 µM) in Kpi buffer (50 mM, pH 6) at 3 ˚C. (a) Raw UV-vis absorbance spectra at selected time points. (b) Kinetic transients at 361 nm and 414 nm over 2 s. The transient at 361 nm was fitted to a triple exponential, yielding a formation rate of **Int1** of 114 s^{-1}, and the decay rates 15.3 s^{-1} and 1.4 s^{-1}. The transient at 414 nm was fitted to a double exponential, yielding a formation rate of **Int2** of 16.0 s^{-1} and the decay rate 0.5 s^{-1}. (c) Kinetic transients at 361 nm and 414 nm over 60 s. The transient at 414 nm was fitted to a double exponential to yield a rate of formation of **Int2** of 18.4 s^{-1} and a rate of decay of 0.07 s^{-1}. All transients are overlaid with fits by black dashed lines.

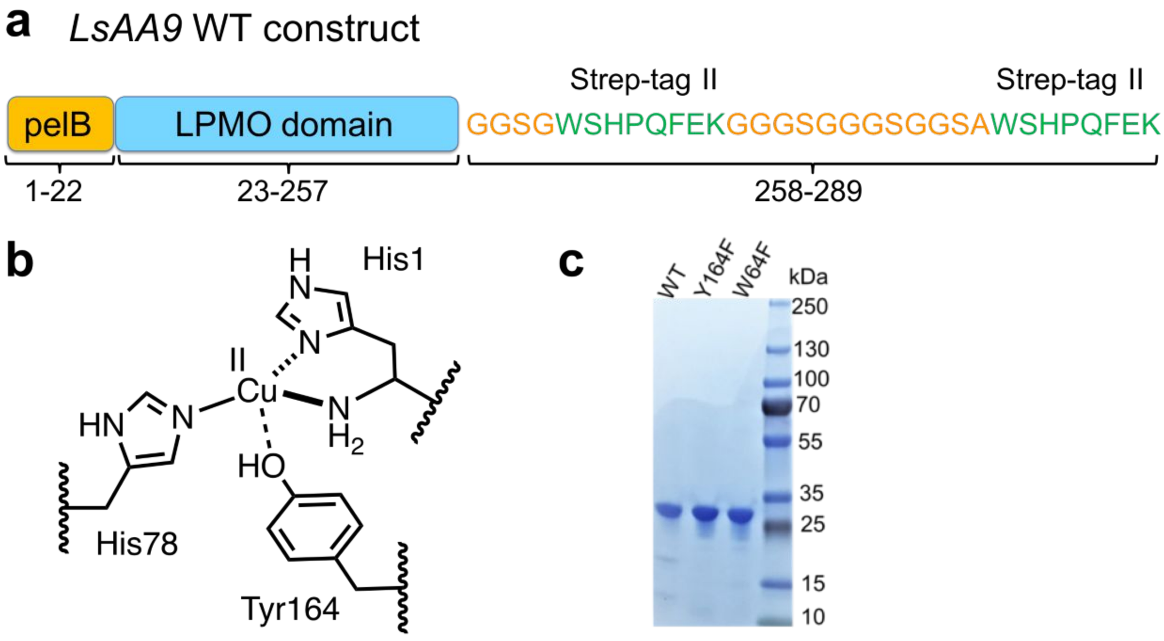

Figure S4. The construction and characterization of *Ls*AA9. (a) The full-length *Ls*AA9 genetic construct, featuring a *pel*B sequence for the periplasmic secretion, an *Ls*AA9 domain and a twin-strep-tag for affinity purification. (b) The histidine brace active site of the mature *Ls*AA9. (c) SDS-PAGE analysis of purified *Ls*AA9 variants.

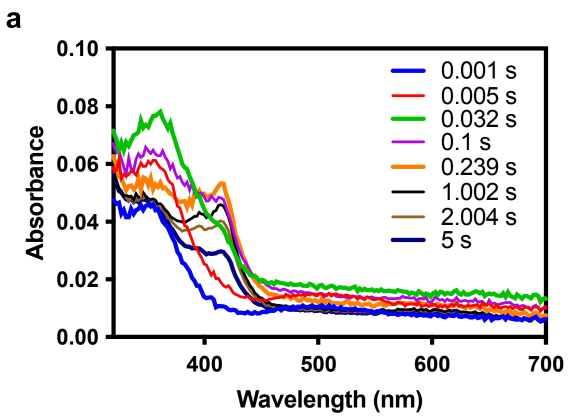

Figure S5. Stopped-flow analysis of *E. coli Ls*AA9 oxidation. $\mathrm{Cu^{I}}$-*Ls*AA9 (100 μM) mixed with *m*-CPBA (100 μM) in KPi buffer (50 mM, pH 6) at 3 ˚C. (a) Raw UV-vis absorbance spectra at selected time points. (b) Kinetic transients at 361 nm and 414 nm over 5 s. The transient at 361 nm was fitted to a triple exponential, yielding a rate of formation of **Int1** 111.9 s^{-1} and decay rates of 8.9 s^{-1} and 0.6 s^{-1}. The transient at 414 nm was fitted to a double exponential, yielding rates of formation and decay of **Int2** of 19.6 s^{-1} and 0.3 s^{-1} respectively. All transients are overlaid with fits by black dashed lines.

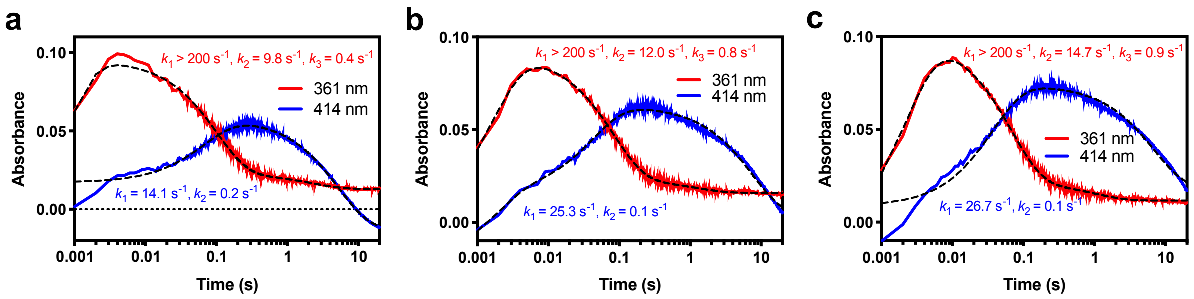

Figure S6. Stopped-flow kinetics for reoxidation of $\mathrm{Cu^{I}}$-*Ls*AA9 (100 μM) mixed with *m*-CPBA (500 μM) in KPi buffer (50 mM) at different pHs at 3 ˚C. (a) pH 6; (b) pH 7; (c) pH 8. The kinetic transients of **Int1** (red) are fitted to a triple exponential and **Int2** transients (blue) are fitted to a double exponential. The number of phases was determined by inspection of the residuals from each fit. The reaction rates are listed on each panel. All transients are overlaid with fits by black dashed lines. The spectra of Int1 and Int2 are almost indistinguishable across the pH range tested.

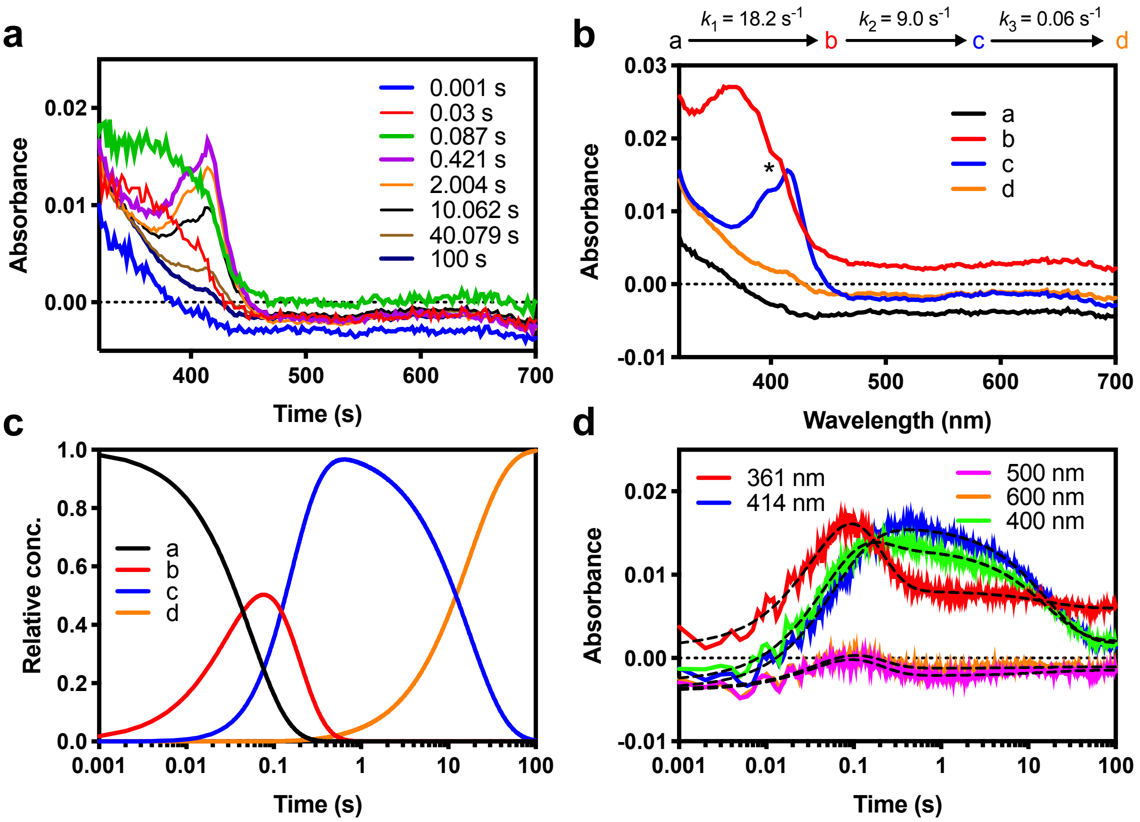

Figure S7. Stopped-flow kinetics for reoxidation of Cu^{I}-*Ls*AA9 WT (50 µM) mixed with H_2O_2 (10 equiv., 500 µM) in MES buffer (50 mM, pH 6.0) at 3 ˚C. (a) Raw UV-visible absorbance spectra at selected time points from 0.001 to 100 s after mixing. (b) Global fitting of the raw UV-visible kinetic data using a sequential a-b-c-d model gives rates of $k_1 = 18.2 \pm 0.1$ s^{-1}, $k_2 = 9.0 \pm 0.04$ s^{-1}, $k_3 = 0.06 \pm 0.001$ s^{-1}. The asterisk illustrates a small amount of the 414 nm absorbance feature present in species b. (c) Concentration profiles of a, b, c and d species generated from the global analysis. (d) Kinetic transients at selected wavelengths, overlaid by the fits derived from the global analysis (black dashed lines).

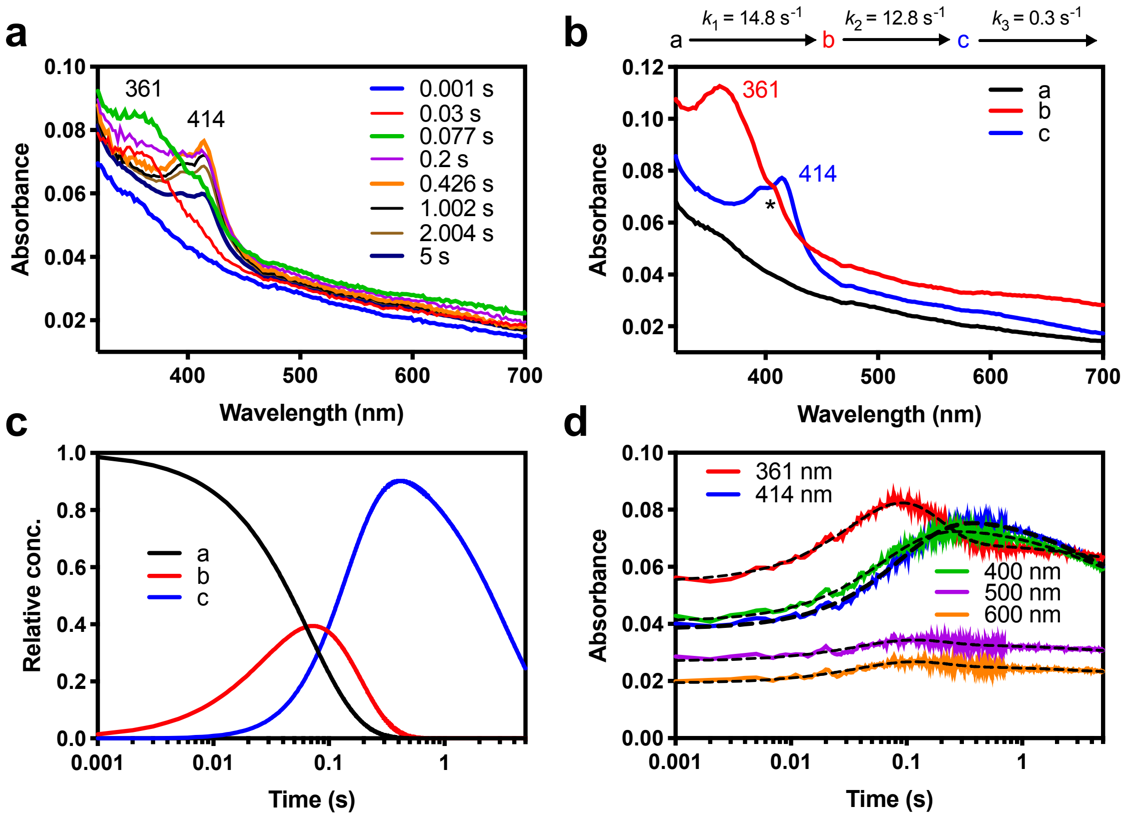

Figure S8. Stopped-flow kinetics for reoxidation of Cu^{I}-*Ls*AA9 WT (50 μM) mixed with H_2O_2 (10 equiv., 500 μM) in KPi buffer (50 mM, pH 6) at 3 ˚C. (a) Raw UV-visible absorbance spectra at selected time points. (b) Global fit of the raw UV-visible kinetic data using a sequential a-b-c-d model gives rates of $k_1 = 14.8 \pm 0.2$ s^{-1}, $k_2 = 12.8 \pm 0.1$ s^{-1}, $k_3 = 0.3 \pm 0.002$ s^{-1}. The asterisk illustrates a small amount of the 414 nm absorbance feature present in species b. (c) Concentration profiles of a, b and c species from the global analysis. Species c decays to a species d with no obvious spectral features (see Fig. S7). (d) Kinetic transients at selected wavelengths, overlaid by the fits derived from the global analysis (black dashed lines).

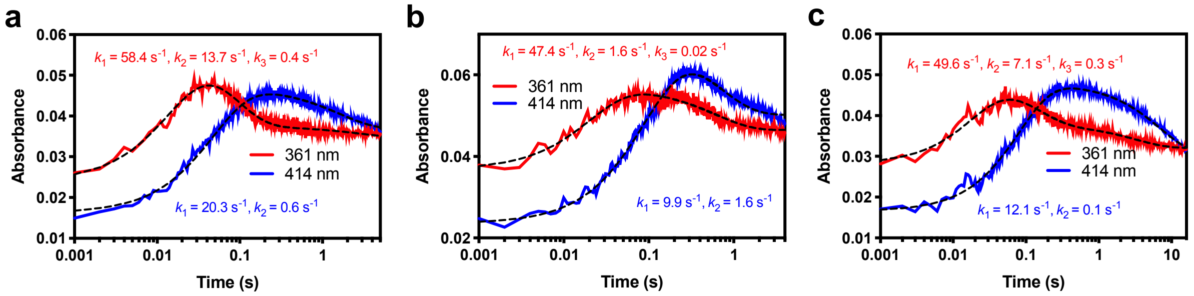

Figure S9. Stopped-flow kinetics for reoxidation of Cu^{I}-*Ls*AA9 (50 μM) mixed with *m*-CPBA (50 μM) in KPi buffer (50 mM, pH 6) at 3 ˚C. To prepare Cu^{I}-*Ls*AA9 (50 μM), the resting state *Ls*AA9 (50 μM) was reduced by different reductants (50 μM) and desalted. (a) ascorbate; (b) dithionite; (c) pyrogallol. The kinetic transients of **Int1** (red) are fitted to a triple exponential and **Int2** transients (blue) are fitted to a double exponential. The reaction rates are listed on each panel. All transients are overlaid with fits by black dashed lines.

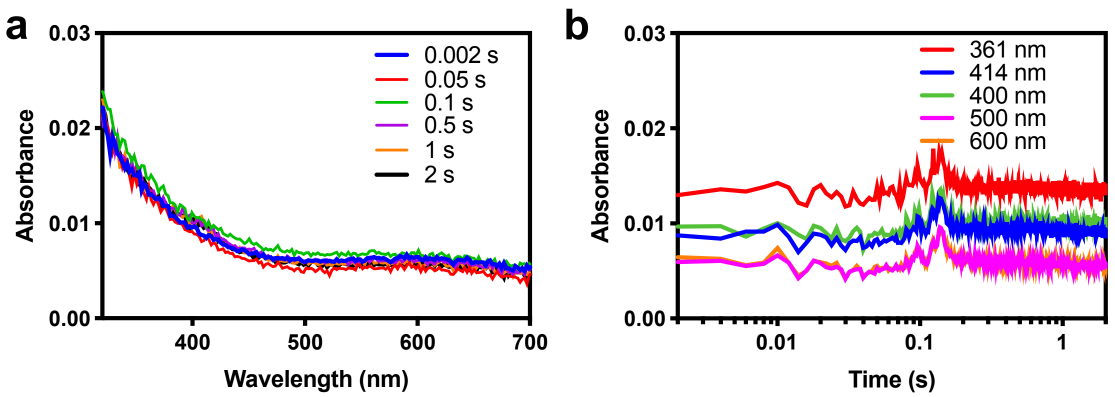

Figure S10. Stopped-flow kinetics for resting state Cu^{II}-*Ls*AA9 WT (50 µM) mixed with *m*-CPBA (1 equiv., 50 µM) in KPi buffer (50 mM, pH 6) at 3 ˚C. (a) Raw UV-visible absorbance spectra at selected time points. (b) Kinetic transients at selected wavelengths.

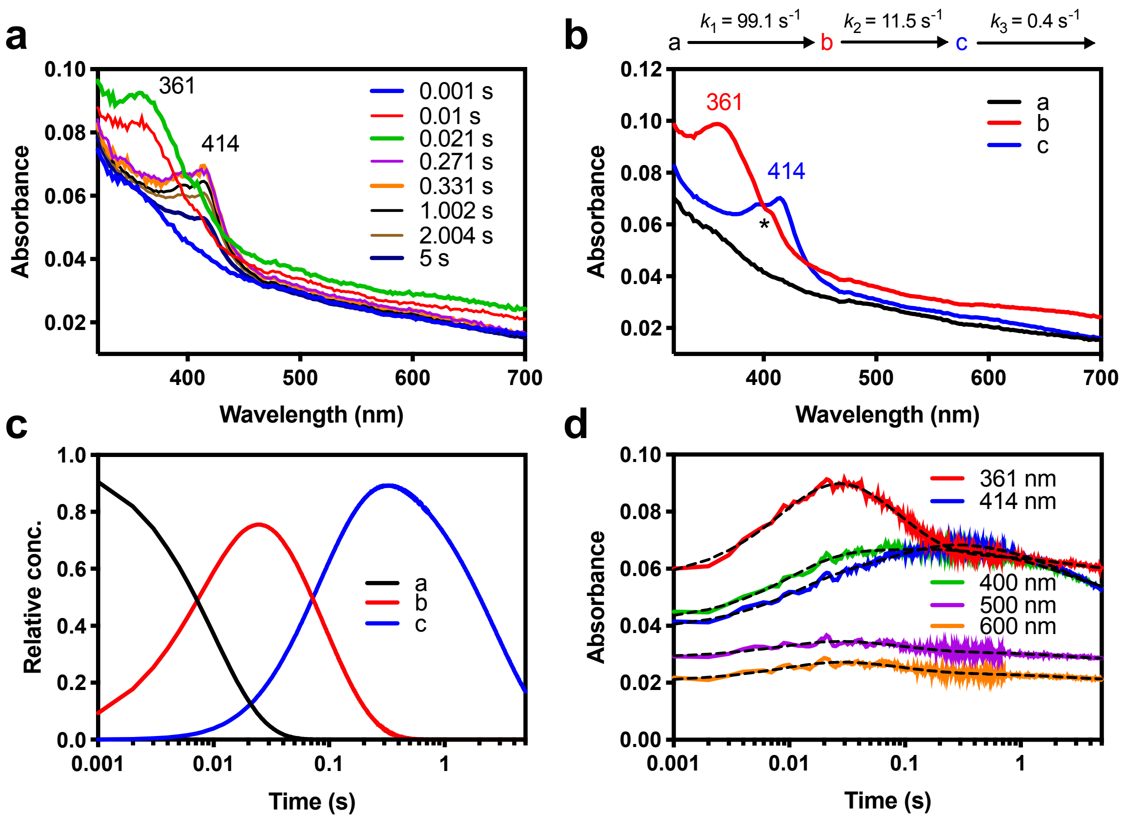

Figure S11. Stopped-flow kinetics for reoxidation of Cu^{I}-*Ls*AA9 WT (50 μM) mixed with H_2O_2 (50 equiv., 2500 μM) in KPi buffer (50 mM, pH 6) at 3 ˚C. (a) Raw UV-visible absorbance spectra at selected time points. (b) Global fit of the raw UV-visible kinetic data using a sequential a-b-c-d model gives rates of $k_1 = 99.1 \pm 0.3$ s^{-1}, $k_2 = 11.5 \pm 0.02$ s^{-1}, $k_3 = 0.4 \pm 0.002$ s^{-1}. The asterisk illustrates a small amount of the 414 nm absorbance feature present in species b. (c) Concentration profiles of a, b and c species from the global analysis. (d) Kinetic transients at selected wavelengths, overlaid by the fits derived from the global analysis (black dashed lines).

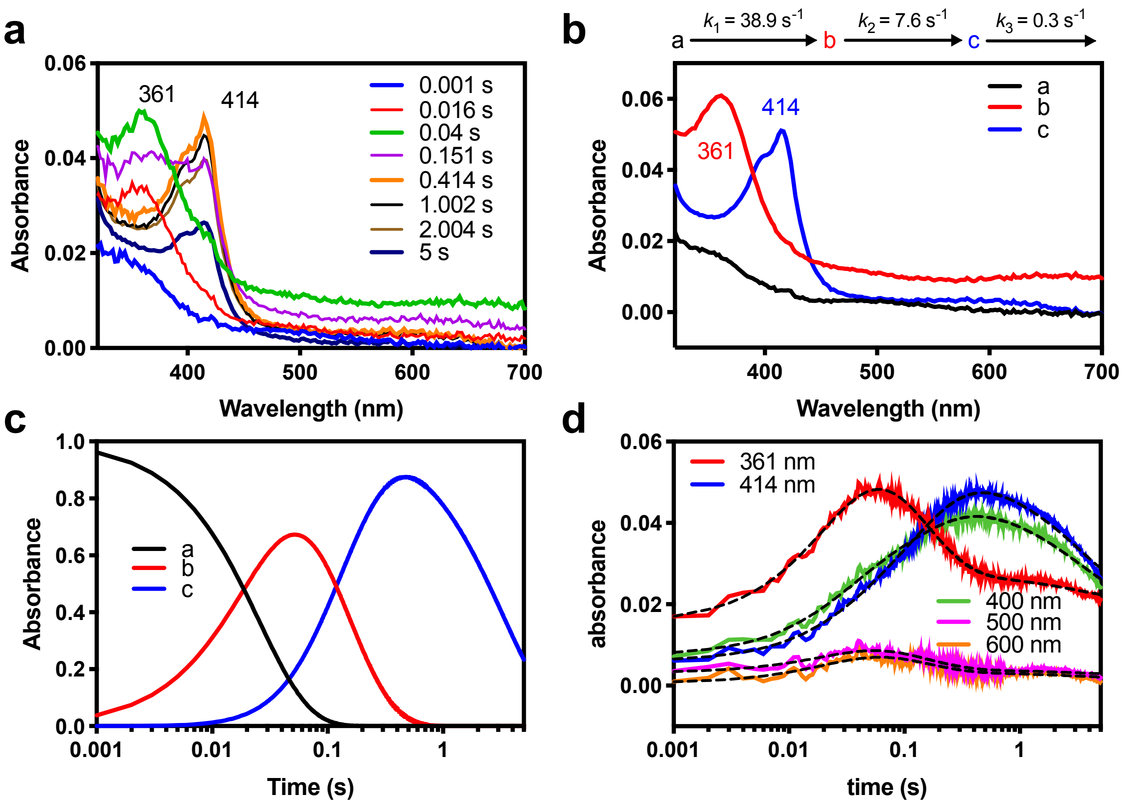

Figure S12. Stopped-flow kinetics (5 s) for reoxidation of Cu^{I}-*Ls*AA9 WT (50 μM) mixed with peracetic acid (1 equiv., 50 μM) in KPi buffer (50 mM, pH 6) at 3 ˚C. (a) Raw UV-visible absorbance spectra at selected time points. (b) Global fit of the raw UV-visible kinetic data using a sequential a-b-c-d model gives rates of $k_1 = 38.9 \pm 0.1$ s^{-1}, $k_2 = 7.6 \pm 0.01$ s^{-1}, $k_3 = 0.3 \pm 0.001$ s^{-1}. (c) Concentration profiles of a, b and c species from the global analysis. (d) Kinetic transients at selected wavelengths, overlaid by the fits derived from the global analysis (black dashed lines).

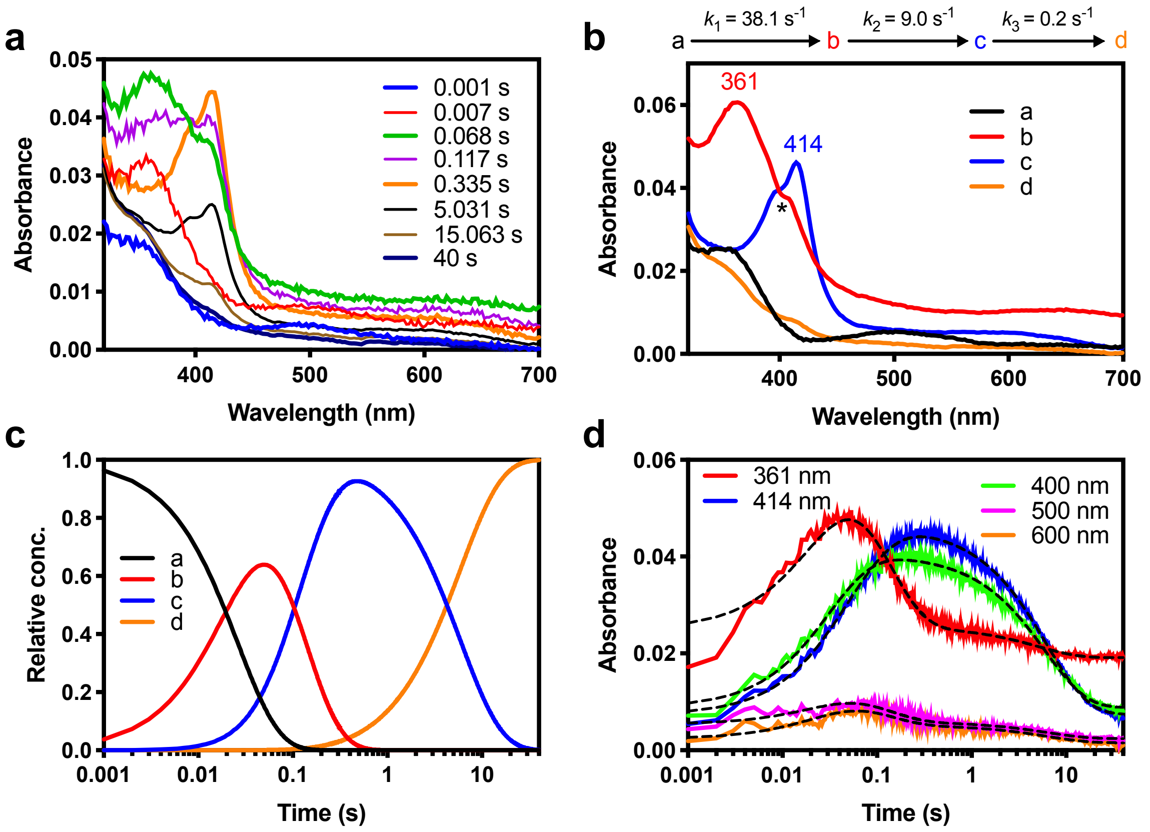

Figure S13. Stopped-flow kinetics for reoxidation of Cu^{I}-*Ls*AA9 WT (50 μM) mixed with *m*-CPBA (1 equiv., 50 μM) in KPi buffer (50 mM, pH 6) at 3 ˚C. (a) Raw UV-visible absorbance spectra at selected time points. (b) Global fit of the raw UV-visible kinetic data using a sequential a-b-c-d model gives rates of k_1 = 38.1 ± 0.1 s^{-1}, k_2 = 9.0 ± 0.01 s^{-1}, k_3 = 0.2 ± 0.001 s^{-1}. The asterisk illustrates a small amount of the 414 nm absorbance feature present in species b. (c) Concentration profiles of a, b and c species from the global analysis. (d) Kinetic transients at selected wavelengths, overlaid by the fits derived from the global analysis (black dashed lines).

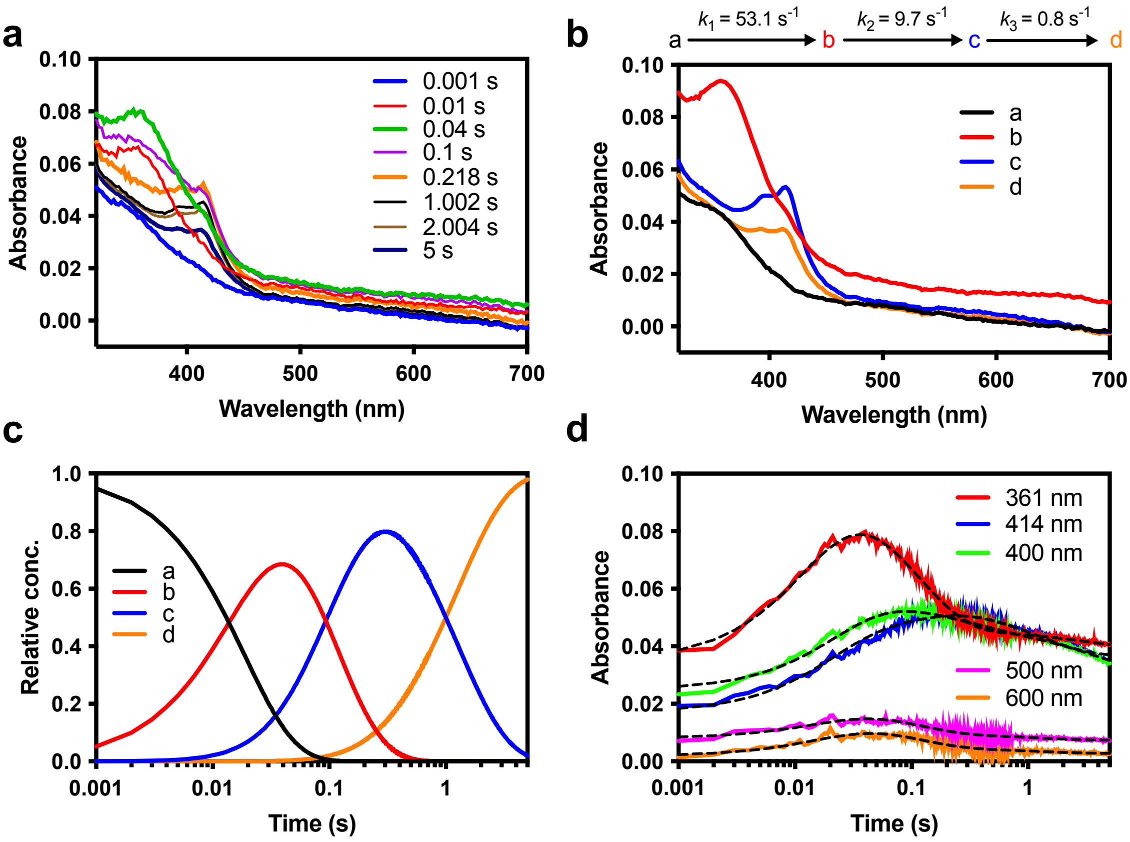

Figure S14. Stopped-flow kinetics (5 s) for reoxidation of Cu^I-*Ls*AA9 WT (1 equiv., 50 µM) mixed with peracetic acid (2 equiv., 100 µM) in KPi buffer (50 mM, pH 6) at 3 ˚C. (a) Raw UV-visible absorbance spectra at selected time points. (b) Global fit of the raw UV-visible kinetic data using a sequential a-b-c-d model gives rates of $k_1 = 53.1 \pm 0.1\ s^{-1}$, $k_2 = 9.7 \pm 0.02\ s^{-1}$, $k_3 = 0.8 \pm 0.003\ s^{-1}$. (c) Concentration profiles of a, b, c and d species from the global analysis. (d) Kinetic transients at selected wavelengths, overlaid by the fits derived from the global analysis (black dashed lines).

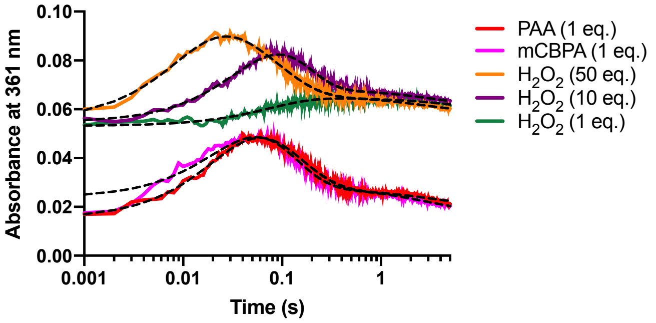

Conditions	**Int1** formation rate (s^{-1})
PAA (1 eq.)	38.9 ± 0.1
PAA (2 eq.)	53.1 ± 0.1
m-CPBA (1 eq.)	38.1 ± 0.1
H_2O_2 (50 eq.)	99.1 ± 0.3
H_2O_2 (10 eq.)	14.8 ± 0.2
H_2O_2 (1 eq.)	–

Figure S15. Stopped-flow analysis of *Ls*AA9 oxidation using different oxidants. Cu^{I}-*Ls*AA9 (50 µM) was mixed with various oxidants in KPi buffer (50 mM, pH 6) at 3 ˚C. Kinetic transients of **Int1** at 361 nm are overlaid with fits by black dashed lines. **Int1** formation rates are listed in the table.

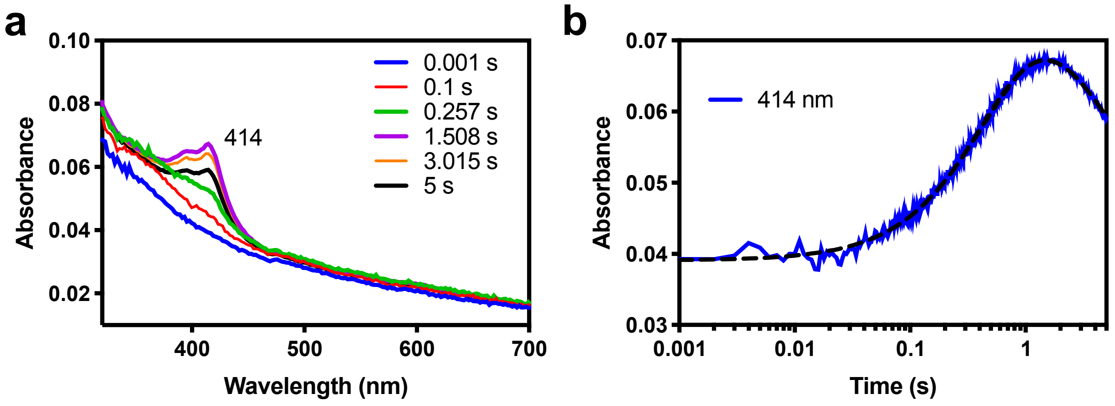

Figure S16. Stopped-flow kinetics for reoxidation of Cu^{I}-*Ls*AA9 WT (50 µM) mixed with H_2O_2 (1 equiv., 50 µM) in KPi buffer (50 mM, pH 6) at 3 ˚C. (a) Raw UV-visible absorbance spectra at selected time points. (b) The kinetic transient at 414 nm was fitted to a double exponential equation (black dashed lines), affording the **Int2** formation rate (k = 2.1 ± 0.03 s^{-1},) and decay rate (k = 0.1 ± 0.03 s^{-1}).

Conditions	**Int1** formation rate (s^{-1}) in KPi (50 mM in D_2O, pD 6)	**Int1** formation rate (s^{-1}) in KPi (50 mM in H_2O, pH 6)	KIE
PAA (1 eq.)	22.7	28.9	1.3
m-CPBA (1 eq.)	15.6	24.2	1.6
H_2O_2 (50 eq.)	33.5	56.9	1.7
H_2O_2 (10 eq.)	20.4	33.6	1.6
Conditions	**Int2** formation rate (s^{-1}) in KPi (50 mM in D_2O, pD 6)	**Int2** formation rate (s^{-1}) in KPi (50 mM in H_2O, pH 6)	KIE
PAA (1 eq.)	5.6	8.4	1.5
m-CPBA (1 eq.)	7.2	9.7	1.3
H_2O_2 (50 eq.)	5.0	11.2	2.2
H_2O_2 (10 eq.)	6.8	13.4	2.0

Table S3. Stopped-flow analysis of *Ls*AA9 oxidation showing a solvent Kinetic Isotope Effect (KIE). Cu^{I}-*Ls*AA9 (50 µM) was mixed with various oxidants in KPi buffer (50 mM in D_2O or H_2O) at 5 ˚C. **Int1** formation rates are listed in the table. KIE = (**Int1** formation rate in H_2O)/ (**Int1** formation rate in D_2O).

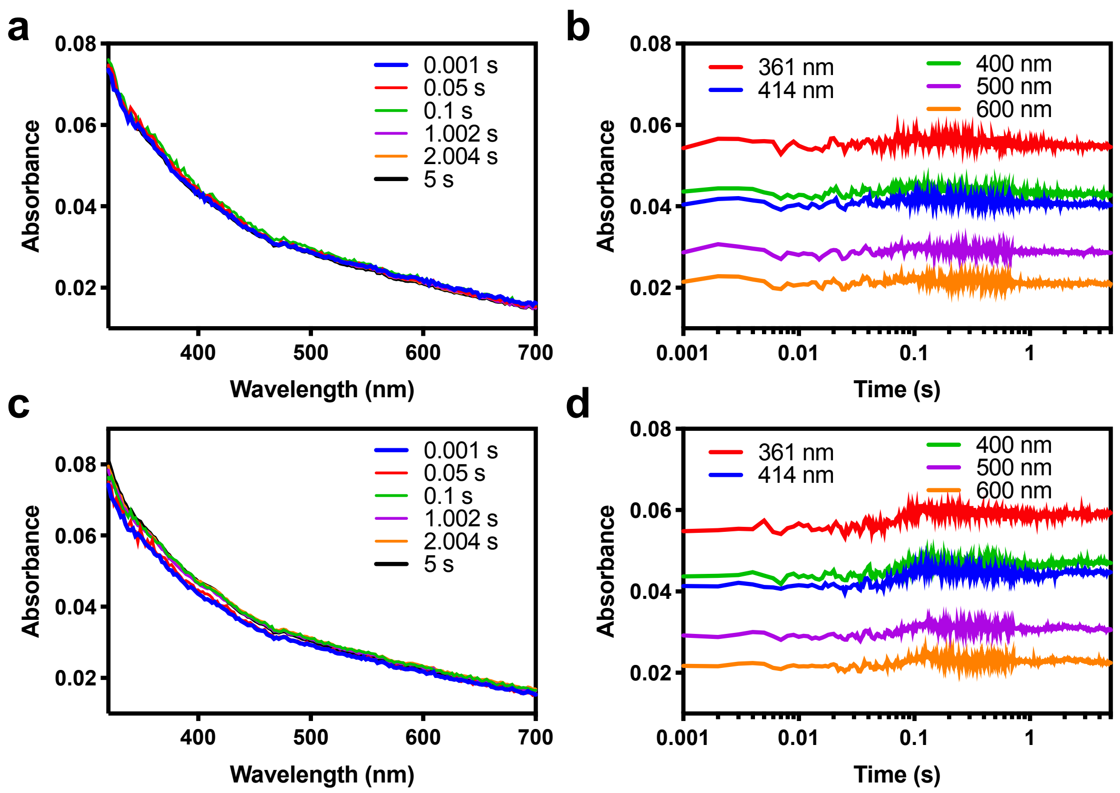

Figure S17. Stopped-flow kinetics for reoxidation of Cu^{I}-*Ls*AA9 WT (50 µM), pre-bound with G5 substrate (500 µM), mixed with H_2O_2 (1 equiv., 50 µM, a and b) or PAA (1 equiv., 50 µM, c and d) in KPi buffer (50 mM, pH 6) at 3 ˚C. (a and c) Raw UV-visible absorbance spectra at selected time points. (b and d) Kinetic transients at selected wavelengths.

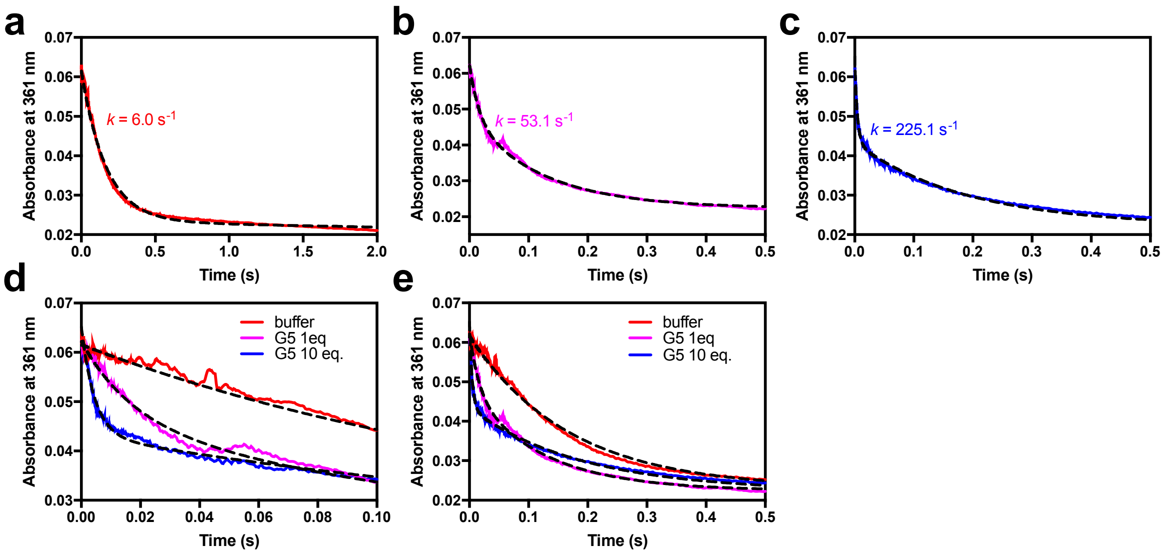

Figure S18. Double-mixing stopped-flow kinetics using a photomultiplier tube detector (361 nm). Reduced Cu^{I}-*Ls*AA9 WT (50 μM) was first mixed with PAA (1 equiv., 50 μM), allowed to age for 50 ms (**Int1** is fully formed), then mixed with either buffer (as a control) or G5 substrate (1 equiv. 50 μM) or G5 (10 equiv., 500 μM) in KPi buffer (50 mM, pH 6) at 3 ˚C. Data were fitted to a double exponential equation to generate the following k_1 decay rates of **Int1**: (a) 6.0 ± 0.1 s^{-1} for buffer, (b) 53.1 ± 1.5 s^{-1} for G5 1 eq. and (c) 225.1 ± 3.2 s^{-1} for G5 10 eq. (d) The absorbance at 361 nm over 0.1 s after mixing with buffer or G5 (1 and 10 eq.). (e) The absorbance at 361 nm over 0.5 s after mixing with buffer or G5 (1 and 10 eq.). All fits are shown as black dashed lines.

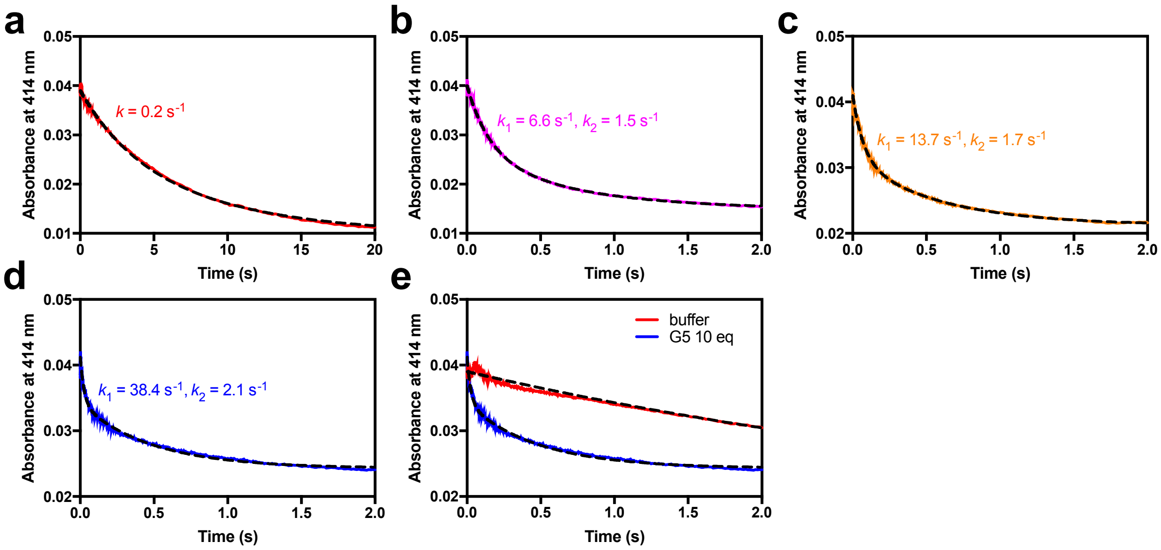

Figure S19. Double-mixing stopped-flow kinetics using a photomultiplier tube detector (414 nm). Reduced Cu^{I}-*Ls*AA9 WT (50 μM) was first mixed with PAA (1 equiv., 50 μM), allowed to age for 1 s (**Int2** is fully formed, no **Int1**), then mixed with either buffer (as a control) or G5 (2, 4, 10 equiv.) in KPi buffer (50 mM, pH 6) at 3 ˚C. (a) The kinetic transient at 414 nm for buffer was fitted to a single exponential equation (black dashed lines) with a decay rate of 0.2 ± 0.001 s^{-1} (b)-(d) The kinetic transients at 414 nm for the G5 substrate were fitted to a double exponential equation to generate the following decay rates of **Int2**: 6.6 ± 0.3 s^{-1} and 1.5 ± 0.1 s^{-1} for G5 2 eq., 100 μM, (c) 13.7 ± 0.3 s^{-1} and 1.7 ± 0.06 s^{-1} for G5 4 eq., 200 μM and (d) 38.4 ± 0.8 s^{-1} and 2.1 ± 0.04 s^{-1} for G5 10 eq., 500 μM. (e) An overlay of the buffer data and the G5 (10 eq.) data. All fits are shown as black dashed lines.

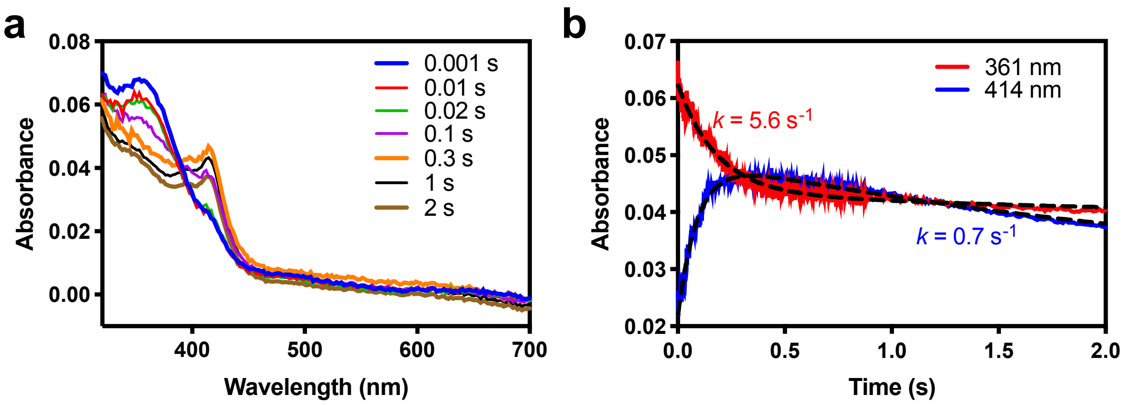

Figure S20. Double mixing stopped-flow kinetics using a photodiode array detector. Reduced Cu^{I}-*Ls*AA9 WT (200 μM, 50 μM final) was first mixed with PAA (1 equiv., 50 μM final) and held in an ageing loop for 50 ms (to allow for formation of **Int1**) before mixing with buffer (as a control) in KPi buffer (50 mM, pH 6) at 3 ˚C. (a) Raw UV-visible absorbance spectra at selected time points. (b) The kinetic transient at 361 nm was fitted to a double exponential equation (black dashed line) to generate a k_1 decay rate of 5.6 ± 0.3 s^{-1}, which correlates to the formation of **Int2** at 414 nm. The 414 nm transient was fitted to a double exponential equation (black dashed line) with a decay rate of 0.7 ± 0.07 s^{-1}.

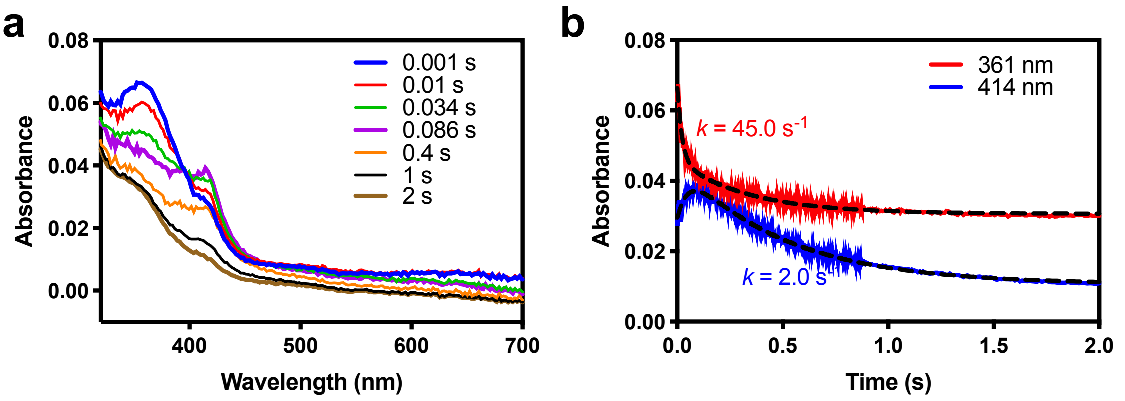

Figure S21. Double-mixing stopped-flow kinetics using a photodiode array detector. Reduced Cu^{I}-*Ls*AA9 WT (50 μM) was first mixed with PAA (1 equiv., 50 μM), allowed 50 ms ageing time (**Int1** is fully formed), then mixed with G5 substrate (1 equiv., 50 μM) in KPi buffer (50 mM, pH 6) at 3 ˚C. (a) Raw UV-visible absorbance spectra at selected time points. (b) The kinetic transient at 361 nm was fitted to a double exponential equation (black dashed line) to generate a k_1 decay rate of 45 ± 1.4 s^{-1}, which correlates to a small increase in **Int2** at 414 nm. The 414 nm transient was fitted to a double exponential equation (black dashed line) with a decay rate of 2.0 ± 0.02 s^{-1}.

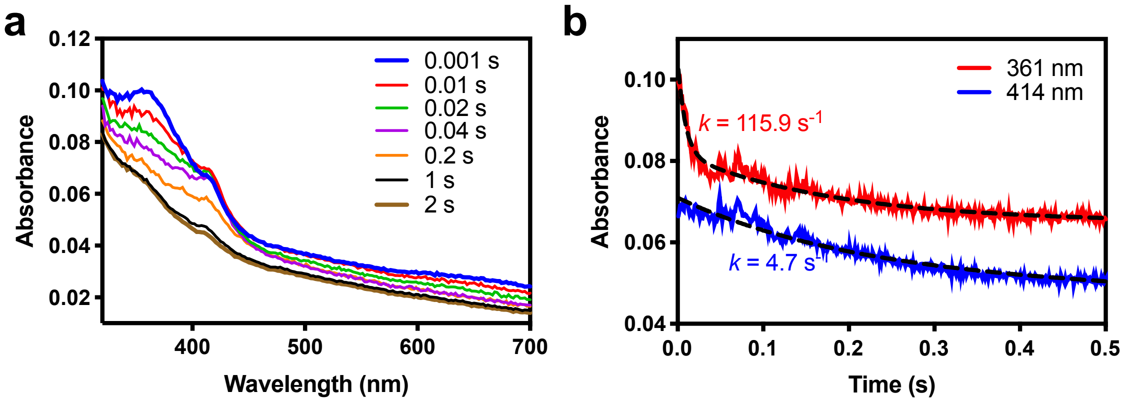

Figure S22. Double-mixing stopped-flow kinetics using a photodiode array detector. Reduced Cu^{I}-*Ls*AA9 WT (50 μM) was first mixed with PAA (1 equiv., 50 μM), allowed 50 ms aging time (**Int1** is fully formed), then mixed with G5 substrate (2 equiv., 100 μM) in KPi buffer (50 mM, pH 6) at 3 ˚C. (a) Raw UV-visible absorbance spectra at selected time points. (b) The kinetic transient at 361 nm was fitted to a double exponential equation (black dashed line) to generate a k_1 decay rate of 115.9 ± 6.1 s^{-1}. The 414 nm transient was fitted to a double exponential equation (black dashed line) with a k_1 decay rate of 4.7 ± 0.3 s^{-1}.

.

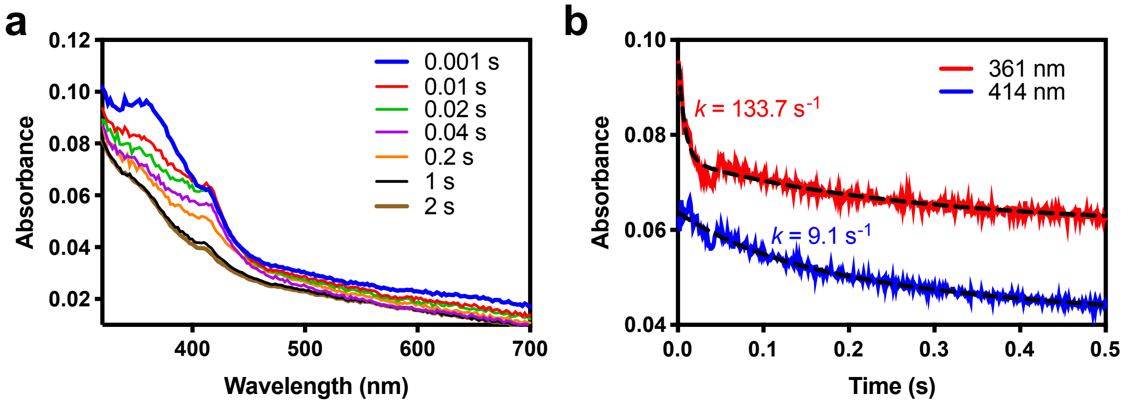

Figure S23. Double-mixing stopped-flow kinetics using a photodiode array detector. Reduced Cu^{I}-*Ls*AA9 WT (50 μM) was first mixed with PAA (1 equiv., 50 μM), allowed 50 ms ageing time (**Int1** is fully formed), then mixed with G5 substrate (4 equiv., 200 μM) in KPi buffer (50 mM, pH 6) at 3 ˚C. (a) Raw UV-visible absorbance spectra at selected time points. (b) The kinetic transient at 361 nm was fitted to a double exponential equation (black dashed line) to generate a k_1 decay rate of 133.7 ± 6.2 s^{-1}. The 414 nm transient was fitted to a double exponential equation (black dashed line) with a k_1 decay rate of 9.1 ± 0.6 s^{-1}.

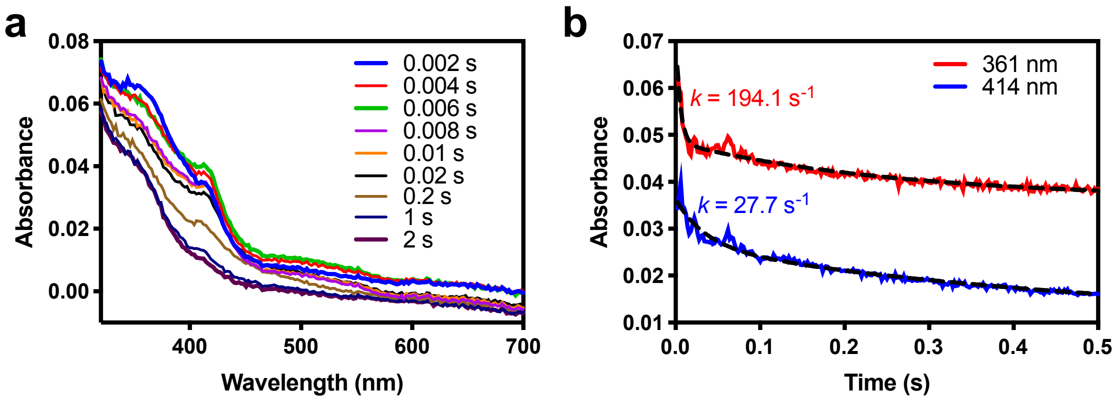

Figure S24. Double-mixing stopped-flow kinetics using a photodiode array detector. Reduced Cu^{I}-*Ls*AA9 WT (50 μM) was first mixed with PAA (1 equiv., 50 μM), allowed 50 ms ageing time (**Int1** is fully formed), then mixed with G5 substrate (10 equiv., 500 μM) in KPi buffer (50 mM, pH 6) at 3 ˚C. (a) Raw UV-visible absorbance spectra at selected time points. (b) The kinetic transient at 361 nm was fitted to a double exponential equation (black dashed line) to generate a k_1 decay rate of 194.1 ± 11.1 s^{-1}. The 414 nm transient was fitted to a double exponential equation (black dashed line) with a k_1 decay rate of 27.7 ± 1.3 s^{-1}.

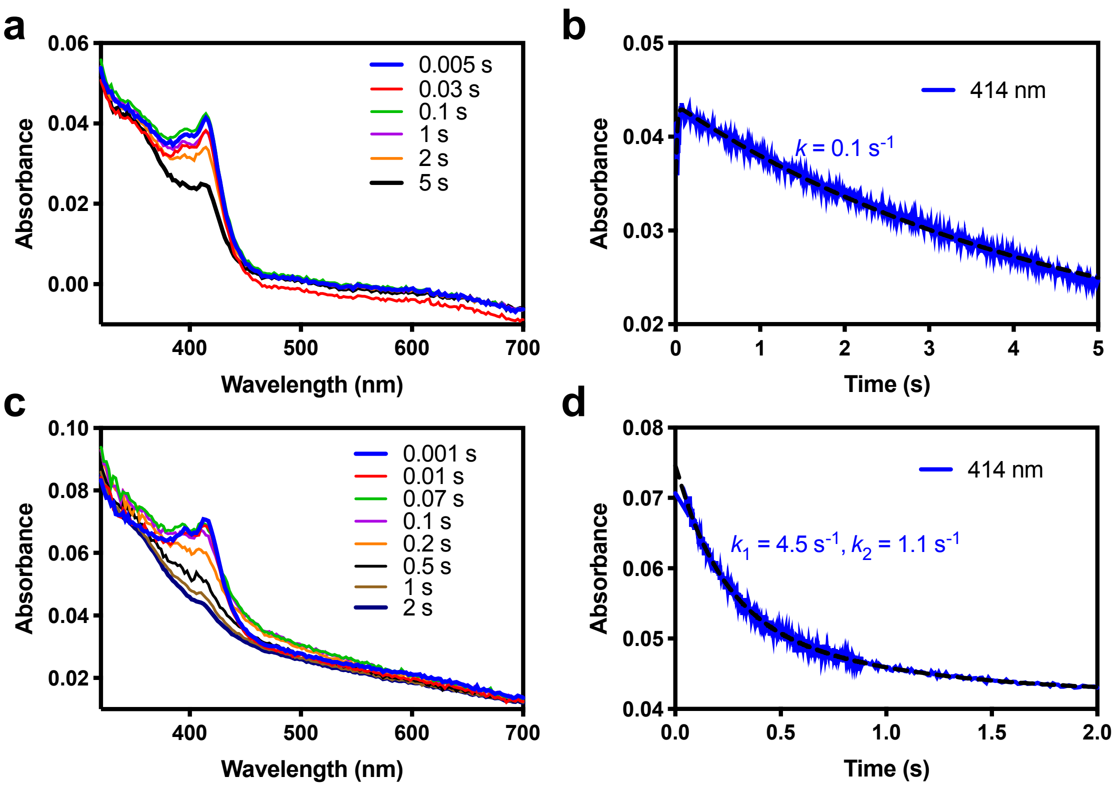

Figure S25. Double-mixing stopped-flow kinetics using a photodiode array detector. Reduced Cu^{I}-*Ls*AA9 WT (50 µM) was first mixed with PAA (1 equiv., 50 µM), allowed to age for 1 s (**Int2** is fully formed, no **Int1**) before mixing it with either buffer (as a control, a and b) or G5 substrate (2 equiv., 100 µM, c and d) in KPi buffer (50 mM, pH 6) at 3 ˚C. (a and c) Raw UV-visible absorbance spectra at selected time points. (b) The kinetic transient at 414 nm was fitted to a single exponential equation (black dashed lines) with a decay rate of 0.1 ± 0.004 s^{-1}. (d) The kinetic transient at 414 nm was fitted to a double exponential equation (black dashed lines) to generate decay rates of 4.5 ± 0.3 s^{-1} and 1.1 ± 0.2 s^{-1}.

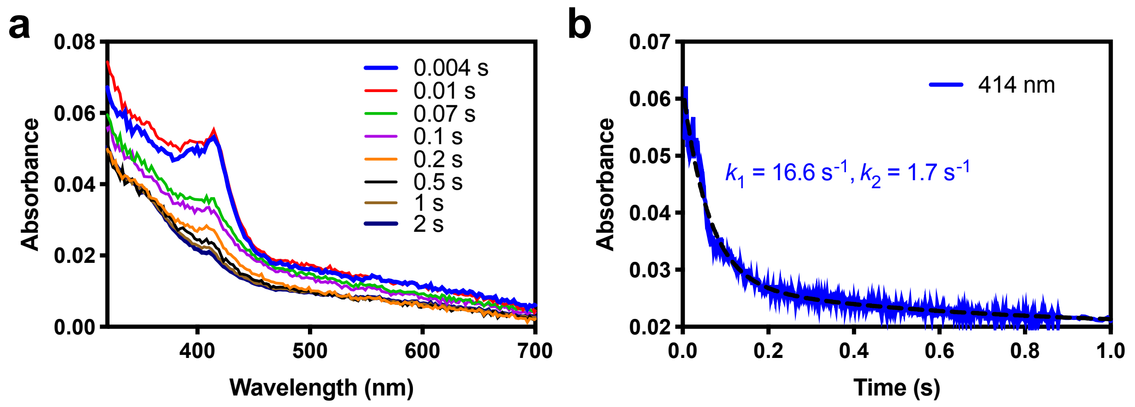

Figure S26. Double-mixing stopped-flow kinetics using a photodiode array detector. Reduced Cu^{I}-*Ls*AA9 WT (50 μM) was first mixed with PAA (1 equiv., 50 μM), allowed to age for 1 s (**Int2** is fully formed, no **Int1**), then mixed with G5 substrate (4 equiv., 200 μM) in KPi buffer (50 mM, pH 6) at 3 ˚C. (a) Raw UV-visible absorbance spectra at selected time points. (b) The kinetic transient at 414 nm was fitted to a double exponential equation (black dashed lines) to generate decay rates of 16.6 ± 0.4 s^{-1} and 1.7 ± 0.2 s^{-1}.

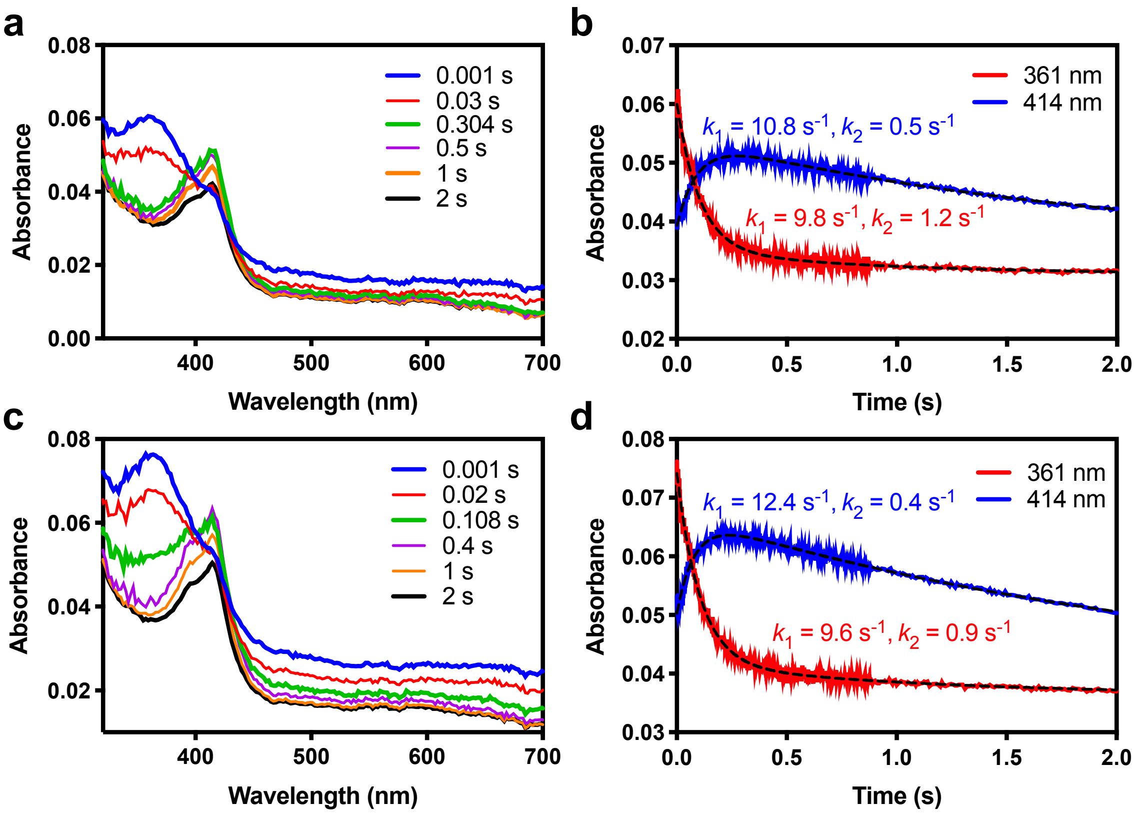

Figure S27. Double-mixing stopped-flow kinetics using a photodiode array detector. Reduced Cu^{I}-*Ls*AA9 WT (50 µM) was first mixed with *m*-CPBA (1 equiv., 50 µM), allowed 50 ms aging time (**Int1** is fully formed), then mixed with buffer (as a control, a and b) or G2 (10 equiv., 500 µM, c and d) in KPi buffer (50 mM, pH 6) at 3 ˚C. (a and c) Raw UV-visible absorbance spectra at selected time points. (b and d) The kinetic transients at 361 nm and 414 nm were fitted to a double exponential equation (black dashed lines) with decay rates of 1.2 ± 0.2 s^{-1} and 0.5 ± 0.04 s^{-1} for the buffer, and 0.9 ± 0.02 s^{-1} and 0.4 ± 0.03 s^{-1} for the G2.

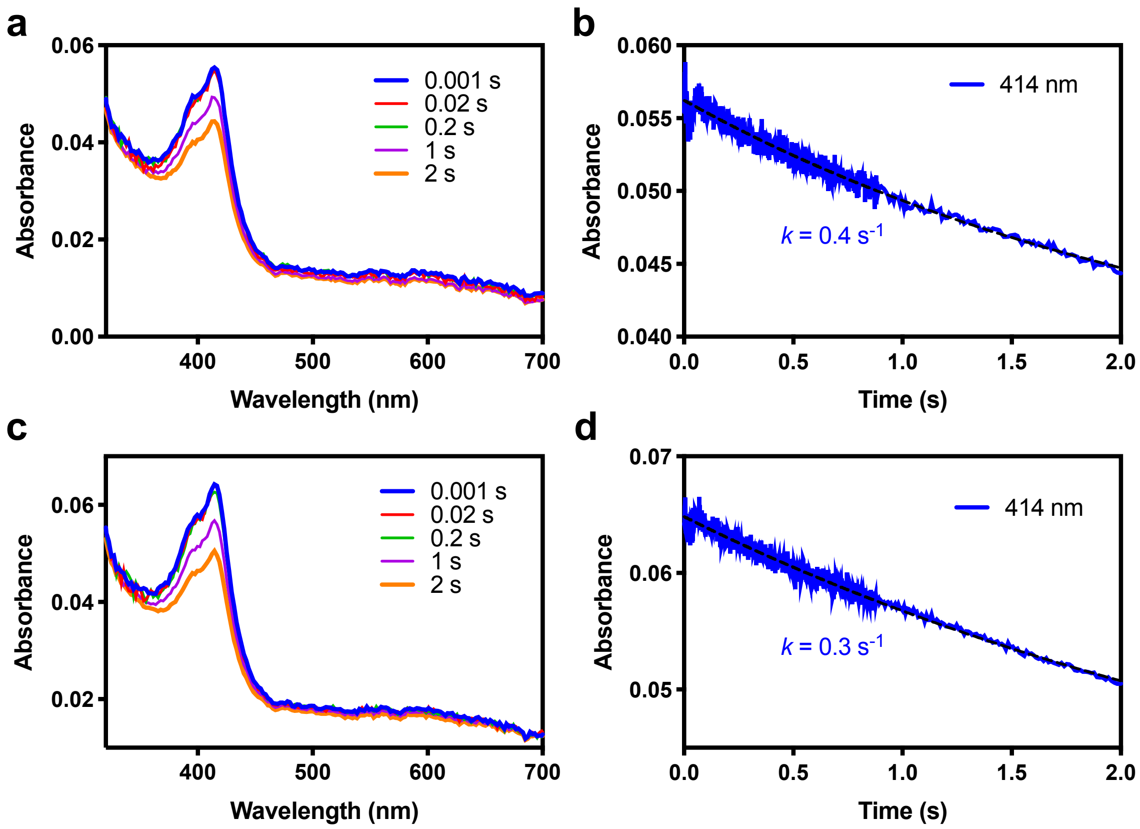

Figure S28. Double-mixing stopped-flow kinetics using a photodiode array detector. Reduced Cu^{I}-*Ls*AA9 WT (50 μM) was first mixed with *m*-CPBA (1 equiv., 50 μM), allowed 500 ms aging time (**Int2** is fully formed), then mixed with buffer (as a control, a and b) or G2 (10 equiv., 500 μM, c and d) in KPi buffer (50 mM, pH 6) at 3 ˚C. (a and c) Raw UV-visible absorbance spectra at selected time points. (b and d) The kinetic transients at 414 nm were fitted to a single exponential equation (black dashed lines) with decay rates of $0.4 \pm 0.02\ s^{-1}$ for the buffer, and $0.3 \pm 0.02\ s^{-1}$ for the G2.

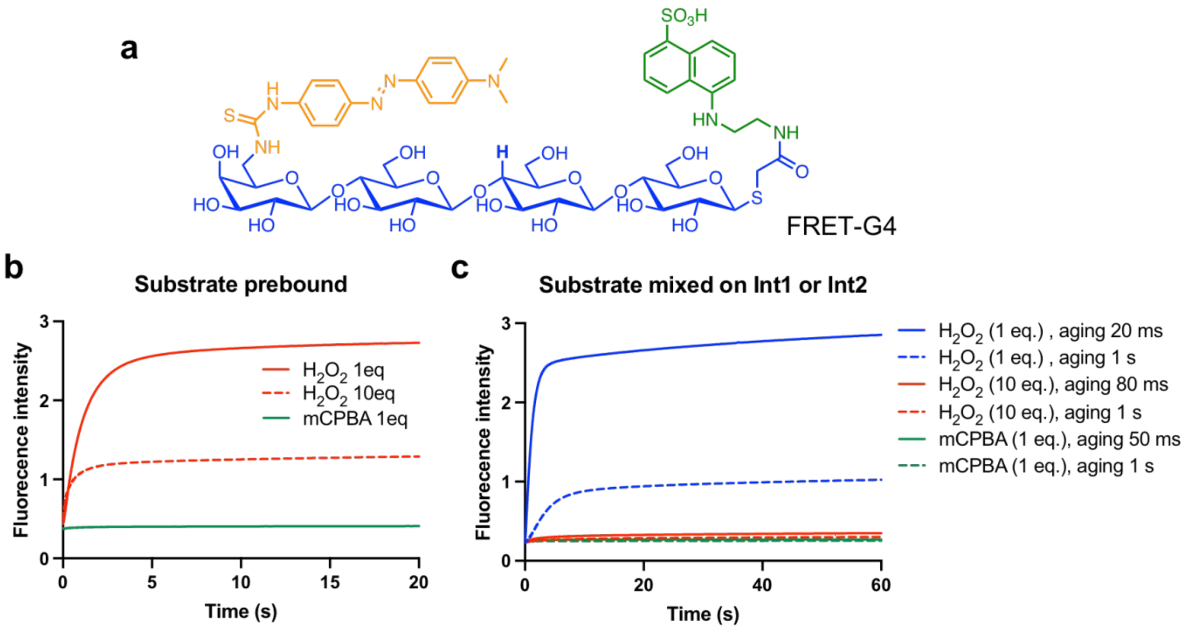

Figure S29. Determining reactivity of **Int1** and **Int2** with a cellotetraose fluorescence resonance energy transfer substrate (FRET-G4). (a) The chemical structure of FRET-G4. (b) Stopped-flow kinetics showing fluorescence upon mixing of Cu^{I}-*Ls*AA9 (50 µM), pre-bound with FRET-G4 (100 µM), with either *m*-CPBA (50 µM) or H_2O_2 (50 µM, 500 µM) in KPi buffer (50 mM, pH 6) at 3 ˚C. (c) Double mixing stopped-flow kinetics showing fluorescence changes upon mixing **Int1** or **Int2** with FRET-G4 substrate. Reduced Cu^{I}-*Ls*AA9 (50 µM) was first mixed with m-CPBA (50 µM) or H_2O_2 (50 µM, 500 µM), allowed to age for either 50 ms, 80 ms or 1 s, then mixed with the FRET-G4 substrate (100 µM). An excitation wavelength of 330 nm and a 455 nm high-pass filter was used in all stopped-flow experiments.

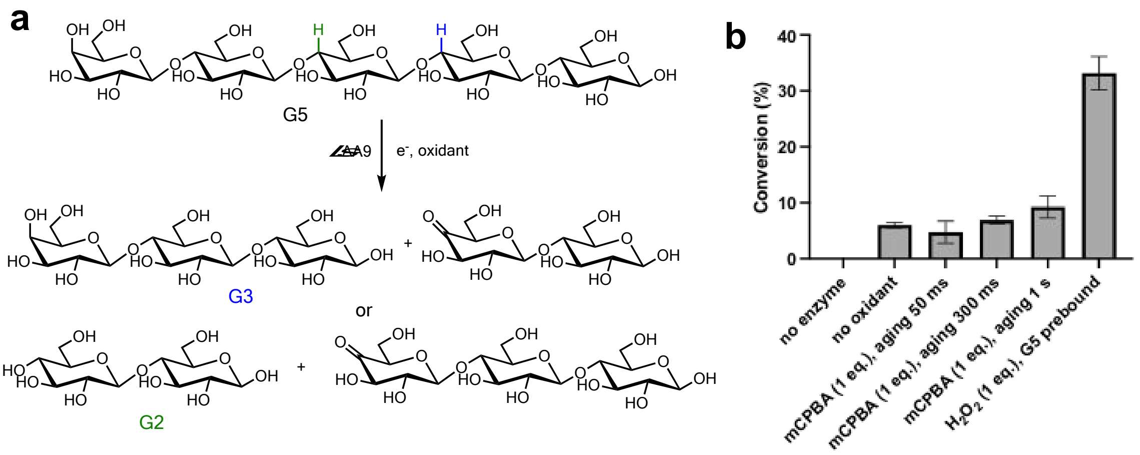

Figure S30. Determining reactivity of **Int1** and **Int2** with a soluble cellopentaose substrate (G5). (a) *Ls*AA9 catalyzes the degradation of G5 substrate, forming cellotriose (G3) and oxidized cellobiose (G2), or G2 and oxidized G3 products. (b) Product conversion by LC-MS after anaerobic double-mixing experiments using a quench-flow device. Reduced Cu^{I}-*Ls*AA9 (50 μM) was first mixed with *m*-CPBA (50 μM), allowed to age for either 50 ms, 300 ms or 1 s, then mixed with the G5 substrate (250 μM). The final reaction mixture (200 μL) was recovered from the quench-flow device, immediately quenched with 200 μL MeCN and analyzed by LC-MS. The final concentration of G2, G3 and G5 were calculated based on standard curves using reference compounds. The reaction conversion was calculated by ([G2]+[G3])/([G2]+[G3]+[G5])*100%.

Table S4. Spin-Hamiltonian Parameters used for the simulations of the EPR spectra of *Ls*AA9 variants

Samples	*Ls*AA9-WT				
	**A(^{63,65}Cu)** [a,b]	**g**-tensor[b]	a_{iso}[a]	g_{iso}	Line widths[c]
Ls-LPMO- WT	|[76 32 464]|	[2.038 2.075 2.273]	|190.6|	2.129	[4.0 0.84]
Ls-LPMO Cu^{I}+PAA[d]	|[76 32 464]	[2.038 2.075 2.273]	|190.6|	2.129	[4.0 0.84]
organic radical[d]	nd	[2.00 2.002 2.004]	nd	2.002	[0.8 1]
Ls-LPMO Y164F	|[45 58 445]|	[2.022 2.099 2.264]	|186.0|	2.128	[4.2 0.92]

[a] hyperfine and a_{iso} are given in MHz; the sign of the hyperfine coupling is not determined, so absolute values are given; nd = not determined

[b] Accurate determination of the g_x, g_y, $|A_x|$ and $|A_y|$ values were not possible owing to the second-order nature of the perpendicular region, although it was noted that satisfactory simulation could only be achieved with the particular set of values reported in the simulation. Furthermore, it was noted super-hyperfine splitting due to $^{14}N/^{1}H$-nuclei along the g_x, g_y, regions; however, given the ambiguity in the number of $^{14}N/^{1}H$ nuclei coupled to the electron spin, these were not included in the simulations to remove the over-parameterization.

[c] line widths in mT.

[d] To model the EPR spectrum of the *Ls*AA9 Cu^{I} oxidized by PAA, two EPR active species with an electron spin $S = ½$ were considered, Cu^{II} and a radical signal (Table S4). The relative population of the radical signal is < 2% compared to the Cu^{II} signal with the fastest freeze-quenching time of ~ 1 s. The population of the radical signal goes down when the freeze-quenching time is slowed down to ~2-5 s, without significant changes in the spin-Hamiltonian parameters of the Cu^{II} and the radical signals.

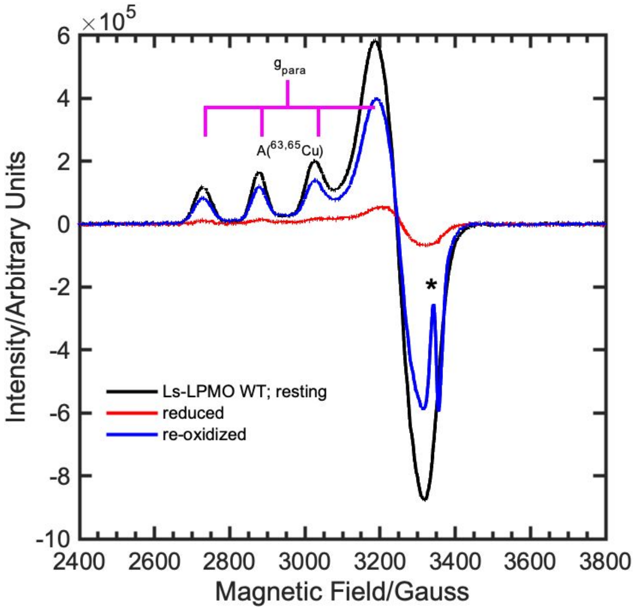

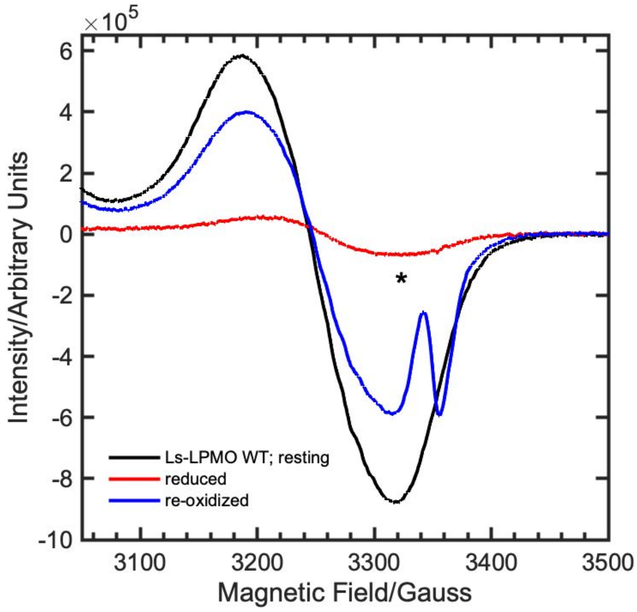

Figure S31. (top) CW-EPR spectra of *Ls*AA9 WT samples measured as a frozen solution at 20 K; the ^{63,65}Cu parallel hyperfine coupling is indicated by the magenta goal-post; Comparison of the intensity of the EPR spectra of resting and reduced LPMO enzymes indicates ~8 % Cu^{II} signal remained in the resting state. (bottom) to visualize the super-hyperfine coupling and the organic radical signal, the intense EPR signal observed between 3050-3500 G is expanded; The sharp EPR signal observed at 3350 G, indicated by the black asterisk mark is due to an organic radical with g ~ 2.002; *Conditions;* microwave power 30 dB, modulation amplitude 5 G, time constant 81 ms, conversion time 41 ms, sweep time 84 s, receiver gain 60 dB, average microwave frequency 9.386 GHz, temperature 20 K.

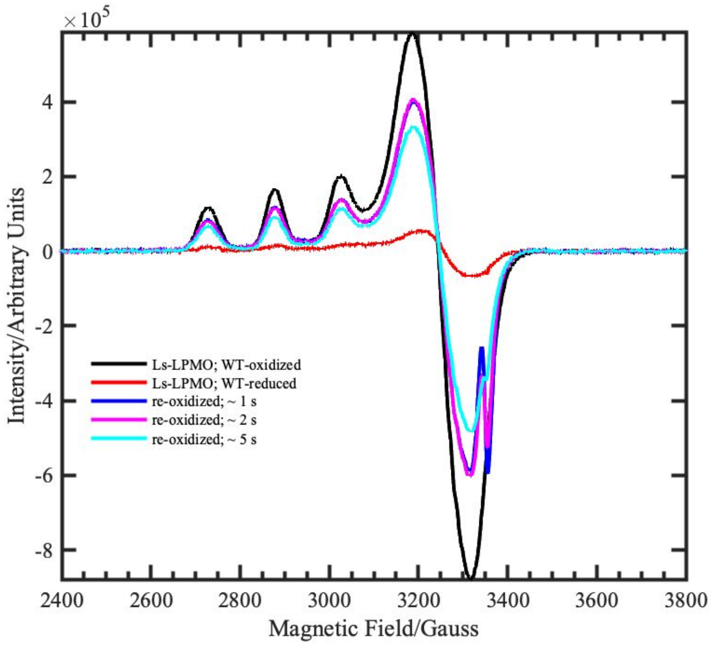

Figure S32. CW-EPR spectra of the resting (black trace)/reduced (red trace) state *Ls*AA9 WT and Cu^{I} + PAA sample freeze-quenched at various time points; measured as a frozen solution at 20 K; *Conditions;* as in Figure S31.

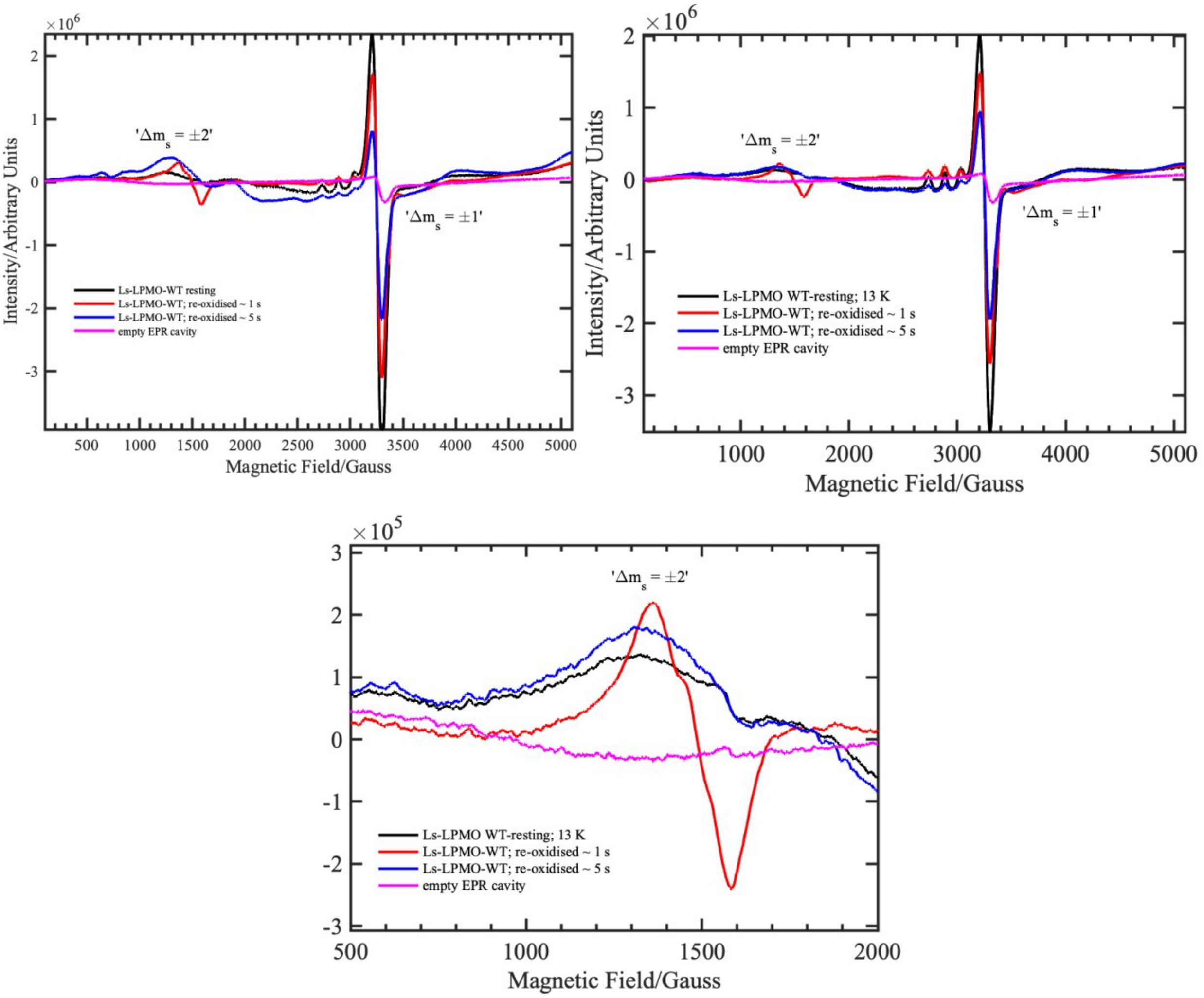

Figure S33. Low temperature, CW-EPR spectra of *Ls*AA9 WT samples measured as a frozen solution, including empty EPR cavity as a control; (top left) 8 K*,* (top right); 13 K, (bottom) 13 K -the low-field region is expanded to show the ‘$\Delta m_S = \pm 2$’ transitions observed in the *Ls*AA9 WT freeze-quenched at ~1 s (red trace), which are clearly absent in the other two samples (black trace; resting state and blue trace; *Ls*AA9 WT freeze-quenched at ~ 5 s). *Conditions;* microwave power 10 dB (20 mW), modulation amplitude 10 G, time constant 81 ms, conversion time 41 ms, sweep time 84 s, receiver gain 60 dB, average microwave frequency 9.386 GHz.

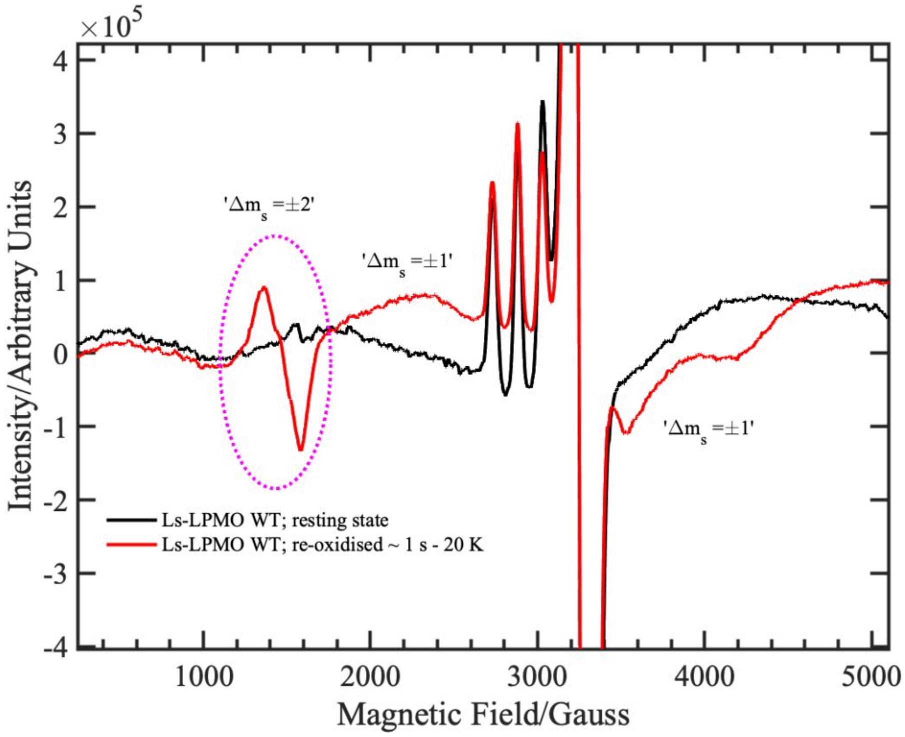

Figure S34. Low temperature CW-EPR spectra of *Ls*AA9 WT samples measured as a frozen solution at 20 K; both spectra are expanded to show both the '$\Delta m_S = \pm 2$' '$\Delta m_S = \pm 1$' transitions arise from the triplet, $S = 1$ spin state. *Conditions;* as in Figure S33.

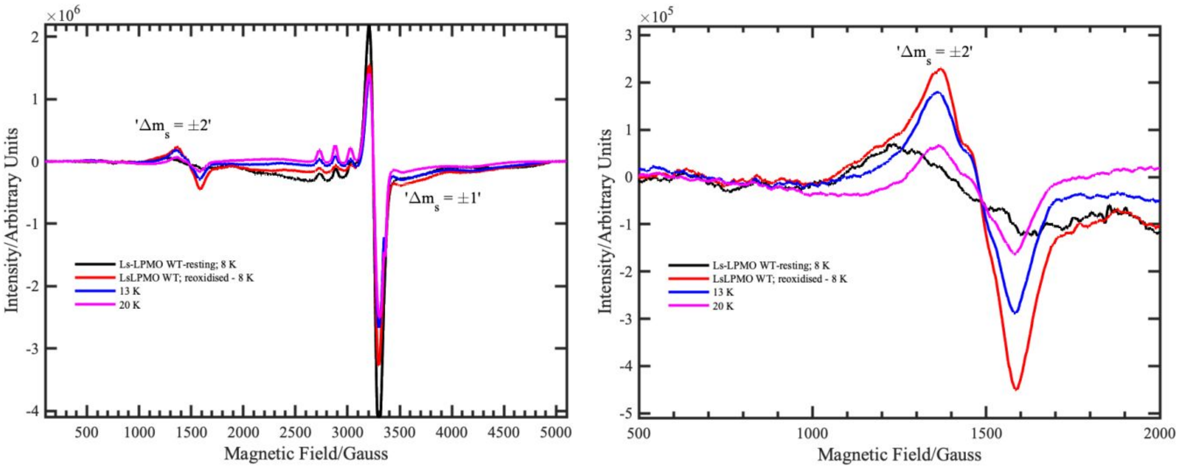

Figure S35. (left) Temperature dependent, CW-EPR spectra of *Ls*AA9 WT samples freeze-quenched at ~ 1 s, and measured as a frozen solution; (right) the low-field region is expanded to show the half-field transitions and are indicated by '$\Delta m_S = \pm 2$'; *Conditions;* as in Figure S33.

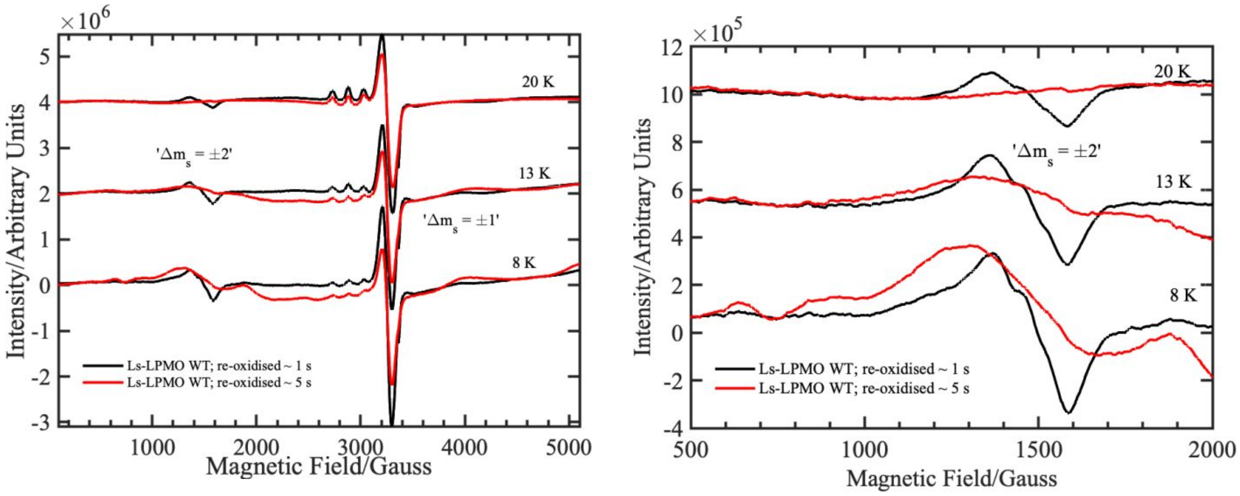

Figure S36A. (left) Temperature dependent, CW-EPR spectra of *Ls*AA9 WT samples measured as a frozen solution; (right) all the spectra are expanded between 500-2000 G to show the half-field transitions, '$\Delta m_S = \pm 2$' arise from the triplet, $S = 1$ spin state. *Conditions;* as in Figure S33.

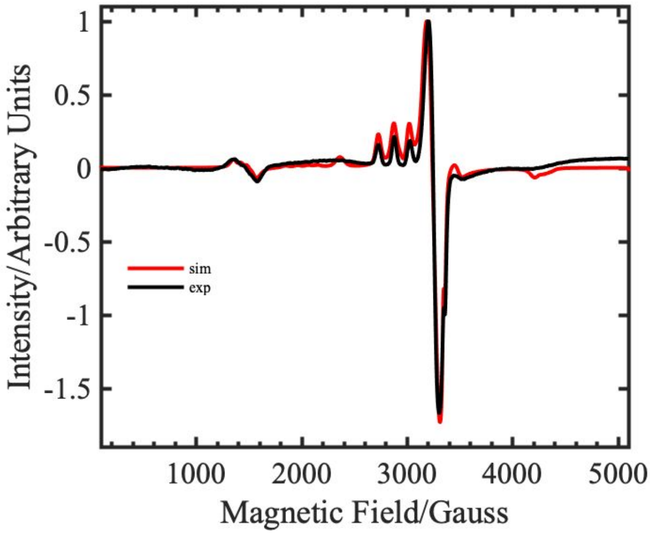

Figure S36B. Experimental and simulated spectra of *Ls*AA9 WT sample, freeze-quenched at ~ 1 s; three spins were included in the simulation – (i) resting state Cu^{II}; ~ 62 % population (ii) radical signal with ~ <0.1% population (iii) spin-coupled, Cu^{II}-radical pair; ~ 38% population; individual spin-Hamiltonian parameters of the Cu^{II} ion and radical signals were used (see Table S4 for more details) along with the near rhombic dipolar interaction tensor, **T** = [+3417 +917 -4334], and J = -100 MHz. It is noteworthy to mention that the magnitude of the exchange interaction is not sensitive to the simulated spectrum, so range of J is plausible. Also, it is noted that **T** is not axial (usually the case), which is not surprising given the large anisotropy associated with the Cu^{II} ion and the delocalisation of the electron density that often occurs within the tyrosyl radical.

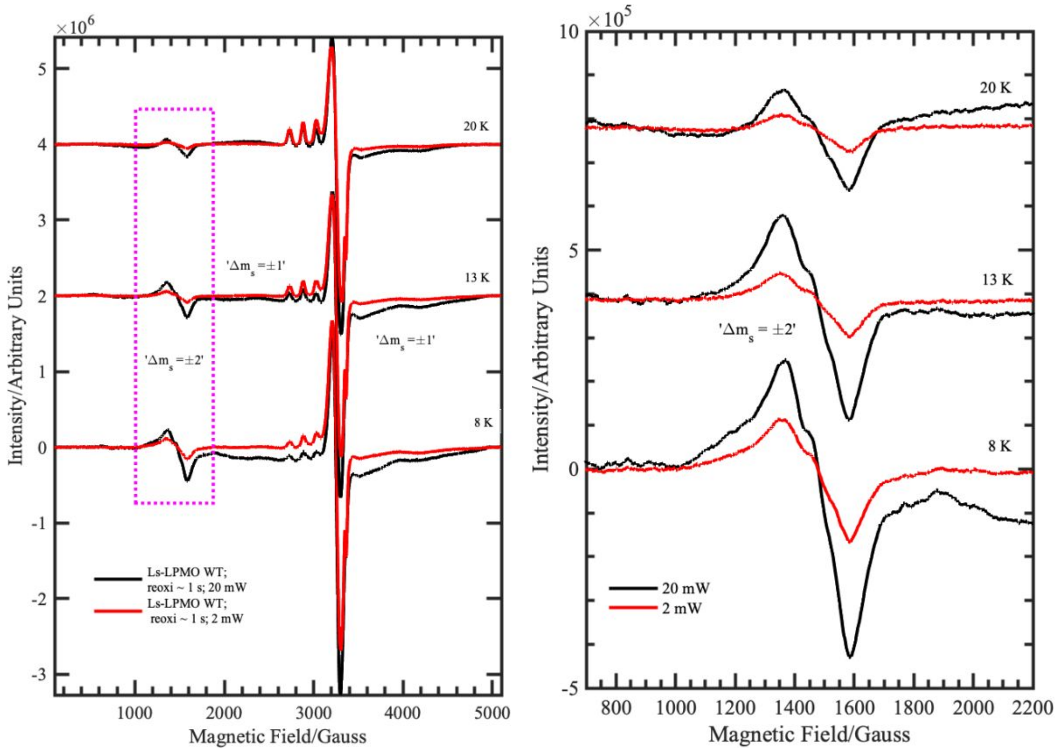

Figure S37. Temperature and microwave power dependent CW-EPR spectra of *Ls*AA9 WT freeze-quenched at ~ 1 s, measured as a frozen solution; (left) wide-sweep, CW-EPR spectra measured from 100-5100 G. (right) The spectra shown between 700-2200 G, to show the half-field transitions, '$\Delta m_S = \pm 2$' arising from the triplet, $S = 1$ spin state. *Conditions;* as in Figure S33.

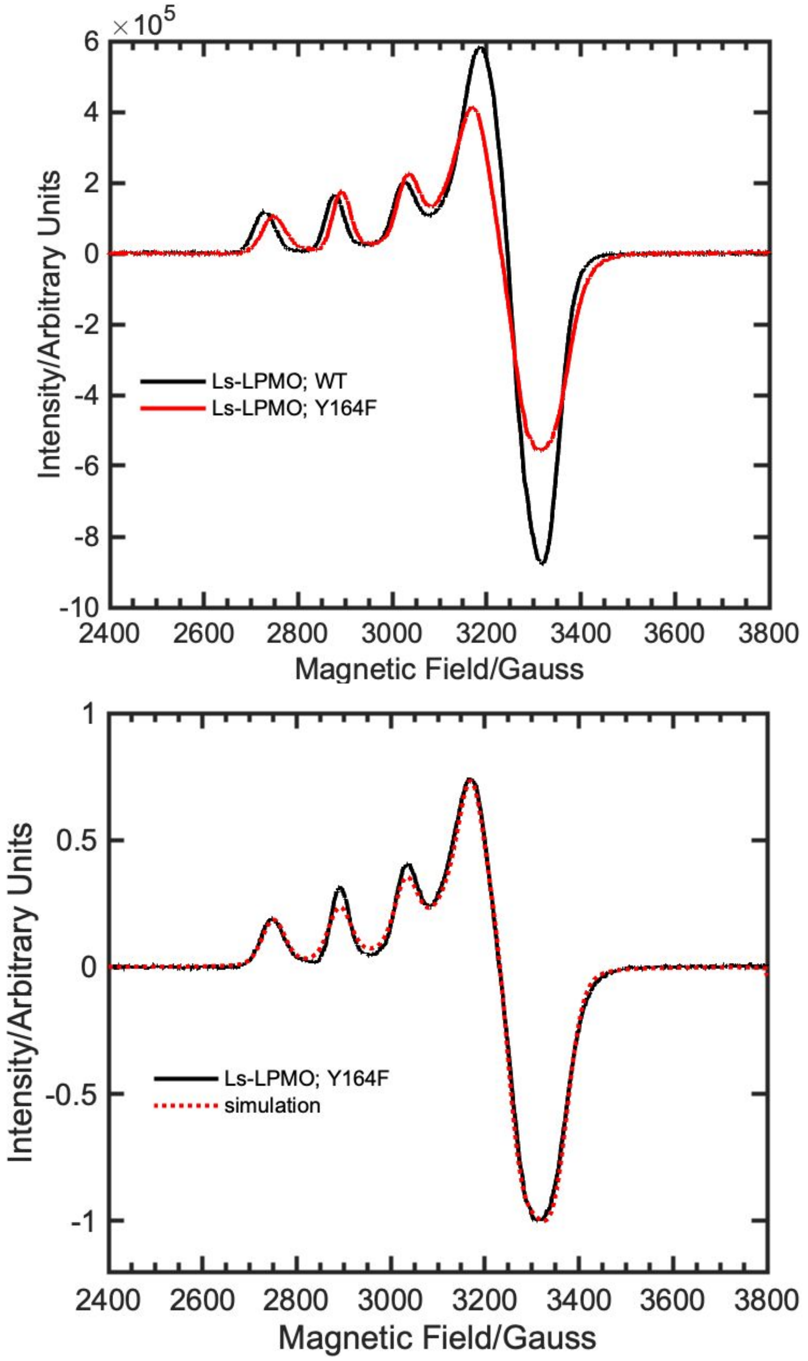

Figure S38. (top) Comparison of CW-EPR spectra of *Ls*AA9 WT (black) and *Ls*AA9 Y164F (red) samples measured as a frozen solution at 20 K. (bottom), The dotted red line (simulation) overlaid on the experimental EPR spectrum of *Ls*AA9 Y164F (black trace) is the simulation; The spin-Hamiltonian parameters used to model the EPR spectrum are given in the Table S4. *Conditions;* as in Figure S31.

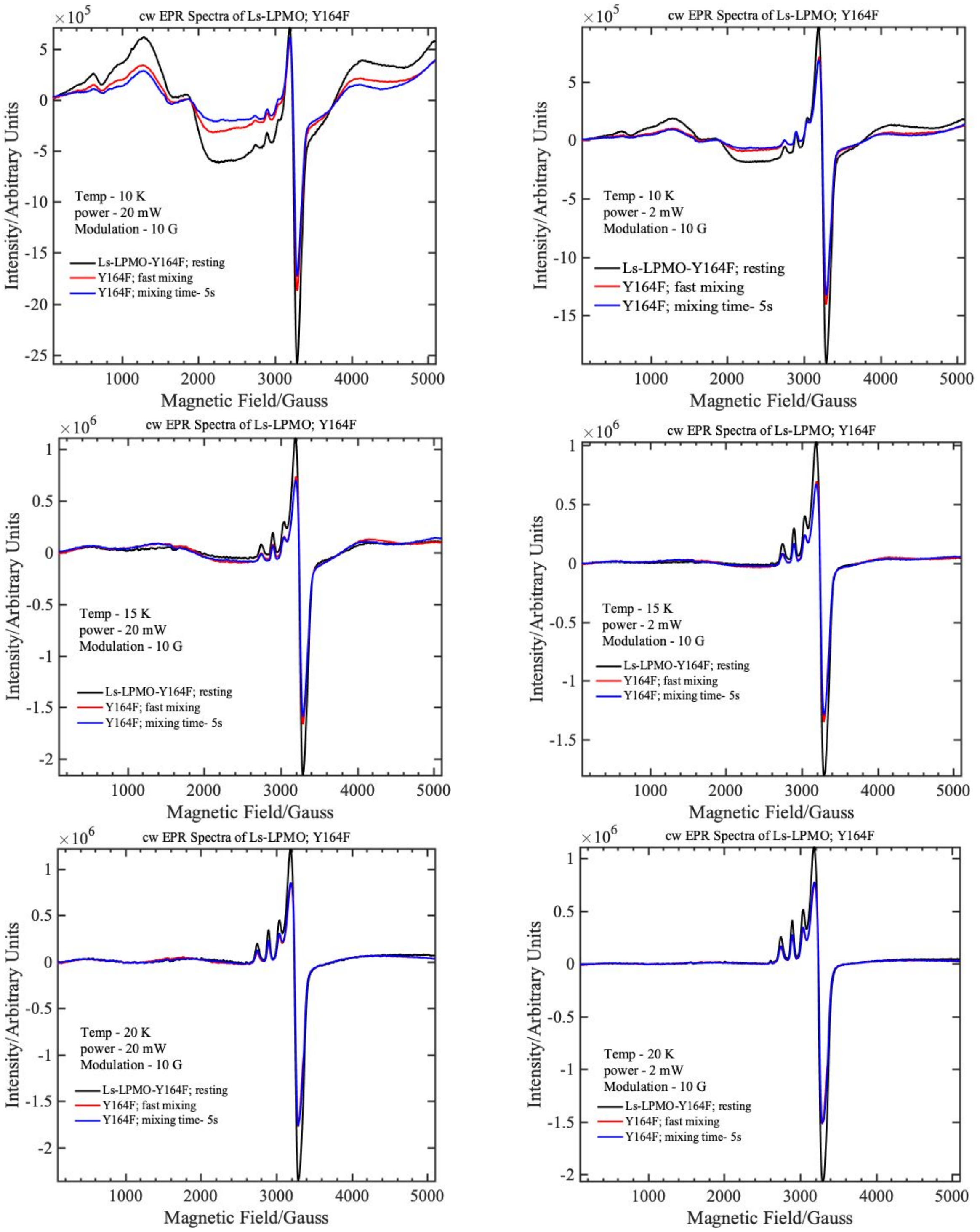

Figure S39. Temperature and microwave power dependent, CW-EPR spectra of *Ls*AA9 Y164F freeze-quenched at ~ 0.5 to1 s after mixing (fast mixing), and after 5 s, and the resting state of *Ls*AA9 Y164F, measured as frozen solutions; wide-sweep, CW-EPR spectra measured from 100-5100 G; (right). Conditions are as given in Figure S33.

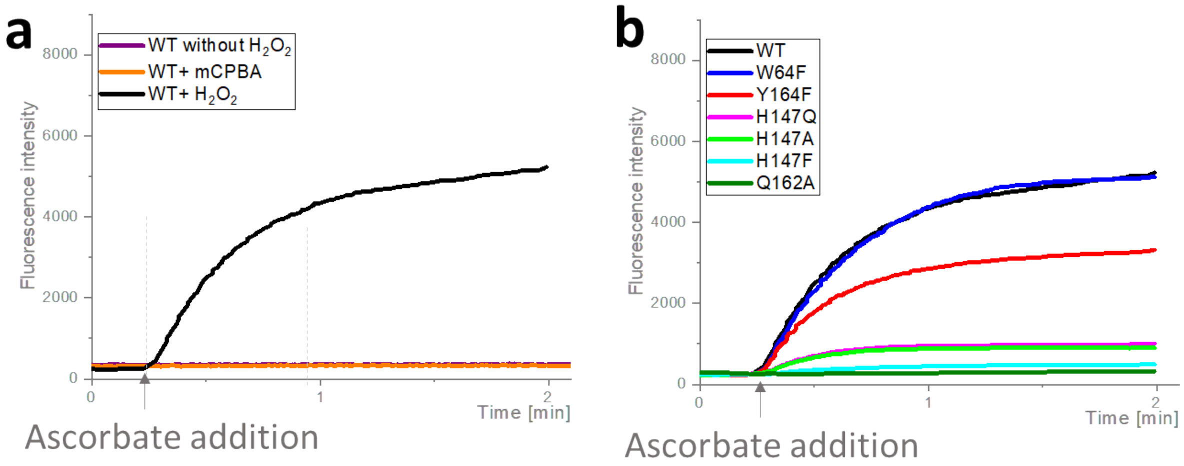

Figure S40. Steady state assays using the FRET-G4 substrate. (a) Assay of WT with different oxidants (mCPBA or H_2O_2). (b) Assay of WT and all the *Ls*AA9 variants. Reaction conditions: enzyme (1 μM), FRET-G4 substrate (20 μM), H_2O_2 (20 μM), ascorbate (2 μM) in KPi buffer (50 mM, pH 6).

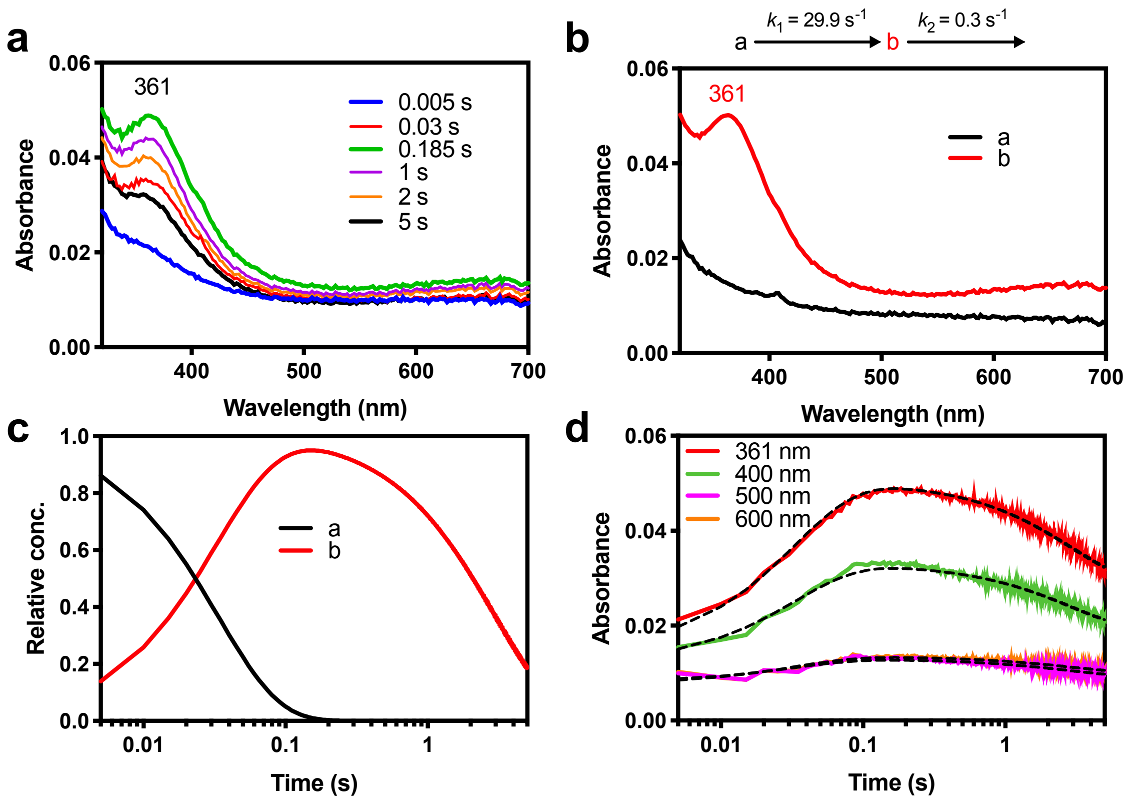

Figure S41. Stopped-flow kinetics for reoxidation of Cu^{I}-*Ls*AA9 Y164F (50 μM) mixed with PAA (1 equiv., 50 μM) in KPi buffer (50 mM, pH 6) at 3 ˚C. (a) Raw UV-visible absorbance spectra at selected time points. (b) Global fit of the raw UV-visible kinetic data using a sequential a-b-c model gives rates of $k_1 = 29.9 \pm 0.05$ s^{-1}, $k_2 = 0.3 \pm 0.001$ s^{-1}. (c) Concentration profiles of a and b from the global analysis. Species b decays to a species c with no obvious spectral features. (d) Kinetic transients at selected wavelengths, overlaid by the fits derived from the global analysis (black dashed lines).

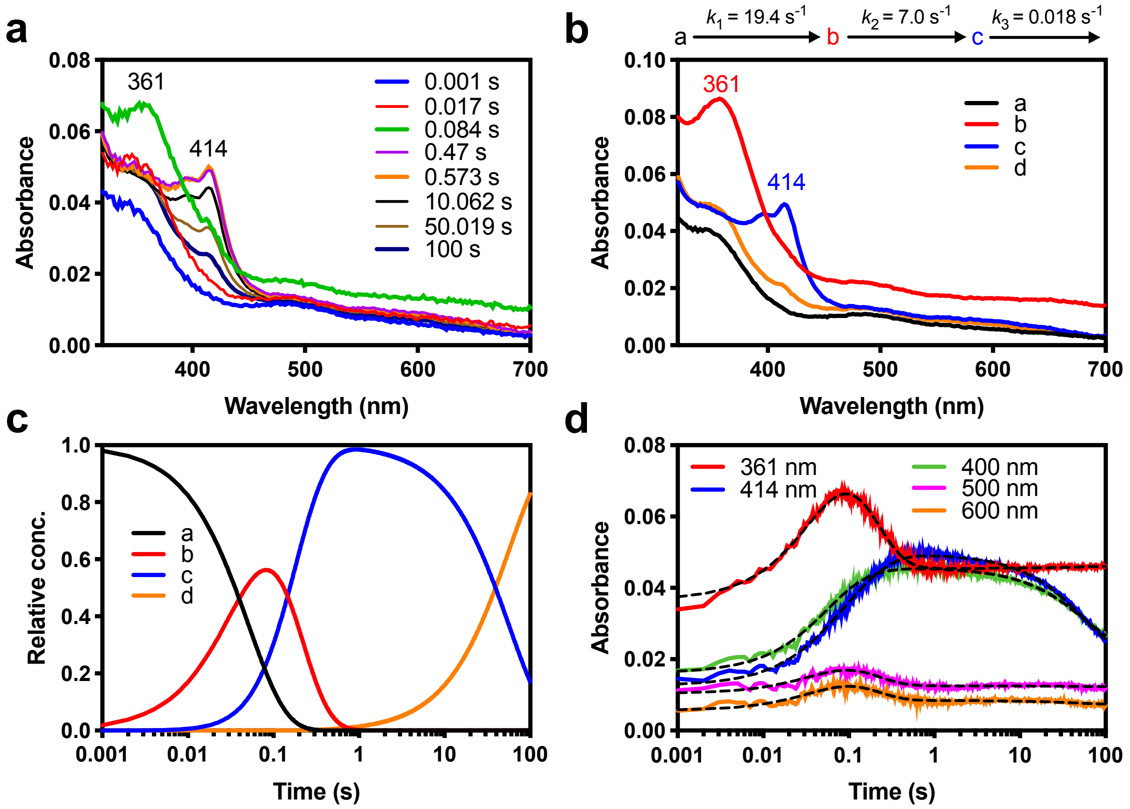

Figure S42. Stopped-flow kinetics for reoxidation of Cu^{I}-*Ls*AA9 W64F (50 μM) mixed with PAA (1 equiv., 50 μM) in KPi buffer (50 mM, pH 6) at 3 ˚C. (a) Raw UV-visible absorbance spectra at selected time points. (b) Global fit of the raw UV-visible kinetic data using a sequential a-b-c-d model gives rates of k_1 = 19.4 ± 0.04 s^{-1}, k_2 = 7.0 ± 0.01 s^{-1}, k_3 = 0.018 ± 0.001 s^{-1}. (c) Concentration profiles of a, b, c and d species from the global analysis. (d) Kinetic transients at selected wavelengths, overlaid by the fits derived from the global analysis (black dashed lines).

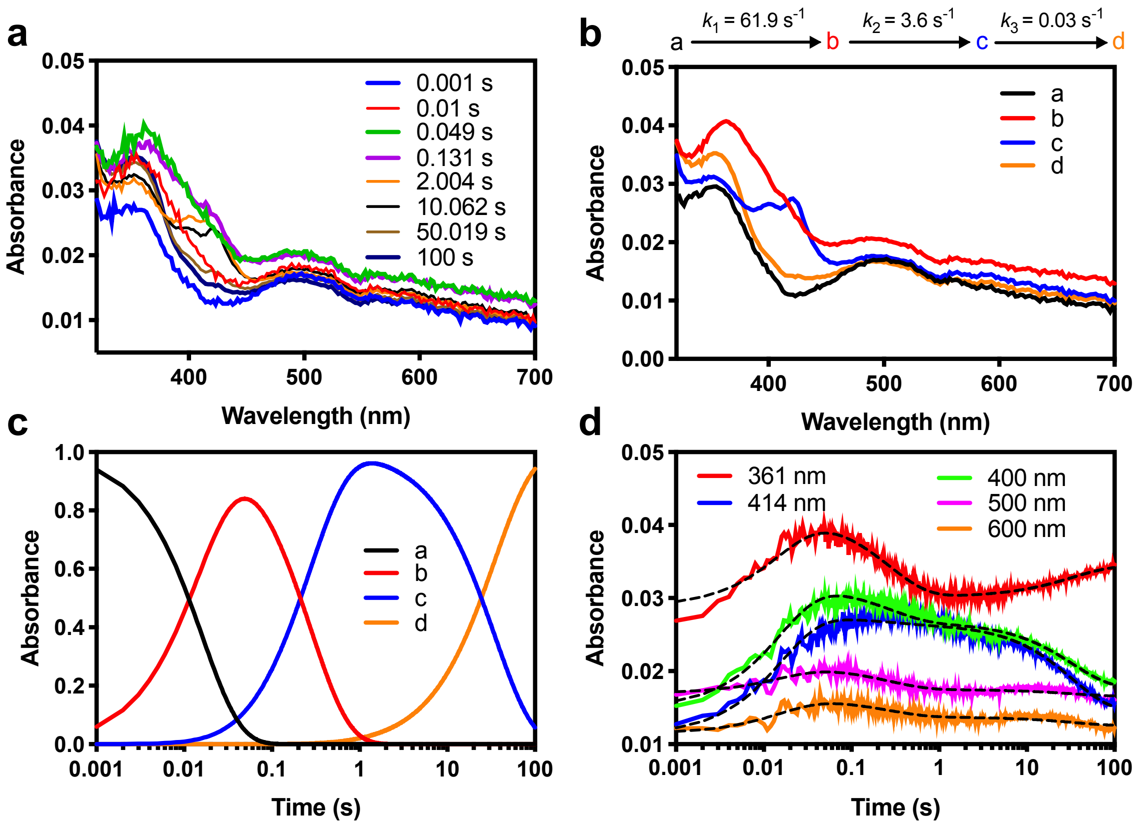

Figure S43. Stopped-flow kinetics (100 s) for reoxidation of Cu^{I}-*Ls*AA9 H147F (50 μM) mixed with *m*-CPBA (1 equiv., 50 μM) in KPi buffer (50 mM, pH 6.0) at 3 ˚C. (a) Raw UV-visible absorbance spectra at selected time points. (b) Global fit of the raw UV-visible kinetic data using a sequential a-b-c-d model gives rates of k_1 = 61.9 ± 0.2 s^{-1}, k_2 = 3.6 ± 0.01 s^{-1}, k_3 = 0.03 ± 0.001 s^{-1}. (c) Concentration profiles of a, b, c and d species from the global analysis. (d) Kinetic transients at selected wavelengths, overlaid by the fits derived from the global analysis (black dashed lines).

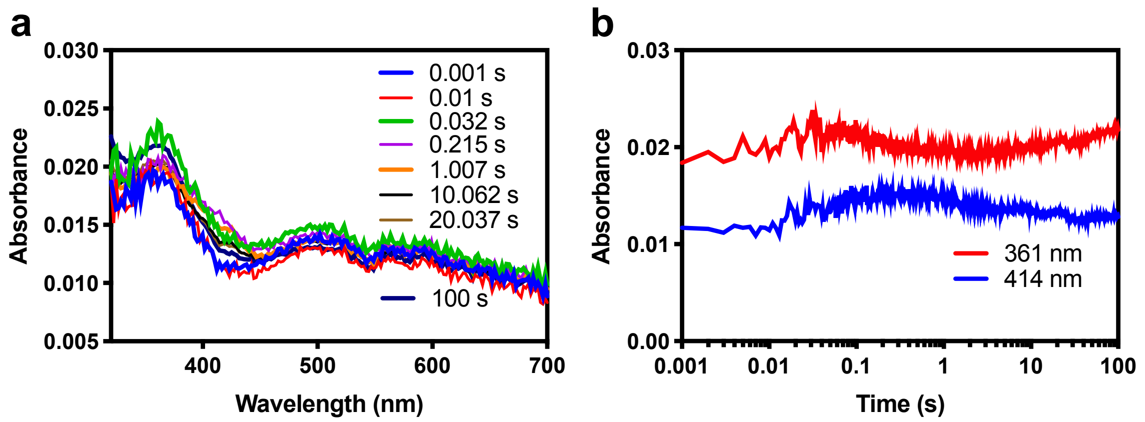

Figure S44. Stopped-flow kinetics reoxidation of Cu^{I}-*Ls*AA9 H147Q (50 µM) mixed with *m*-CPBA (1 equiv., 50 µM) in KPi buffer (50 mM, pH 6.0) at 3 ˚C. (a) Raw UV-visible absorbance spectra at selected time points. (b) Kinetic transients at selected wavelengths.

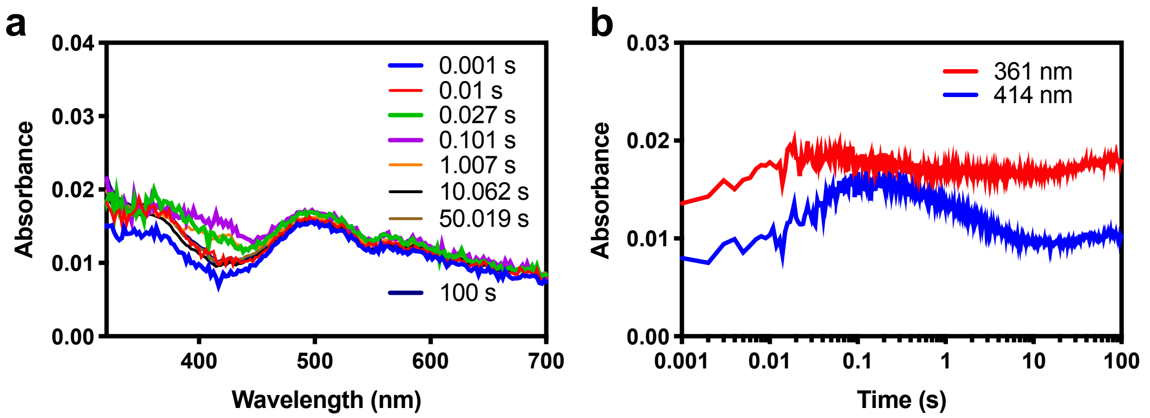

Figure S45. Stopped-flow kinetics reoxidation of Cu^{I}-*Ls*AA9 H147A (50 µM) mixed with *m*-CPBA (1 equiv., 50 µM) in KPi buffer (50 mM, pH 6.0) at 3 ˚C. (a) Raw UV-visible absorbance spectra at selected time points. (b) Kinetic transients at selected wavelengths.

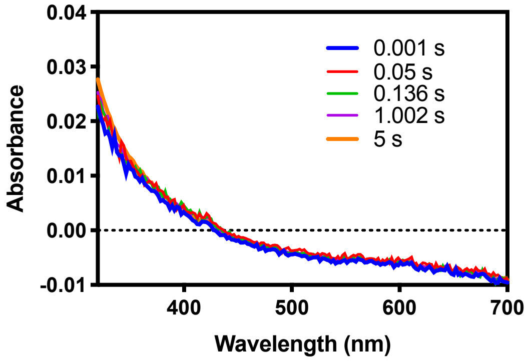

Figure S46. Stopped-flow kinetics for reoxidation of Cu^{I}-*Ls*AA9 Q162A (50 μM) mixed with H_2O_2 (1 equiv., 50 μM) in KPi buffer (50 mM, pH 6) at 3 ˚C. Raw UV-visible absorbance spectra at selected time points are shown.

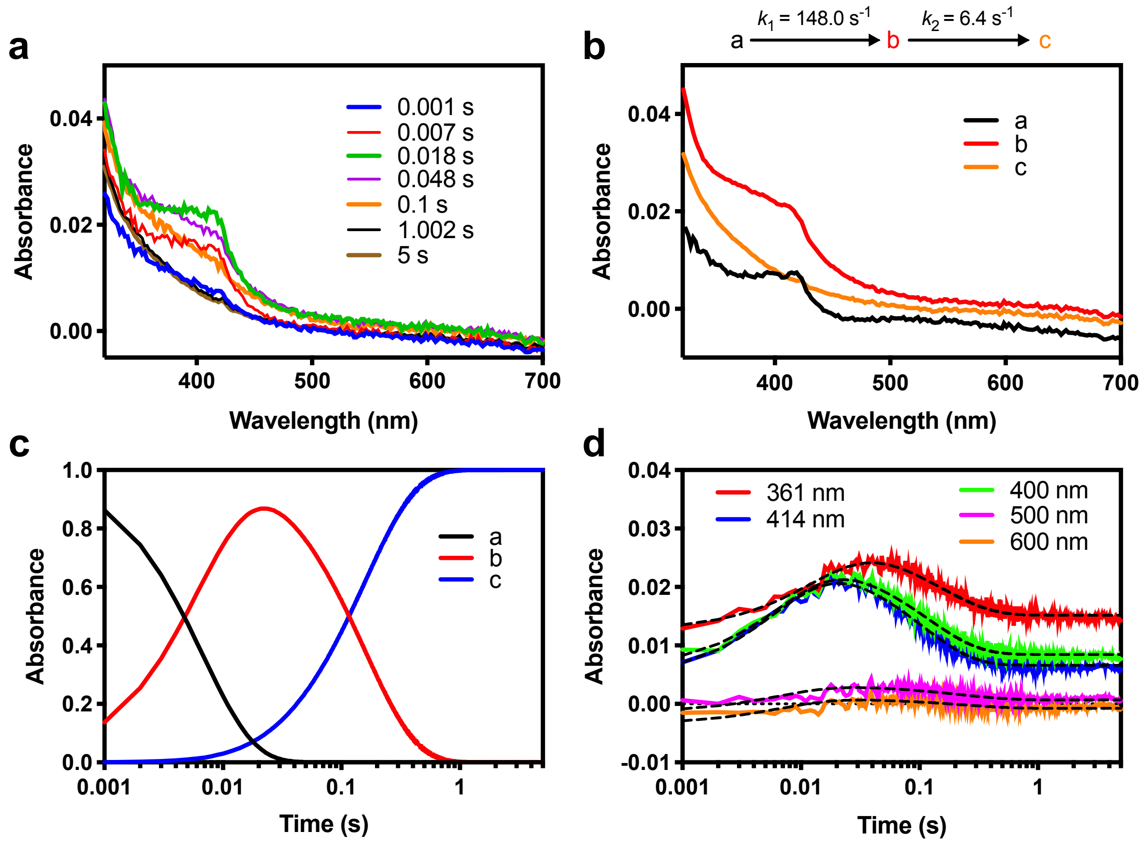

Figure S47. Stopped-flow kinetics for reoxidation of Cu^{I}-*Ls*AA9 Q162A (50 μM) mixed with *m*-CPBA (1 equiv., 50 μM) in KPi buffer (50 mM, pH 6) at 3 ˚C. (a) Raw UV-visible absorbance spectra at selected time points. (b) Global fit of the raw UV-visible kinetic data using a sequential a-b-c model gives rates of k_1 = 148.0 ± 0.01 s^{-1}, k_2 = 6.4 ± 0.01 s^{-1}. (c) Concentration profiles of a, b and c species from the global analysis. (d) Kinetic transients at selected wavelengths, overlaid by the fits derived from the global analysis (black dashed lines).

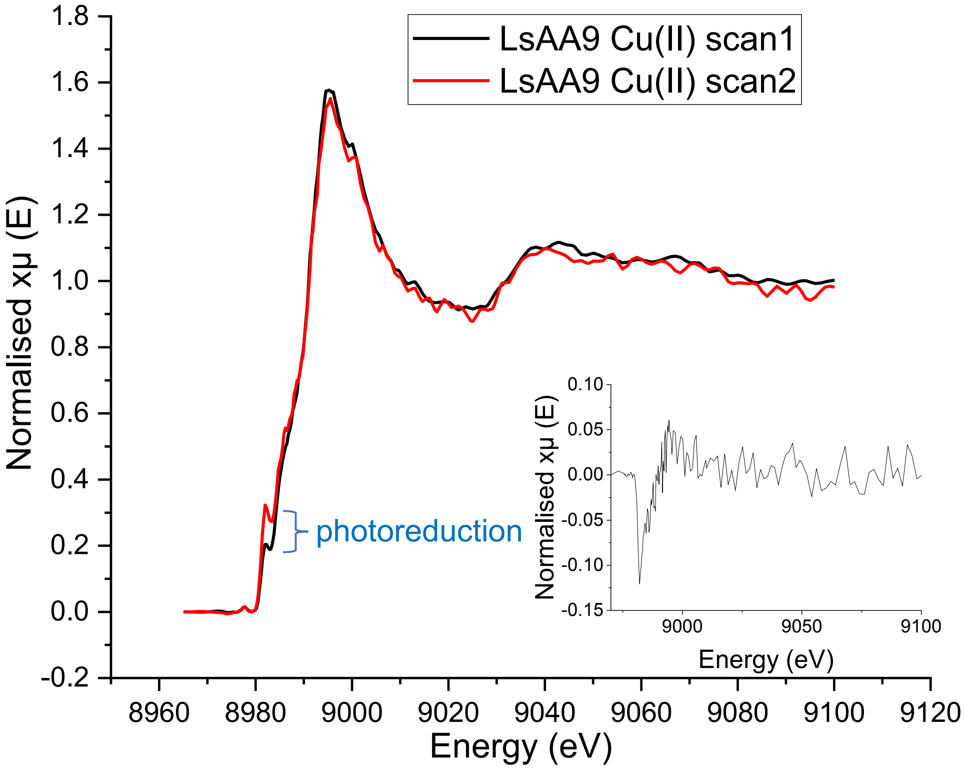

Figure S48. XAS spectra of *Ls*AA9, showing the photoreduction of the sample induced by the X-ray beam. Scan1 is the average of the first spectrum taken at three fresh positions, while scan2 is the average of the second spectrum in each of the three positions. Each spectrum has taken 6 minutes to be collected.

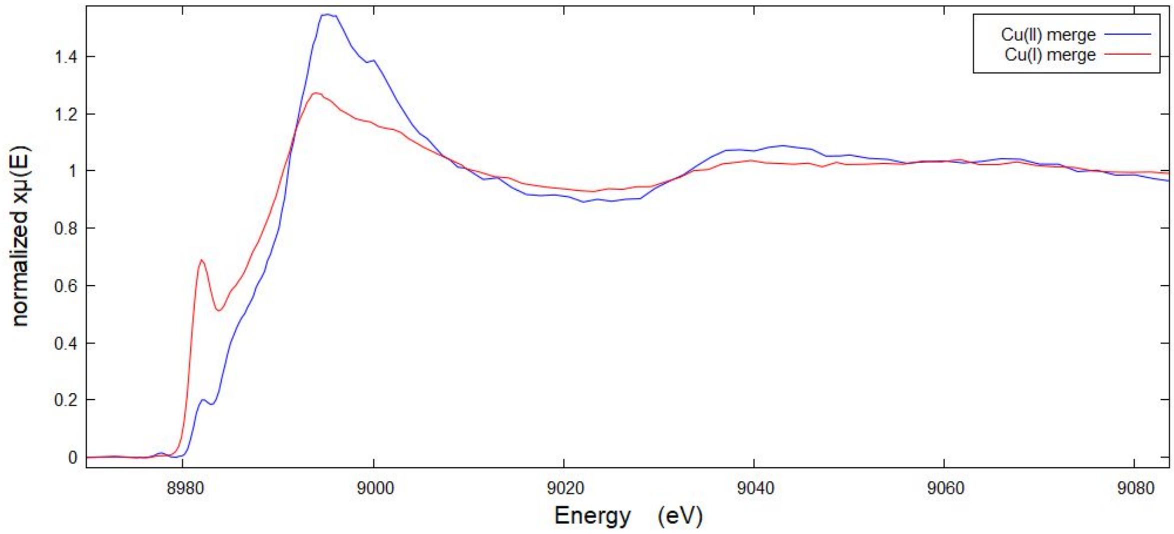

Figure S49. An overlay of HERFD-XAS spectra of Cu^{I} (red) and Cu^{II} (blue) of *Ls*AA9 Y164F controls.

Figure S50A. Int1 single scan (non-normalised) plotted with the pre-edge normalisation line.

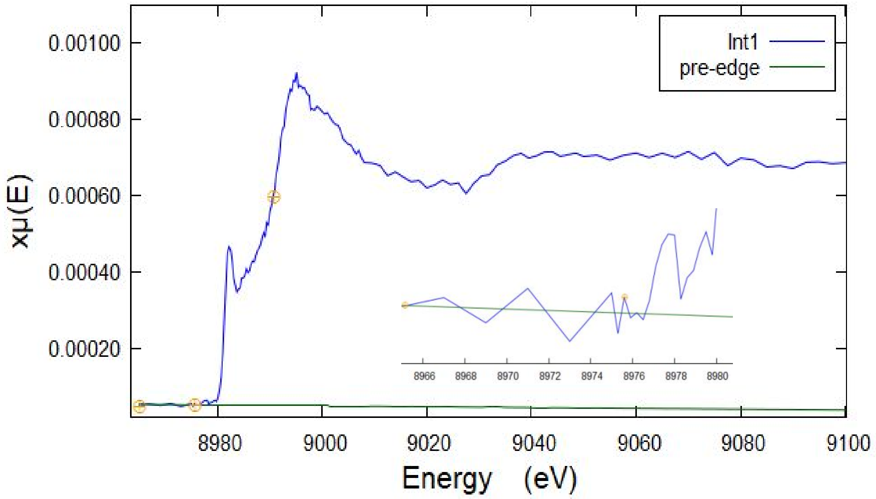

Figure S50B. Int1 average of 14 scans (blue points) with fitted 2-point average line. Asterisks show two pre-edge peaks.

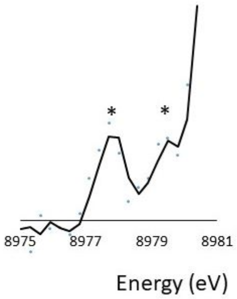

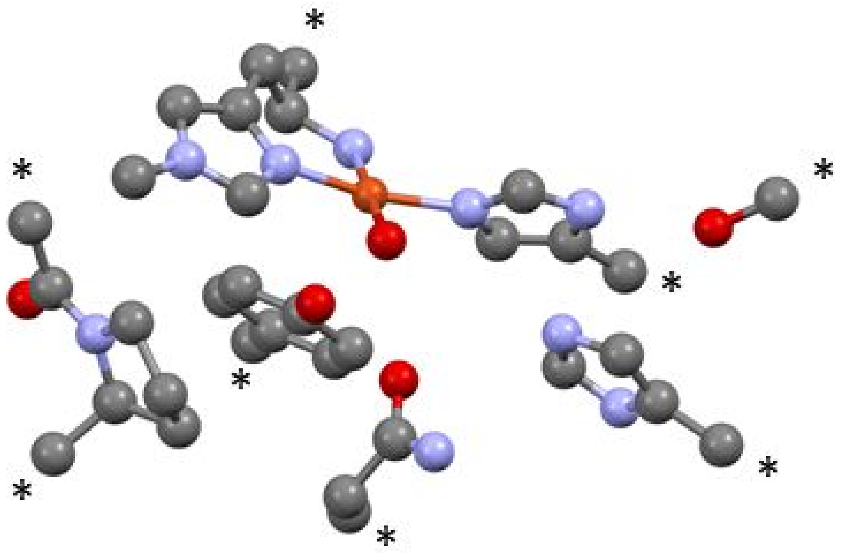

Figure S51. Structure of Cu^{II}-*Ls*AA9-histidyl radical DFT model. Hydrogen atoms are omitted for clarity and atoms kept frozen throughout the calculation are highlighted with asterisks.

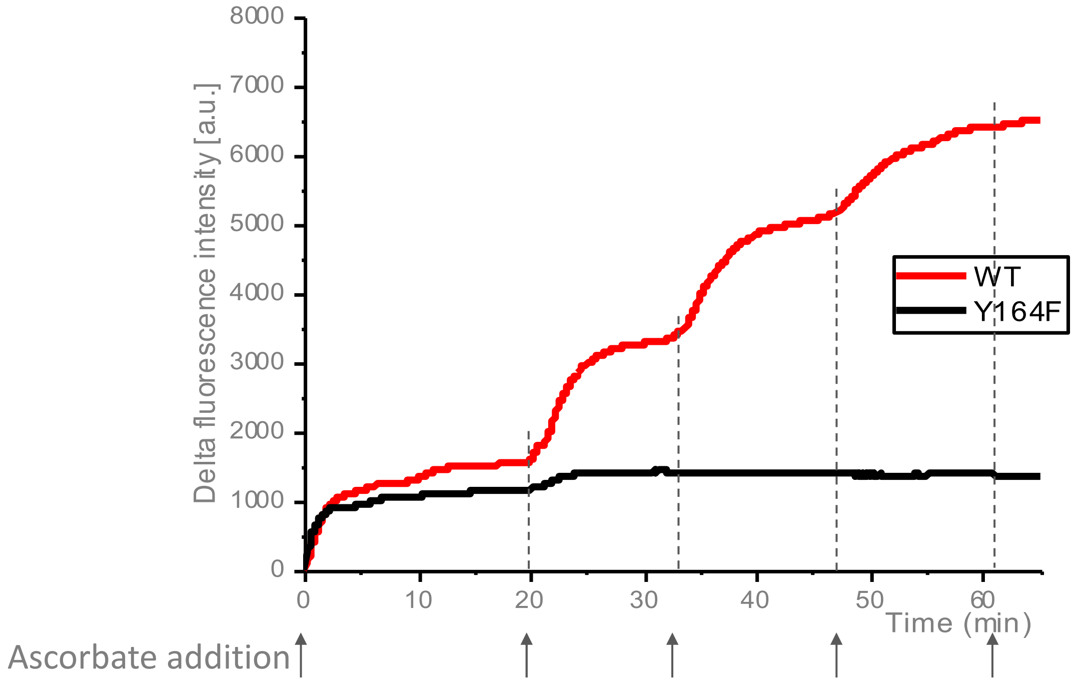

Figure S52. Steady state assays using a fluorogenic substrate show that multiple addition of low concentration of ascorbate could rescue the activity of *Ls*AA9 WT (red), but not the case for the Y164F variant (black). Reaction conditions: enzyme (0.25 µM), substrate (75 µM), H_2O_2 (50 µM), ascorbate (0.5 µM) in KPi buffer (50 mM, pH 6). Addition of ascorbate (0.5 µM) at 20 min, 33min, 48 min and 60 min are shown as vertical black arrows.

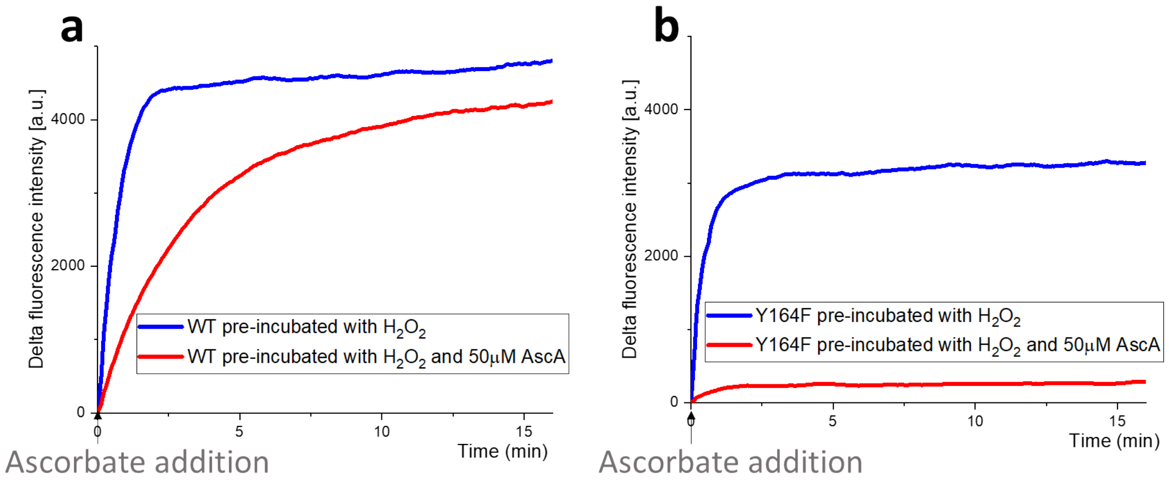

Figure S53. Steady state assays of *Ls*AA9 WT (a) and Y164F (b) which were pre-subjected to multiple cycles of uncoupled turnovers. Pre-incubation conditions: enzyme (10 μM), H_2O_2 (150 μM), ascorbate (0 μM, 50 μM, 100 μM) in KPi buffer (50 mM, pH 6). The reaction mixture above was left at room temperature for 10 min in a N_2 glovebox. Assay conditions: pre-incubated enzyme mixture (10 μL, final concentration 0.5 μM), substrate (75 μM), H_2O_2 (50 μM), ascorbate (1.5 μM) in KPi buffer (50 mM, pH 6).

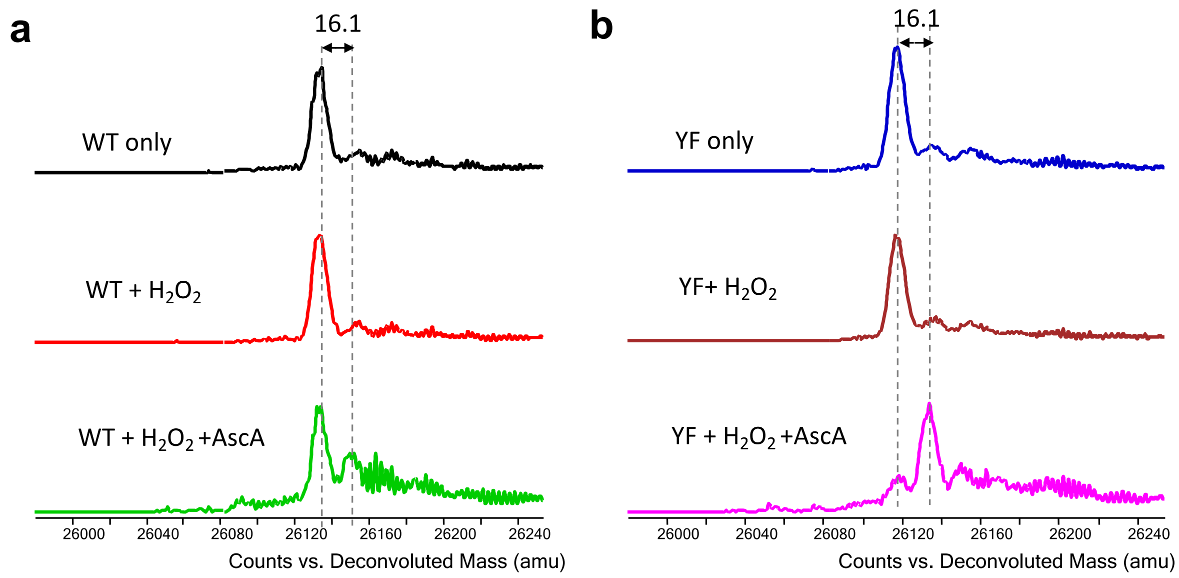

Figure S54. Intact protein mass spectrometry analysis of *Ls*AA9 WT (a) and Y164F (b) which were pre-subjected to multiple cycles of uncoupled turnovers. Pre-incubation conditions: 30 μM enzyme, 300 μM H_2O_2, with or without ascorbate (150 μM), under N_2. After two hours incubation at room temperature, the reaction mixture was desalted using spin concentrators to remove excess ascorbate.

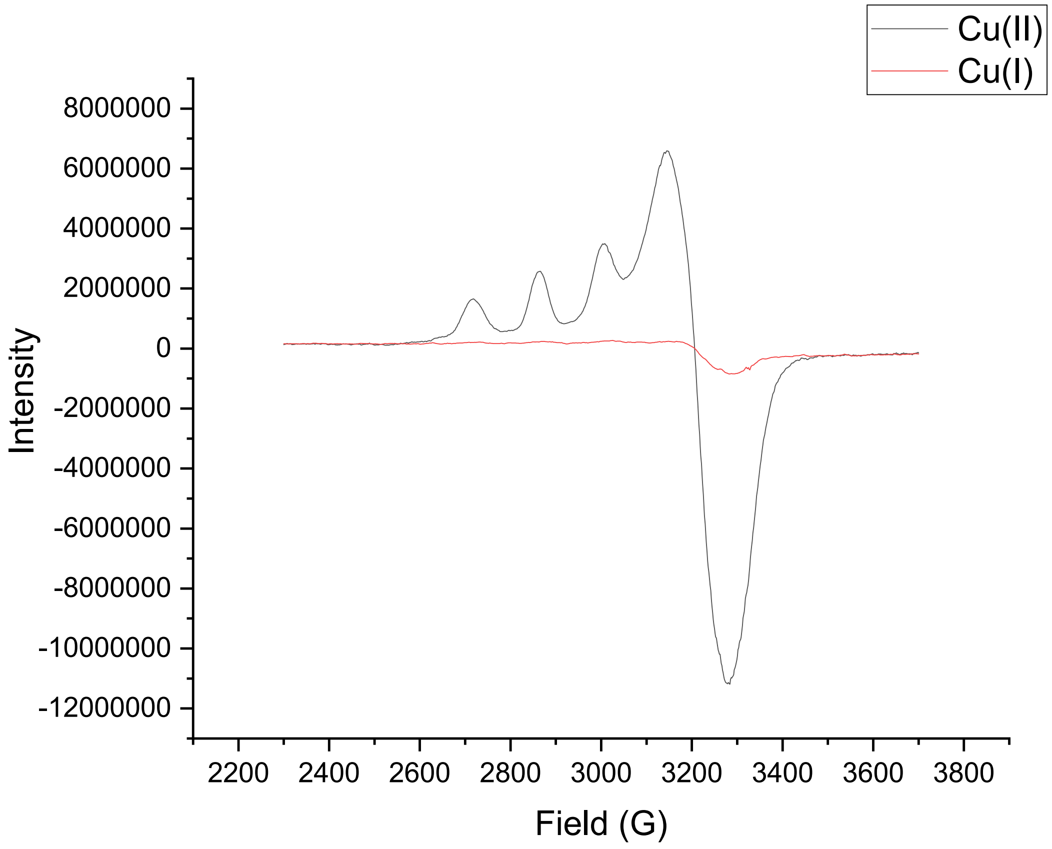

Figure S55. Reduction of Cu^{II} *Ls*Y164F (black) to form Cu^{I} *Ls*Y164F (red) with ascorbate

DFT calculations.

Atomic Coordinates for optimised Cu^{II}-*Ls*AA9-histidyl radical

N	-5.725813	-4.561189	18.039480
C	-5.449138	-4.263493	16.601596
C	-4.251984	-5.075938	16.102068
C	-5.260693	-2.748596	16.420565
C	-6.525358	-1.991217	16.647693
C	-7.154751	-1.027375	15.883879
N	-7.327826	-2.179780	17.778902
C	-8.384413	-1.409030	17.723103
N	-8.308898	-0.665860	16.586338
C	-9.287097	0.317291	16.145009
C	-9.558074	-2.687084	13.264063
C	-10.220580	-4.014763	13.605598
O	-10.188572	-4.986029	12.831465
N	-10.874951	-4.071562	14.804687
C	-11.649355	-5.270513	15.196781
C	-12.940935	-5.390955	14.380011
C	-11.885893	-5.042118	16.702217
C	-11.925034	-3.511221	16.849383
C	-10.871966	-3.030321	15.842575
C	-4.835020	-3.806054	26.828752
O	-5.561772	-4.241794	25.666583
C	-5.687000	-7.308018	23.024170
C	-5.859258	-6.030977	22.280475
C	-6.294770	-5.789748	20.985774
N	-5.651944	-4.778358	22.840415
C	-5.974785	-3.831969	21.922491
N	-6.364675	-4.421066	20.785235
C	-9.370979	-5.330981	27.072937
C	-9.005856	-4.916552	25.677919
C	-8.760733	-3.637242	25.176648
N	-8.959500	-5.838330	24.636769
C	-8.712051	-5.141161	23.535689
N	-8.594670	-3.800818	23.806441
C	-12.033968	-6.725964	19.908925
C	-12.325913	-5.284944	20.334427
C	-11.181549	-4.640281	21.091389
N	-11.492230	-3.983567	22.220706
O	-9.990473	-4.697476	20.683576
C	-8.003013	-9.808969	15.431019
C	-8.194223	-8.537170	16.234676
C	-8.224632	-7.271335	15.601955
C	-8.373382	-8.561712	17.634520
C	-8.431057	-6.091044	16.329746
C	-8.578818	-7.385314	18.381753
C	-8.616317	-6.136567	17.728559
O	-8.821142	-4.947212	18.382584
Cu	-6.980819	-3.443992	19.196811
H	-8.821101	-2.388002	14.039586
H	-11.035669	-5.455521	17.276700
H	-12.808846	-5.543010	17.054479
H	-11.693865	-3.160853	17.873725
H	-12.926348	-3.117381	16.577272
H	-9.872856	-2.958651	16.329585
H	-11.115330	-2.035146	15.422017
H	-11.018957	-6.164977	15.015742
H	-4.979704	-4.495829	27.689253
H	-5.123746	-2.777831	27.139633
H	-6.523567	-4.249717	25.872504
H	-9.281808	-4.484998	27.781275
H	-8.722751	-6.158646	27.426712
H	-8.735679	-2.650428	25.651111
H	-8.345225	-3.070670	23.130661
H	-8.629914	-5.534198	22.516115

H	-11.848452	-7.375589	20.787576
H	-11.148478	-6.780951	19.246919
H	-13.252138	-5.230559	20.940543
H	-12.499432	-4.651289	19.437727
H	-10.753612	-3.542752	22.776096
H	-7.939192	-10.697061	16.089169
H	-7.077221	-9.765997	14.820080
H	-8.111206	-7.202419	14.508245
H	-8.358400	-9.528550	18.162904
H	-8.499435	-5.120201	15.818728
H	-8.733887	-7.439847	19.470421
H	-4.839658	-4.585604	18.558594
H	-6.359260	-4.570983	16.050709
H	-4.098703	-4.903751	15.018406
H	-4.455574	-2.401400	17.107772
H	-4.903149	-2.551235	15.391597
H	-6.878677	-0.569545	14.927832
H	-10.124900	0.325487	16.864295
H	-8.833457	1.327254	16.100275
H	-9.671706	0.057017	15.139127
H	-4.678384	-7.383553	23.478552
H	-5.829805	-8.167168	22.343639
H	-6.579874	-6.510059	20.213565
H	-5.416677	-4.581794	23.840951
H	-5.922223	-2.753284	22.094184
H	-3.323233	-4.778760	16.632440
H	-4.414993	-6.161329	16.258561
H	-6.124706	-5.506531	18.108363
H	-3.760619	-3.800670	26.561813
H	-6.431996	-7.386099	23.844592
H	-10.416740	-5.701931	27.106158
H	-12.447618	-3.962645	22.579304
H	-12.894316	-7.141445	19.349301
H	-12.703782	-5.445750	13.300194
H	-13.489698	-6.313366	14.662171
H	-13.608559	-4.521990	14.554587
H	-9.043585	-2.790066	12.292416
H	-10.311993	-1.875850	13.193342
O	-8.165841	-2.464413	20.284562
H	-8.787553	-3.164274	20.603712
H	-8.845888	-9.966666	14.725086
H	-9.327181	-5.046126	19.255463

ORCA input file for Cu^{II}-*Ls*AA9-histidyl radical

```
#LsAA9, CuII OH, His1radical, broken symmetry, BP86, Geom Opt, Hydroph. env.,

! UKS BP86 RI D3BJ Def2-SVP def2/J TIGHTSCF CPCM Opt SlowConv
%pal nprocs 16
 end

%cpcm
  epsilon 4.0
  refrac 1.33
 end

%scf
 MaxIter 1000
 Brokensym 1,1 # The number of electrons on each site
end

%geom

Constraints
{C 2 C} # Constraining atom no. 3 in space (GaussView)
{C 10 C}
```

```
{C 15 C}
{C 38 C}
{C 33 C}
{C 27 C}
{C 21 C}
{C 19 C}

 end
end

*xyz 1 1
N       -5.698919000     -4.510221000     18.048105000 newgto "def2-TZVP" end
C       -5.435136000     -4.247641000     16.602019000
C       -4.251984000     -5.075938000     16.102068000
C       -5.227210000     -2.741894000     16.370313000
C       -6.479302000     -1.948087000     16.555732000
C       -7.082578000     -1.011354000     15.731115000
N       -7.262422000     -2.081648000     17.691349000 newgto "def2-TZVP" end
C       -8.303449000     -1.254790000     17.570715000 newgto "def2-TZVP" end
N       -8.226283000     -0.581638000     16.392328000
C       -9.180429000      0.405272000     15.899827000
C       -9.558074000     -2.687084000     13.264063000
C      -10.234495000     -4.010355000     13.597394000
O      -10.227175000     -4.970836000     12.810819000
N      -10.873807000     -4.070463000     14.804274000
C      -11.652204000     -5.266713000     15.199278000
C      -12.940935000     -5.390955000     14.380011000
C      -11.889849000     -5.034749000     16.704611000
C      -11.888298000     -3.505158000     16.862164000
C      -10.819867000     -3.049916000     15.860100000
C       -4.835020000     -3.806054000     26.828752000
O       -5.565702000     -4.272737000     25.683312000
C       -5.687000000     -7.308018000     23.024170000
C       -5.926528000     -6.026549000     22.295832000
C       -6.369233000     -5.771781000     21.004400000
N       -5.737777000     -4.776601000     22.868352000
C       -6.069466000     -3.826816000     21.956744000
N       -6.454966000     -4.399409000     20.811522000 newgto "def2-TZVP" end
C       -9.370979000     -5.330981000     27.072937000
C       -9.099354000     -4.860630000     25.691220000
C       -8.692984000     -3.598247000     25.257729000
N       -9.289028000     -5.696036000     24.606055000
C       -8.996256000     -4.952040000     23.534252000
N       -8.640422000     -3.675909000     23.877621000
C      -12.033968000     -6.725964000     19.908925000
C      -12.445135000     -5.311846000     20.317891000
C      -11.309165000     -4.524866000     20.939311000
N      -11.609135000     -3.697507000     21.952659000
O      -10.126175000     -4.622862000     20.517790000
C       -8.003013000     -9.808969000     15.431019000
C       -8.198214000     -8.520384000     16.204784000
C       -8.203027000     -7.267564000     15.546760000
C       -8.404742000     -8.517226000     17.601018000
C       -8.412324000     -6.071616000     16.247590000
C       -8.612456000     -7.325065000     18.320878000
C       -8.624762000     -6.090657000     17.641928000
O       -8.837322000     -4.888357000     18.273661000 newgto "def2-TZVP" end
Cu      -7.055567000     -3.399635000     19.166921000 newgto "def2-TZVP" end
H       -8.800822000     -2.413972000     14.029324000
H      -11.052882000     -5.470055000     17.280318000
H      -12.826044000     -5.513824000     17.051804000
H      -11.651535000     -3.171333000     17.890698000
H      -12.876471000     -3.080950000     16.587731000
H       -9.815510000     -3.037580000     16.342081000
H      -11.020947000     -2.037714000     15.457435000
H      -11.022756000     -6.163287000     15.024209000
H       -4.989181000     -4.462307000     27.713633000
```

```
H      -5.109034000     -2.763464000     27.104389000
H      -6.531555000     -4.255903000     25.876912000
H      -9.130443000     -4.554051000     27.824712000
H      -8.780918000     -6.239653000     27.314931000
H      -8.494278000     -2.664243000     25.795353000
H      -8.407205000     -2.852213000     23.257299000
H      -9.024329000     -5.282186000     22.487808000
H     -11.700791000     -7.317399000     20.785121000
H     -11.205932000     -6.708015000     19.174732000
H     -13.311997000     -5.326891000     21.009116000
H     -12.771854000     -4.735471000     19.424411000
H     -10.866725000     -3.160561000     22.409745000
H      -7.973962000    -10.685634000     16.106837000
H      -7.057576000     -9.792362000     14.849457000
H      -8.065805000     -7.222471000     14.454668000
H      -8.409472000     -9.474338000     18.146830000
H      -8.458828000     -5.109885000     15.717939000
H      -8.788476000     -7.354959000     19.406944000
H      -4.815395000     -4.460060000     18.569470000
H      -6.352860000     -4.561287000     16.066796000
H      -4.112063000     -4.938851000     15.011313000
H      -4.429576000     -2.379035000     17.057544000
H      -4.850337000     -2.583827000     15.341497000
H      -6.793320000     -0.608580000     14.755580000
H     -10.002080000      0.505367000     16.630795000
H      -8.685240000      1.386602000     15.769424000
H      -9.596236000      0.079895000     14.926896000
H      -4.653288000     -7.362142000     23.422025000
H      -5.846551000     -8.166259000     22.345921000
H      -6.641647000     -6.486465000     20.221938000
H      -5.491798000     -4.588020000     23.867069000
H      -6.039711000     -2.752262000     22.153784000
H      -3.313041000     -4.770557000     16.609764000
H      -4.415552000     -6.156009000     16.292458000
H      -6.025534000     -5.478808000     18.149508000
H      -3.759981000     -3.824716000     26.564030000
H      -6.383476000     -7.409159000     23.882547000
H     -10.439947000     -5.606007000     27.197683000
H     -12.548921000     -3.653091000     22.349005000
H     -12.887042000     -7.254404000     19.440937000
H     -12.701200000     -5.451111000     13.301273000
H     -13.489312000     -6.312342000     14.665993000
H     -13.609662000     -4.521634000     14.549523000
H      -9.059117000     -2.786891000     12.283699000
H     -10.294015000     -1.868376000     13.213656000
O      -8.435390000     -2.430065000     20.145162000 newgto "def2-TZVP" end
H      -9.042981000     -3.164293000     20.409785000
H      -8.825286000     -9.967012000     14.701332000
H      -9.368959000     -4.987521000     19.126602000
*
```

Selected results from calculations

E(High-Spin) = –3800.291578 Eh
E(BrokenSym) = –3800.297131 Eh
E(High-Spin)-E(BrokenSym)= 0.1511 eV 1218.559 cm^{-1} (ANTIFERROMAGNETIC coupling)

Distances/Å

Cu-OH	1.883
Cu-N (His1)	1.931
Cu-N (His2)	1.964
Cu-NH_2	2.040
Cu…OTyr	2.512

References:

(1) Frandsen, K. E. H.; Simmons, T. J.; Dupree, P.; Poulsen, J. N.; Hemsworth, G. R.; Ciano, L.; Johnston, E. M.; Tovborg, M.; Johansen, K. S.; Freiesleben, P. Von; Marmuse, L.; Fort, S.; Cottaz, S.; Driguez, H.; Henrissat, B.; Lenfant, N.; Tuna, F.; Baldansuren, A.; Davies, G. J.; Leggio, L. Lo; Walton, P. H. The Molecular Basis of Polysaccharide Cleavage by Lytic Polysaccharide Monooxygenases. *Nat. Chem. Biol.* **2016**, *12*, 298–303.

(2) Hernández-Ortega, A.; Quesne, M. G.; Bui, S.; Heyes, D. J.; Steiner, R. A.; Scrutton, N. S.; De Visser, S. P. Catalytic Mechanism of Cofactor-Free Dioxygenases and How They Circumvent Spin-Forbidden Oxygenation of Their Substrates. *J. Am. Chem. Soc.* **2015**, *137*, 7474–7487.

(3) Stoll, S.; Schweiger, A. EasySpin, a Comprehensive Software Package for Spectral Simulation and Analysis in EPR. *J. Magn. Reson.* **2006**, *178*, 42–55.

(4) Diaz-Moreno, S.; Amboage, M.; Basham, M.; Boada, R.; Bricknell, N. E.; Cibin, G.; Cobb, T. M.; Filik, J.; Freeman, A.; Geraki, K.; Gianolio, D.; Hayama, S.; Ignatyev, K.; Keenan, L.; Mikulska, I.; Mosselmans, J. F. W.; Mudd, J. J.; Parry, S. A. The Spectroscopy Village at Diamond Light Source. *J. Synchrotron Radiat.* **2018**, *25*, 998–1009.

(5) Hayama, S.; Duller, G.; Sutter, J. P.; Amboage, M.; Boada, R.; Freeman, A.; Keenan, L.; Nutter, B.; Cahill, L.; Leicester, P.; Kemp, B.; Rubies, N.; Diaz-Moreno, S. The Scanning Four-Bounce Monochromator for Beamline I20 at the Diamond Light Source. *J. Synchrotron Radiat.* **2018**, *25*, 1556–1564.

(6) Hayama, S.; Boada, R.; Chaboy, J.; Birt, A.; Duller, G.; Cahill, L.; Freeman, A.; Amboage, M.; Keenan, L.; Diaz-Moreno, S. Photon-in/Photon-out Spectroscopy at the I20-Scanning Beamline at Diamond Light Source. *J. Phys. Condens. Matter* **2021**, *33*.

(7) Johann, H. H. Die Erzeugung Lichtstarker Röntgenspektren Mit Hilfe von Konkavkristallen. **1931**, *185*, 185–206.

(8) Plackett, R.; Horswell, I.; Gimenez, E. N.; Marchal, J.; Omar, D.; Tartoni, N. Merlin: A Fast Versatile Readout System for Medipix3. *J. Instrum.* **2013**, *8*, 1–4.

(9) Ravel, B.; Newville, M. ATHENA, ARTEMIS, HEPHAESTUS: Data Analysis for X-Ray Absorption Spectroscopy Using IFEFFIT. *J. Synchrotron Radiat.* **2005**, *12*, 537–541.

(10) Neese, F. .; Wennmohs, F. .; Becker, U. .; Riplinger, C. The ORCA Quantum Chemistry Program Package. *J. Chem. Phys.* **2020**, *152*, 224108.

(11) Neese, F. Software Update: The ORCA Program System, Version 4.0. *Wiley Interdiscip. Rev. Comput. Mol. Sci.* **2018**, *8*, 1327–1333.

(12) Becke, A. D. Density-Fnnctional Exchange-Energy Approximation with Correct Asymptotic Behavior. *Phys. Rev. A* **1988**, *38*, 3098–3110.

(13) Weigend, F.; Ahlrichs, R. Balanced Basis Sets of Split Valence, Triple Zeta Valence and Quadruple Zeta Valence Quality for H to Rn: Design and Assessment of Accuracy. *Phys. Chem. Chem. Phys.* **2005**, *7*, 3297–3305.

(14) Grimme, S.; Ehrlich, S.; Goerigk, L. Effect of the Damping Function in Dispersion Corrected Density Functional Theory. *J. Comput. Chem.* **2011**, *32*, 1456–1465.

(15) Tomasi, J.; Mennucci, B.; Cammi, R. Quantum Mechanical Continuum Solvation Models. *Chem. Rev.* **2005**, *105*, 2999–3093.

(16) Becke, A. D. Density - Functional Thermochemistry. III. The Role of Exact Exchange. *J. Chem. Phys.* **1993**, *98*, 5648–5652.

(17) Soda, T.; Kitagawa, Y.; Onishi, T.; Takano, Y.; Shigeta, Y.; Nagao, H.; Yoshioka, Y.; Yamaguchi, K. Ab Initio Computations of Effective Exchange Integrals for H-H, H-He-H and Mn 2 O 2 Complex: Comparison of Broken-Symmetry Approaches. *Chem. Phys. Lett.* **2000**, *319*, 223–230.

(18) Hirata, S.; Head-Gordon, M. Time-Dependent Density Functional Theory within the Tamm-Dancoff Approximation. *Chem. Phys. Lett.* **1999**, *314*, 291–299.

(19) Neese, F.; Wennmohs, F.; Hansen, A.; Becker, U. Efficient, Approximate and Parallel Hartree-Fock and Hybrid DFT Calculations. A "chain-of-Spheres" Algorithm for the Hartree-Fock Exchange. *Chem. Phys.* **2009**, *356*, 98–109.

(20) Pantazis, D. A.; Chen, X. Y.; Landis, C. R.; Neese, F. All-Electron Scalar Relativistic Basis Sets for Third-Row Transition Metal Atoms. *J. Chem. Theory Comput.* **2008**, *4*, 908–919.

(21) Van Wüllen, C. Molecular Density Functional Calculations in the Regular Relativistic Approximation: Method, Application to Coinage Metal Diatomics, Hydrides, Fluorides and Chlorides, and Comparison with First-Order Relativistic Calculations. *J. Chem. Phys.* **1998**, *109*, 392–399.
